# Applicability Domain of Polyparameter Linear Free Energy Relationship Models Evaluated by Leverage and Prediction Interval Calculation

**DOI:** 10.1021/acs.est.2c00865

**Published:** 2022-04-14

**Authors:** Satoshi Endo

**Affiliations:** † Health and Environmental Risk Division, National Institute for Environmental Studies (NIES), Onogawa 16-2, Tsukuba, 305-8506 Ibaraki, Japan; ‡ Graduate School of Engineering, Osaka City University, Sugimoto 3-3-138, Sumiyoshi, 558-8585 Osaka, Japan

**Keywords:** applicability domain, linear solvation energy relationship, extrapolation, property prediction, partition coefficient, QSAR, QSPR, perfluoroalkyl substances

## Abstract

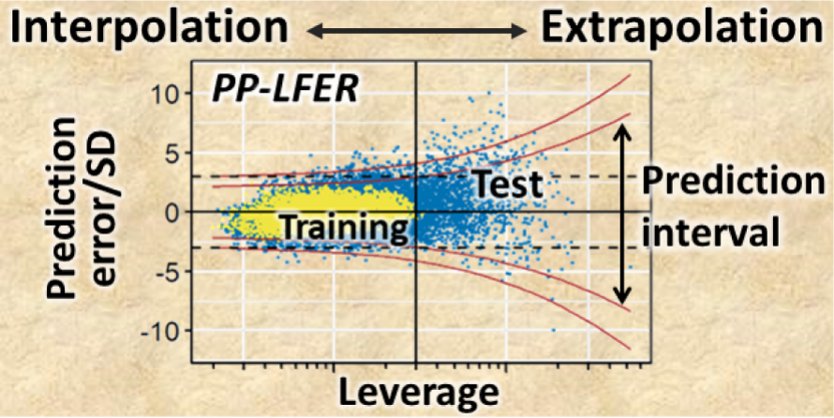

Polyparameter linear free energy relationships (PP-LFERs) are accurate and robust models employed to predict equilibrium partition coefficients (*K*) of organic chemicals. The accuracy of predictions by a PP-LFER depends on the composition of the respective calibration data set. Generally, extrapolation outside the domain defined by the calibration data is likely to be less accurate than interpolation. In this study, the applicability domain (AD) of PP-LFERs was systematically evaluated by calculating the leverage (*h*) and prediction interval (PI). Repeated simulations with experimental data showed that the root mean squared error of predictions increased with *h*. However, the analysis also showed that PP-LFERs calibrated with a large number (e.g., 100) of training data were highly robust against extrapolation error. For such PP-LFERs, the common definition of extrapolation (*h* > 3 *h*_mean_, where *h*_mean_ is the mean *h* of all training compounds) may be excessively strict. Alternatively, the PI is proposed as a metric to define the AD of PP-LFERs, as it provides a concrete estimate of the error range that agrees well with the observed errors, even for extreme extrapolations. Additionally, published PP-LFERs were evaluated in terms of their AD using the new concept of AD probes, which indicated the varying predictive performance of PP-LFERs in the existing literature for environmentally relevant compounds.

## Abstract 2

Calculating the prediction intervals delineates the applicability domain of polyparameter linear free energy relationship models.

## 1 Introduction

Equilibrium partition coefficients largely determine the environmental distribution of organic contaminants and are crucial parameters for environmental risk assessments. Among various models, the linear solvation energy relationships (LSERs)^1^ or, generally, polyparameter linear free energy relationships (PP-LFERs) that use Abraham’s solute descriptors have been confirmed to be accurate and robust for predicting partition coefficients.^2^ The PP-LFERs cover the intermolecular interactions relevant to the phase partitioning of neutral organic compounds. Their successful environmental applications have been previously reviewed.^3,4^

PP-LFERs are multiple linear regression models that typically use five solute descriptors. The following three types of equations are most often applied.^1,5^
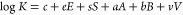
1
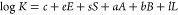
2
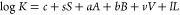
3

The symbols denote the following: *K*, partition coefficient; *E*, excess molar refraction; *S*, solute polarizability/dipolarity parameter; *A*, solute hydrogen (H)-bond donor property; *B*, solute H-bond acceptor property; *V*, McGowan’s molar volume; and *L*, logarithmic hexadecane/air partition coefficient. The lowercase letters are regression coefficients and are typically trained with several tens of compounds, for which experimental log *K* and the solute descriptors (i.e., *E*, *S*, *A*, *B*, *V*, and *L*) are available. The fitting of the PP-LFERs is high even to data that are highly diverse in size and polarity.^1,3^ For solvent/water and solvent/air partition coefficients, the calibration typically results in a standard deviation (SD) of 0.2 or below for the log *K* values.^1^ Partition systems that involve a heterogeneous phase (e.g., natural organic matter) can exhibit a lower quality of fit (SD, 0.3–0.5 log units).^3^

PP-LFERs are derived from a multiple linear regression; therefore, their applicability domain (AD) is related to the training (calibration) set of compounds. Generally, extrapolation (i.e., prediction beyond a specific domain defined by calibration data) is likely to be less accurate than interpolation. Moreover, a long-range extrapolation is expected to be more error-prone than a short-range extrapolation. However, in a multidimensional space (here, 5 descriptors), it is not straightforward to define the terms “interpolation” and “extrapolation” and to establish a quantitative relationship between the extent of extrapolation and prediction accuracy. Notably, an extrapolation can be less accurate but is not necessarily inaccurate or unreliable. The required accuracy depends on the purpose of the model use, and extrapolation can be acceptable within the range where its accuracy is satisfactory.

Among various approaches, the calculation of the leverages has been considered to define and evaluate the AD for linear regression models.^6−9^ The leverage is a quantitative measure of the distance from the entire set of calibration data. Leverage calculation is applied to identify outliers within the calibration set, and it can also be used to quantitatively define extrapolation in the prediction. A large leverage value indicates a long distance from the calibration data in terms of explanatory variables and thus an extrapolation with the possibility of increased error.

The prediction interval (PI) is the range of values where future data are expected to fall at a given frequency.^10^ Typically, 95% or 99% PIs are calculated. Although PIs are frequently calculated for predictions by a simple linear regression model, they are not commonly presented for multiple linear regression models, including PP-LFERs. However, the PI can be more useful than the leverage, as the PI considers both the distance from the calibration set and the quality of the model fitting (see Section 2.2 for more details).

The purposes of this study are threefold: (i) to quantitatively demonstrate how the prediction accuracy of a PP-LFER decreases when moving away from a specific domain of calibration defined by the leverage, (ii) to compare actual prediction errors with error margins expected by PIs, and (iii) to evaluate several calibration sets for PP-LFERs in terms of their AD using a new concept of AD probes. On the basis of these, a discussion is presented on the definition and evaluation of AD for PP-LFER models. The information should also be helpful for the future development of PP-LFERs because it ensures an optimized calibration data set.

## 2 Methodology

### 2.1 Definition and Calculation of the Leverage and PI

The definition and calculation of the leverage and PI are described in full in the Supporting Information and only briefly here.

The PP-LFER regression can be expressed in a matrix form as follows
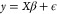
4where *y* is the vector of observations for log *K*, *β* is the vector of regression coefficients, *ε* is the error vector, and *X* is the design matrix consisting of a column of ones and the solute descriptors of *n* training compounds. The hat matrix (*H*) can be derived from *X*, and the diagonals of *H* (i.e., *h*_ii_) are referred to as the leverages and infer the distance of each calibration compound from the others in terms of the solute descriptor combination. *h*_ii_ is between 0 and 1, and the sum of *h*_ii_ for the *n* training compounds is equal to the number of fitting parameters *p*, which is 6 for the PP-LFERs (including the regression constant). An overly high *h*_ii_ indicates that the respective calibration compound is an outlier in terms of its descriptors. Typically, *h*_ii_ = 3*h*_mean_ is considered a threshold value,^6−9^ where *h*_mean_ is the mean of *h*_ii_ for all calibration compounds and is equal to *p*/*n*. To evaluate the extrapolation for compound *j*, which is not included in the calibration set, *h* is calculated as
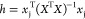
5where *x*_j_ is the column vector containing the solute descriptors of j. Analogous to the identification of outliers in the training set, *h* = 3*h*_mean_ is typically considered the threshold value for extrapolation.^6−9^

The PI of the PP-LFER can be expressed as [log *K*_j_ – Δ(log *K*), log *K*_j_ + Δ(log *K*)], where log *K*_j_ is the value of compound *j* predicted with eq 4 (i.e., log *K*_j_ = *x*_*j*_^T^ β) and Δ(log *K*) is half the width of the PI. Δ(log *K*) is calculated as^10^
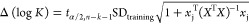
6
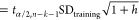
7where *t*_α/2,*n*–*k*–1_ is the two-tailed *t*-value for a given confidence level (α, e.g., 95%), number of training data (*n*), and number of independent variables (*k*; 5 for PP-LFERs). SD_training_ is the standard deviation of the PP-LFER model fitted to the training data. Δ(log *K*) may be normalized to SD_training_, as
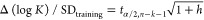
8

In this study, the following two tests were performed to discuss the use of *h* and the PIs to delineate the AD of PP-LFERs.

### 2.2 Test 1: Comparison of Prediction Errors with *h* and the PIs

In the first test, the variation in actual prediction errors by PP-LFERs with *h* and the PIs was examined. Six experimental data sets of partition coefficients from the existing literature were used: octanol/water (*K*_ow_, *n* = 314),^11^ air/water (*K*_aw_, *n* = 390),^12^ oil/water (*K*_oilw_, *n* = 247),^13^ soil organic carbon/water (*K*_oc_, *n* = 79),^14^ phospholipid liposome/water (*K*_lipw_, *n* = 131),^15^ and bovine serum albumin/water (*K*_BSAw_, *n* = 82).^16^ These data sets comprise a relatively large number of compounds and exhibit environmental and toxicological relevance. *K*_ow_, *K*_aw_, and *K*_oilw_ were partition coefficients between two homogeneous solvents, whereas *K*_oc_, *K*_lipw_, and *K*_BSAw_ involved a heterogeneous or anisotropic phase. The *K* values and solute descriptors were obtained from the aforecited references, are listed in Tables S1–S6, and are summarized in Table S7.

To evaluate the prediction accuracy, the *K* data of each set were divided into training and test sets. Training compounds were randomly selected from the entire data set. The number of the training compounds (*n*_training_) was 20, 30, 40, 50, 75, or 100. Rather small values of *n*_training_ were also included in this test to simulate cases of insufficient calibration. The compounds that were not selected as training compounds were used as test compounds. The PP-LFER in the form of eq 1 was calibrated with the training data and was used to predict log *K* for the test compounds. Prediction errors (predicted log *K* – experimental log *K*) were calculated and compared with *h* and Δ(log *K*). For each combination of the *K* set and *n*_training_, the cycle of “random generation of a training set”, “calibration of the PP-LFER”, and “prediction for the test set” was repeated 200 times. This number was arbitrary but appeared sufficient for stable results.

Additionally, using the 200 calibrated PP-LFERs for each case, the log *K* values of per- and polyfluoroalkyl substances (PFASs) and organosilicon compounds (OSCs) were predicted. PFASs and OSCs possess extremely weak van der Waals interaction properties; thus, the *E* and *L* values are comparatively low for their molecular sizes.^17,18^ Therefore, PP-LFERs have to be extrapolated to predict *K* values of PFASs and OSCs unless calibrated with these compounds.^18^ PFASs and OSCs are not present in the data set of any considered PP-LFER and are used to evaluate the influences of extrapolation on the prediction accuracy.

All calculations mentioned above were performed with *R* software.

### 2.3 Test 2: Evaluating Reported PP-LFERs with AD Probes

In the second test, *h* and PI calculation was applied to evaluate the AD of the reported PP-LFER equations. Here, *n*, SD_training_, and the solute descriptors of the calibration compounds were extracted from the existing literature and used to calculate *h* and PIs for 25 selected compounds (Table S8). These compounds, referred to as AD probes herein, were selected because of their wide variations in descriptor values, structural diversity, and environmental relevance. They represent aliphatic and aromatic compounds with varying molecular size and hydrogen (H)-bond interaction properties and include multifunctional polar compounds such as various pesticides and pharmaceuticals, a neutral PFAS, and an OSC. Experimental solute descriptors for the AD probes were obtained from the UFZ-LSER database and are listed in Table S8.^19^ Test 2 did not require the experimental *K* values of the AD probes, and only solute descriptors were used for the calculation. An Excel file with a macro is available on the Web that calculates *h*, *h*/*h*_mean_, and Δ(log *K*) for the AD probes and any desired chemical based on the user-entered training data (https://doi.org/10.26434/chemrxiv-2022-qs03q). Note that there exist compounds with extreme descriptor values that are not covered by the 25 AD probes proposed here. For example, an antibiotic erythromycin (*E* = 2.90, *S* = 3.73, *A* = 1.25, *B* = 4.96, *V* = 5.773)^20^ exhibits exceptionally high *S*, *B*, and *V* values. However, such compounds are rarely used for calibration and are thus highly likely to be out of the AD, which is clear without testing; therefore, compounds with extreme descriptor values were not included in the AD probe set.

## 3 Results and Discussion

### 3.1 Prediction Errors Compared to *h* and the PIs (Test 1)

Figure S1 shows the root mean squared errors (RMSEs) for training and testing sets randomly generated 200 times. The test compounds were grouped into several bins according to the *h* normalized to *h*_mean_ (*h*/*h*_mean_) before the RMSEs were calculated. The observed RMSE for the test compounds increased with *h* for a given *K* data set and *n*_training_. The increasing trend of RMSE with *h* was particularly clear for simulations with small *n*_training_ values (i.e., 20, 30). The increasing trend was sometimes unclear, or even an apparent decrease was seen (e.g., for log *K*_oilw_) for simulations with high *n*_training_ values in a high *h*/*h*_mean_ range, likely because a large *n*_training_ resulted in a relatively small *n*_test_, which may not be able to provide representative RMSEs, particularly for high *h*/*h*_mean_ bins. In other words, the sample size was sometimes too small to derive accurate RMSEs for high *h*/*h*_mean_ bins.

**Figure 1:**
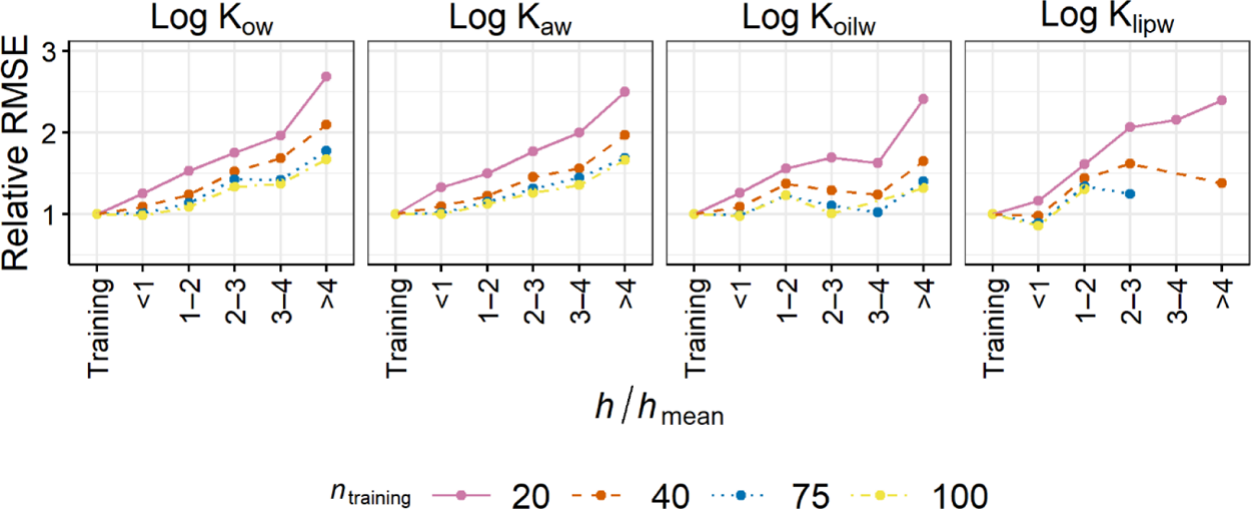
RMSEs of the test data, sorted according to *h*/*h*_mean_, relative to the RMSE of the training data. The plots for *n*_training_ = 30 and 50 and log *K*_oc_ and log *K*_BSAw_ are available in Figure S2.

To demonstrate the increase in RMSE with *h*/*h*_mean_ more clearly, the RMSE values for the test data relative to the RMSE for the training data were calculated (Figures 1 and S2). The relative RMSE generally increased with *h*/*h*_mean_ but to a lesser extent when *n*_training_ was large. For example, the relative RMSEs of log *K*_ow_ data in the “2 < *h*/*h*_mean_ < 3” bin were 1.75, 1.52, 1.42, and 1.34 for *n*_training_ = 20, 40, 75, and 100, respectively. This result suggests that if the PP-LFER is trained with a sufficient size of data, the RMSEs for interpolations (i.e., *h*/*h*_mean_ < 3) resemble the RMSE for the training set. Noteworthily, even for the “3 < *h*/*h*_mean_ < 4” bin (i.e., extrapolation), the relative RMSE for any *K* considered was <1.5 when *n*_training_ ≥ 50 and <2.2 when *n*_training_ ≥ 20. These RMSEs can be sufficiently accurate for various purposes. Although *h* > 3*h*_mean_ is the common definition of extrapolation, the actual threshold of *h* may be adapted to the required accuracy of predictions, depending on the quality of the PP-LFER fit and *n*_training_. For example, if the required accuracy is 0.3 log units, which is typically the level of accuracy of contaminant fate models,^21^ extrapolations by the PP-LFERs for log *K*_ow_ and log *K*_aw_ up to an *h*/*h*_mean_ of 4 can be allowed, according to the results of Test 1 (Figure S1). In contrast, a stricter criterion, for example, *h*/*h*_mean_ < 2 or even <1, should be set to log *K*_oc_, log *K*_lipw_, and log *K*_BSAw_ to comply with the criterion of 0.3 log unit RMSE. Alternative AD thresholds are further discussed in Section 3.3.

**Figure 2:**
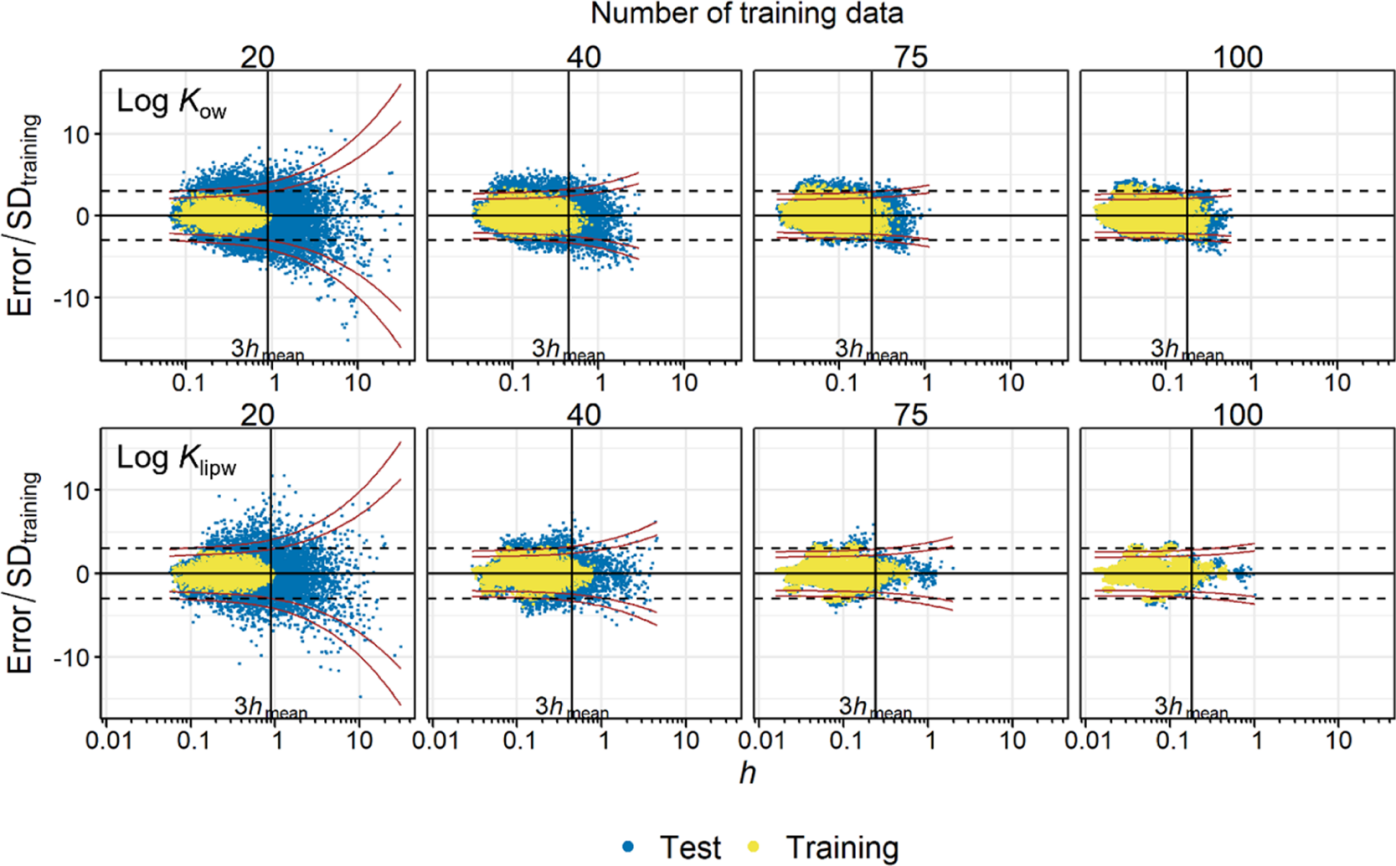
Prediction errors normalized to SD_training_ plotted against *h*. Results from 200 simulations are shown. The vertical line indicates 3*h*_mean_. The dashed horizontal lines indicate errors that are three times the SD_training_. The curves indicate the 95% (inside) and 99% (outside) prediction intervals. Top, log *K*_ow_; bottom, log *K*_lipw_. All other data are shown in Figure S3.

Along with average errors, such as RMSEs, the risk of an extremely inaccurate prediction is of interest. Individual data of Test 1 for log *K*_ow_ and log *K*_lipw_ were plotted against *h* (Figure 2). All other data are shown in Figure S3. When *n*_training_ was small (e.g., 20, 30), both *h* (*x*-axis) and prediction errors (*y*-axis, normalized to SD_training_) for the test data were widely distributed. Extremely large errors (|error/SD_training_| > 5) occasionally occurred, particularly if *h* was large (>10*h*_mean_). In contrast, when *n*_training_ was large (e.g., 75, 100), the training and test data were similarly distributed in terms of *h* and the prediction errors.

The percentage of large prediction errors, defined by |error/SD_training_| > 3, was generally higher for extrapolation (*h*/*h*_mean_ > 3) than interpolation (*h*/*h*_mean_ < 3) (Figure S4). However, the percentage strongly decreased with *n*_training_. As an example, for log *K*_ow_, when *n*_training_ = 20, 3.3% of the interpolations and 17% of the extrapolations suffered from large prediction errors. In contrast, when *n*_training_ = 100, 0.94% of the interpolations and 4.7% of the extrapolations resulted in large prediction errors, the latter conversely indicating that 95% of the extrapolations ended up with errors within 3 SD_training_.

Figure 2 additionally shows the 95% and 99% PIs as a function of *h*. The PIs were narrow up to *h* ∼ 1 and diverged with *h*, as expected from eq 8. The extent of divergence was large when *n*_training_ was small, which can be explained by a large *t*_α/2,*n*–*k*–1_ in eq 8. The data points from Test 1 were within the PIs with a few outliers. The percentage of the test data within a given PI agrees with the theoretical expectations; for example, ca. 95% of the test data are within the 95% PI, independent of *n*_training_ (Figure S5).

Overall, Test 1 demonstrated that *h* increased with the mean prediction error and could be used to identify “risky predictions” that frequently cause high inaccuracy. However, a threshold of 3*h*_mean_ did not appear to be versatile in defining the AD, as the *n*_training_ appeared to influence the range of prediction errors. The plots in Figures 1, 2, and S1–S5 suggest that, when *n*_training_ was large, *h* = 3*h*_mean_ might be overly strict as a threshold, because prediction errors were often similar in magnitude even when *h* > 3*h*_mean_. Note that Test 1 was also performed with eq 3, the PP-LFER equation that uses *L* instead of *E*. However, the results were similar to those of eq 1 and are thus not discussed herein.

### 3.2 PFASs and OSCs

**Figure 3:**
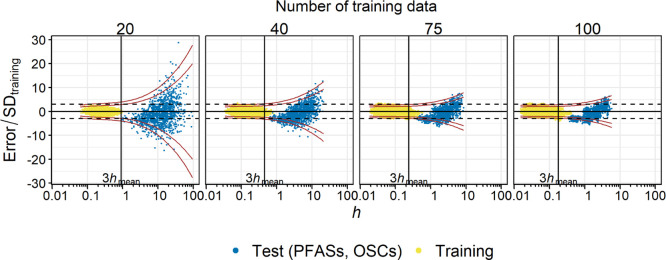
Prediction errors for log *K*_ow_ of PFASs and OSCs normalized to SD_training_ plotted against *h*. The results from 200 simulations are shown. The lines indicate the same as in Figure 2. Equation 3 was used for this plot (see text for more details). Additional data are in Figure S6.

Using 200 trained PP-LFERs, log *K*_ow_ of 3 PFASs [4:2 fluorotelomer alcohol (FTOH), 6:2 FTOH, and 8:2 FTOH] and 3 OSCs [octamethylcyclotetrasiloxane (D4), decamethylcyclopentasiloxane (D5), and dodecamethylcyclohexasiloxane (D6)] were predicted and compared to the experimental data (Figure 3; additional data in Figure S6).^18^ For this comparison, eq 3 instead of eq 1 was used because the latter is unsuitable for PFASs and OSCs (ref (18); also compare Figures S6 and S7). The *h*/*h*_mean_ ratios for these six chemicals were always above 3 with any *n*_training_ used and were up to 300, indicating strong extrapolations. The predictions were highly inaccurate when *n*_training_ was small. However, the predictions appeared to improve with an increase in *n*_training_. When *n*_training_ = 100, even largely extrapolated FTOHs (*h* ∼ 2, *h*/*h*_mean_ ∼ 33) were frequently predicted within 3 SD_training_. The dependence of the prediction error on *h* was well captured by the PIs; the majority of the data were within the 99% PIs, and this was the case for extreme extrapolations as well (Figures 3 and S6). The results for PFASs and OSCs can be considered another indication that well-calibrated PP-LFERs are robust against extrapolation and that *h* = 3*h*_mean_ as the cutoff is excessively strict if the *n*_training_ is large. Notably, although well-calibrated PP-LFERs appear to bear extrapolation, the inclusion of PFASs and OSCs in the calibration set is the first choice to develop PP-LFERs that work for these classes of chemicals, as that substantially decreases *h* for PFASs and OSCs.^18^

### 3.3 Defining the AD of PP-LFERs?

In previous discussions regarding the AD of quantitative structure activity relationships (QSARs), the use of *h* with a cutoff value of 3*h*_mean_ has been frequently presented. As shown in Test 1 of this study, however, this cutoff may excessively limit the potential of well-calibrated PP-LFERs to predict a broad range of compounds above the 3*h*_mean_ threshold. The use of the PI, in contrast, has rarely been investigated in the context of QSAR development but may be more practical for multiple linear regression models, such as PP-LFERs, because the PI encompasses the distance (*h*), quality of model fit (SD_training_), and size of training data (influencing *h* and *t*_α/2,*n*–*k*–1_) and provides a concrete estimate of the error range (eq 7). To use the PI to define the AD, an upper threshold for Δ(log *K*) must be set. Here, two ways that may be acceptable are discussed.

#### 3.3.1 Set the Δ(log *K*) Threshold at a Multiple of SD_training_

The AD may be defined by a Δ(log *K*) threshold that is a multiple of SD_training_. An example of such a criterion is Δ(log *K*)_99%PI_ < 3SD_training_. According to eq 8, this condition corresponds to
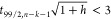
9

Inequality 9 describes the two intersections in Figures 2 and 3 where the curves for the 99% PI meet the horizontal lines for ±3SD_training_. By solving this inequality for *h*, we obtain
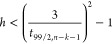
10

**Figure 4:**
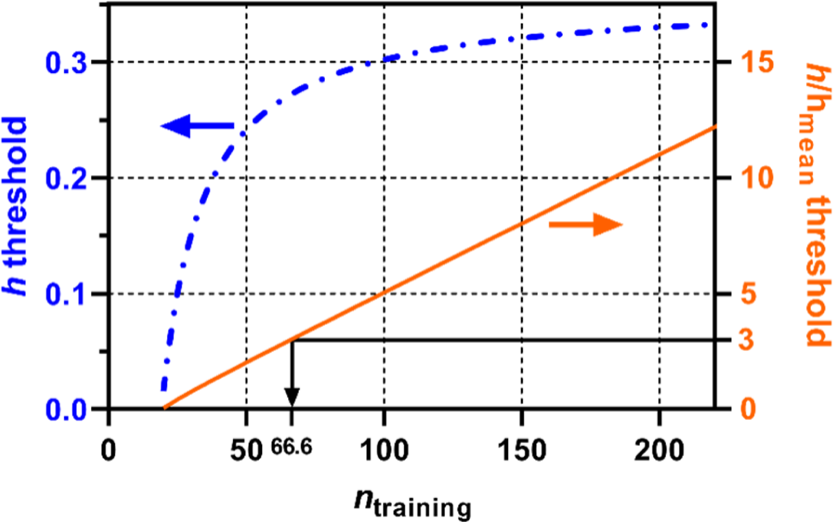
New thresholds of *h* (blue dash-dotted line) and *h*/*h*_mean_ (orange solid line) derived from the Δ(log *K*)_99%PI_ < 3SD_training_ criterion (eq 10) as a function of *n*_training_. The horizontal arrows indicate the axes that the data refer to. The *n*_training_ value that corresponds to the *h*/*h*_mean_ = 3 threshold is also indicated.

Inequality 10 describes a new *h* criterion that is derived from “Δ(log *K*)_99%PI_ < 3SD_training_“ and is a function of *t*_α/2,*n*–*k*–1_. As *t*_α/2,*n*–*k*–1_ is dependent on *n*_training_, this *h* threshold is also dependent on *n*_training_ (Figure 4). For example, if *n*_training_ = 50, the new threshold of *h* is 0.24, which corresponds to *h*/*h*_mean_ = 2.0. If *n*_training_ = 100, the threshold is *h* = 0.30, which is *h*/*h*_mean_ = 5.0. The common threshold of *h*/*h*_mean_ = 3 can be derived when *n*_training_ = 66.6. Thus, the new threshold is stricter if *n*_training_ ≤ 66 and less strict if *n*_training_ ≥ 67, compared with the 3*h*_mean_ rule.

#### 3.3.2 Set the Δ(log *K*) Threshold at a Certain Value

In the second approach, the AD is defined in such a way that the PI becomes narrower than a certain range. For example, we may consider Δ(log *K*)_99%PI_ < 0.5 (i.e., a factor of 3 for *K*) as an acceptable error margin; then, eq 7 becomes
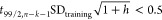
11which can be rewritten as
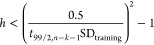
12

Using the SD_training_ value for the PP-LFER of log *K*_ow_ (Table S7) as an example, we can derive a threshold of *h* specific to log *K*_ow_. By inserting SD_training_ = 0.154 and *t*_99/2,*n*–*k*–1_ = 2.59 (with *n* = 314) in inequality 12, we obtained *h* < 0.57 (i.e., *h*/*h*_mean_ < 30) as the new criterion. Note that if SD_training_ is high (e.g., 0.285 for log *K*_lipw_), “Δ(log *K*)_99%PI_ < 0.5” is not achievable no matter how large *n*_training_ is, because *t*_99/2,*n*–*k*–1_ is >2.58 regardless of *n*_training_ and the right-hand side of inequality 12 is always negative. The difficulty associated with this approach to define the AD may be to set the acceptable Δ(log *K*)_99%PI_ level such that it is both useful and achievable.

### 3.4 Evaluating the AD of Published PP-LFERs with AD Probes (Test 2)

**Figure 5:**
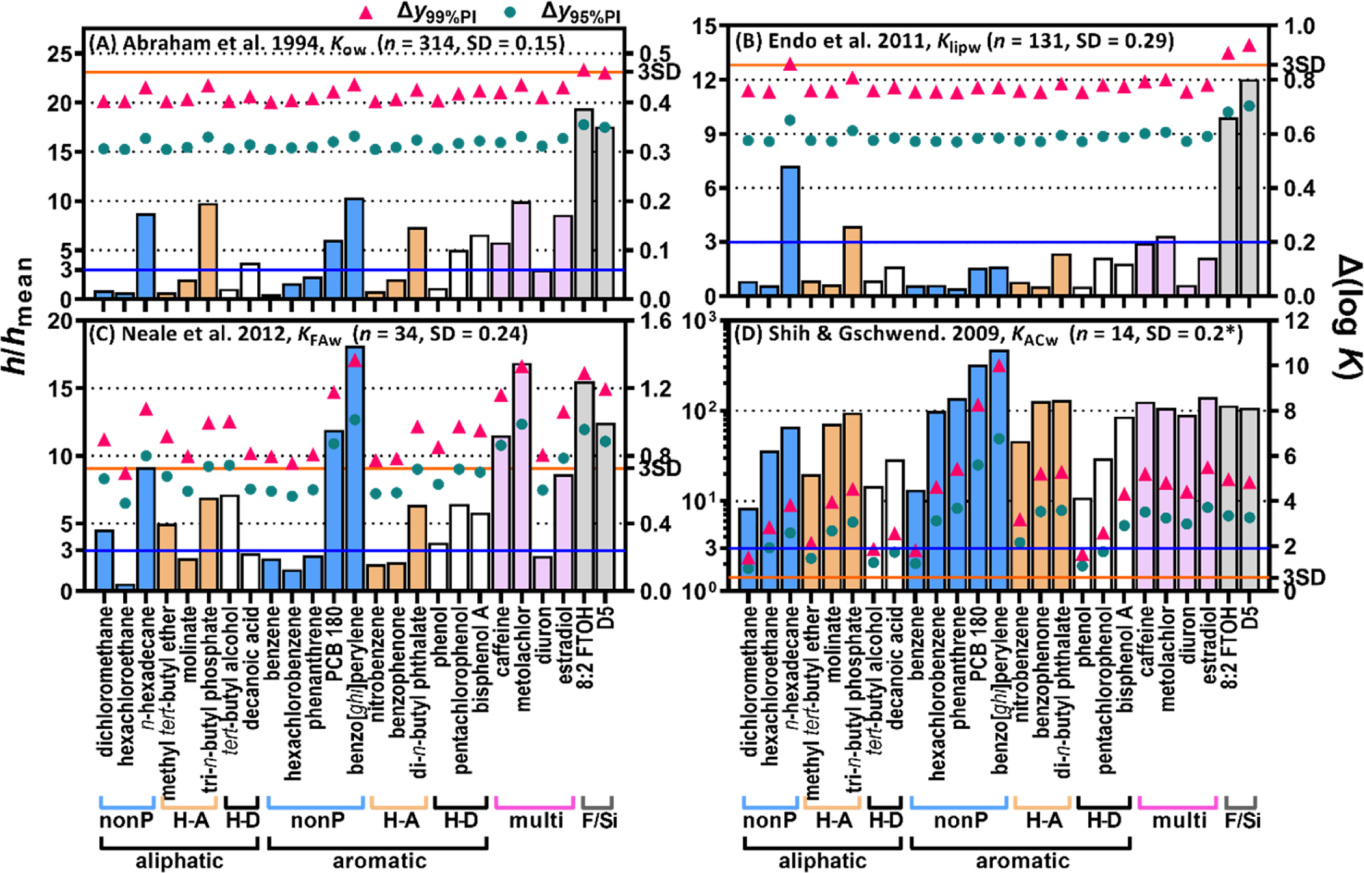
Leverage (bars) and prediction intervals (triangles and circles) of 25 applicability domain (AD) probes calculated with the training data sets of PP-LFERs for *K*_ow_, *K*_lipw_ (liposome/water), *K*_FAw_ (fulvic acid/water), and *K*_ACw_ (activated carbon/water). Solid horizontal lines indicate *h*/*h*_mean_ = 3 and Δ(log *K*) = 3SD. For convenience, chemicals are grouped, according to their structure and polarity, into nonpolar (nonP), H-bond acceptor (H-A), H-bond donor (H-D), multiple functional polar (multi), PFAS and OSC (F/Si), aliphatic, and aromatic chemicals. The cited reference does not give SD but a “mean error” of 0.2, which was used here (asterisked). Plots for all 10 PP-LFERs are shown in Figure S8.

Test 1 demonstrated the usefulness and limitations of *h* and the PI in evaluating the AD of PP-LFERs. In Test 2, *h* and the PI were applied to evaluate 10 published PP-LFER equations^11−16,22−25^ including those that had originally been derived from the data sets used in Test 1 (Figures 5 and S8). In this test, 25 environmentally relevant chemicals were considered as AD probes, as explained in the method section.

The *h* calculation showed that none of the 10 training sets considered encompassed all the 25 AD probes within the 3*h*_mean_ domain. This indicates that certain environmentally relevant compounds must be extrapolated with these PP-LFERs. Particularly, 8:2 FTOH and D5 always appeared as highly extrapolated chemicals (*h*/*h*_mean_ = 8–50), reflecting the fact that PFASs and OSCs were not included in any of the training sets and indicating that these compounds were not well represented by other training compounds. For each type of chemical, relatively small compounds (e.g., dichloromethane, methyl *tert*-butyl ether, and benzene) exhibited lower *h*/*h*_mean_ ratios than larger compounds (e.g., hexadecane, tri-*n*-butyl phosphate, and benzo[*ghi*]perylene). Generally, relatively small compounds are easy to measure, and their data are present in the training set, whereas obtaining data for large compounds tends to be more challenging.^26,27^ Consequently, PP-LFERs must be frequently extrapolated for large compounds.

The data sets for log *K*_ow_(11) and log *K*_aw_(12) exhibited similar patterns for *h*/*h*_mean_ and Δ(log *K*). Thus, the *h*/*h*_mean_ ratios of the small compounds were <3 (interpolation) and those of the large compounds were in the range of 3–15 (extrapolation) (Figure 5A). However, the Δ(log *K*) values were not largely different across the 25 AD probes. Although 12 out of 25 AD probes exhibited *h*/*h*_mean_ > 3, Δ(log *K*)_95%PI_ and Δ(log *K*)_99%PI_ were ∼0.3 and ∼0.4, respectively, for all the AD probes. Even for strongly extrapolated 8:2 FTOH, Δ(log *K*)_95%PI_ and Δ(log *K*)_99%PI_ of log *K*_ow_ predictions were 0.36 and 0.47, respectively. These relatively low Δ(log *K*) values for the extrapolated compounds originated from the low SD_training_ and the substantial size of the training data for *K*_ow_ and *K*_aw_. The log *K*_oilw_(13) data set resulted in similar patterns for *h*/*h*_mean_ and Δ(log *K*), but the values of Δ(log *K*) were higher than those of log *K*_ow_ and log *K*_aw_ because of the higher SD_training_ of log *K*_oilw_ (Figure S8).

The data set for log *K*_lipw_(15) had the benefit of excellent coverage of the AD probes; only 5 out of 25 AD probes exhibited *h*/*h*_mean_ > 3 (Figure 5B). A wealth of data for hydrophobic compounds (e.g., PAHs), substituted phenols, hormones, and pharmaceuticals in addition to simple aliphatic and aromatic and polar and nonpolar compounds with varying sizes resulted in low *h*/*h*_mean_ for the AD probes. Because of the low *h*/*h*_mean_ and high *n*, the Δ(log *K*) values were similar for all AD probes. Nevertheless, the values of Δ(log *K*)_95%PI_ and Δ(log *K*)_99%PI_ (∼0.6 and ∼0.8, respectively) for log *K*_lipw_ were higher than those for log *K*_ow_ by a factor of ∼2, because the SD_training_ of log *K*_lipw_ was higher by the same factor.

Figure 5C,D shows illustrative examples of PP-LFERs with limited training data. The data set of fulvic acid/water partition coefficients (*K*_FAw_)^22^ comprised 34 training data, and 16 out of 25 AD probes were extrapolated (*h*/*h*_mean_ > 3). The major difference from log *K*_ow_ and log *K*_lipw_ was the wide range of Δ(log *K*); the Δ(log *K*)_95%PI_ and Δ(log *K*)_99%PI_ values for log *K*_FAw_ were in the range of 0.5–1.0 and 0.7–1.4, respectively. The data set of activated carbon/water partition coefficients (*K*_ACw_)^25^ was a clearer example of insufficient calibration. It contained only 14 training data, and all AD probes were considered extrapolated (*h*/*h*_mean_, 8–480). Although the model fitting seemed to be good (stated mean error: 0.2),^25^ the PIs were extremely broad, with Δ(log *K*)_95%PI_ and Δ(log *K*)_99%PI_ being 1.0–6.8 and 1.5–10, respectively. These results indicate that PP-LFERs from such small training sets will have a limited predictive ability for external compounds. Conversely, the calculation of *h* and the PIs will be most useful for such poorly calibrated PP-LFERs, as they can identify compounds for which the precision of prediction is still acceptable.

In Supporting Information 10, a comparative discussion is provided for three data sets of log *K*_oc_(14,23,24) in terms of their ADs. These data sets comprise different calibration compounds and, accordingly, cover different types of compounds within their ADs, as demonstrated by the AD probes.

Overall, it can be concluded that the 25 AD probes are useful in illustrating the strength and weakness of calibrated PP-LFERs. The missing classes of compounds in the training data, for example, large hydrophobic compounds and multifunctional polar compounds, can be identified using the *h*/*h*_mean_ values, and the associated elevation of error margins can be evaluated by calculating the PIs. While 25 AD probes were exemplarily used in this study, other sets of AD probes could be also used with a larger or smaller number of chemicals or with specific chemicals of interest (e.g., pesticides), depending on the purpose of evaluation.

### 3.5 Practical Implications

This study demonstrated that extrapolation was error-prone when the number of training data was limited and the *h*/*h*_mean_ value was extremely high. In contrast, PP-LFERs calibrated with many training data (e.g., 100) were highly robust even when *h*/*h*_mean_ signified extrapolation. For partition coefficients between solvent phases or solvent and air such as *K*_ow_ and *K*_aw_, the data are typically accurate and abundant. Thus, extrapolations can frequently result in low prediction errors. Extrapolation is expected to cause unacceptable errors more often for heterogeneous environmental, biological, and technical phases, because the data are often limited, and SD_training_ tends to be large.

The commonly used threshold of *h* being 3 *h*_mean_ appeared to be not useful in defining the AD of PP-LFER models. Alternatively, two possible ways were proposed in this article to define the AD based on the calculation of the PIs. For practical purposes, presenting the PIs for each prediction may be highly recommended. For example, using the PP-LFER, log *K*_ow_ for hexachlorobenzene is predicted as 5.49 with a 95% PI of [5.16, 5.81]. With these PI values, the model user can appreciate the reliability of the prediction and decide whether the value is taken or not, following the accuracy required for the given model use. It could be claimed that calculating the PI each time is more important and useful than seeking a strict definition of the AD because the former presents a quantitative estimate of the error range, while the latter is a qualitative, binomial indicator with an arbitrary cutoff in the end.

To develop a robust PP-LFER, the training set should contain (i) a large number (>60, preferably >100) of (ii) accurate experimental *K* data for (iii) diverse compounds with (iv) accurate descriptors available. The reason is that (i) decreases *t*_α/2,*n*–*k*–1_ and *h*, (ii) and (iv) decrease SD_training_, and (iii) decreases *h* in eq 7, all contributing to tight PIs. The predictive performance of an empirical model is always restricted by the quality and quantity of the underlying experimental data. The improvement in data accuracy and availability will contribute to the further development of PP-LFER approaches.

Extended use of the PI may be considered for evaluating the AD of QSARs that are derived by the multiple linear regression analysis. The calculation of the PI is no more complex than *h* is, but the former provides far more insights into the reliability of predictions, as discussed above. Noteworthily, the success of applying the PIs for PP-LFERs stems from the excellent linear dependence of log *K* on the solute descriptors over a wide range, which is the premise of PP-LFER models such as eqs 1–3. This conversely means that, if the linear relationship between the solute descriptors and log *K* is weak (e.g., for complex phases), prediction errors can be larger and extreme outliers can occur more frequently than predicted by the PIs. The suitability of the PI for various partitioning phases and for various existing QSAR descriptors and properties warrants future investigation.

## Supporting Information Available

Additional explanations for *h* and PIs, used *K* data and AD probes, and additional figures for Tests 1 and 2 (PDF)

## References

[ref1] Abraham M. H.; Ibrahim A.; Zissimos A. M. Determination of sets of solute descriptors from chromatographic measurements. J. Chromatogr. A 2004, 1037, 29–47. 10.1016/j.chroma.2003.12.004. 15214659

[ref2] Goss K.-U.; Schwarzenbach R. P. Linear free energy relationships used to evaluate equilibrium partitioning of organic compounds. Environ. Sci. Technol. 2001, 35, 1–9. 10.1021/es000996d. 11351988

[ref3] Endo S.; Goss K.-U. Applications of polyparameter linear free energy relationships in environmental chemistry. Environ. Sci. Technol. 2014, 48, 12477–12491. 10.1021/es503369t. 25280011

[ref4] Poole C. F.; Ariyasena T. C.; Lenca N. Estimation of the environmental properties of compounds from chromatographic measurements and the solvation parameter model. J. Chromatogr. A 2013, 1317, 85–104. 10.1016/j.chroma.2013.05.045. 23768535

[ref5] Goss K.-U. Predicting the equilibrium partitioning of organic compounds using just one linear solvation energy relationship (LSER). Fluid Phase Equilib. 2005, 233, 19–22. 10.1016/j.fluid.2005.04.006.

[ref6] Netzeva T. I.; Worth A. P.; Aldenberg T.; Benigni R.; Cronin M. T. D.; Gramatica P.; Jaworska J. S.; Kahn S.; Klopman G.; Marchant C. A.; Myatt G.; Nikolova-Jeliazkova N.; Patlewicz G. Y.; Perkins R.; Roberts D. W.; Schultz T. W.; Stanton D. T.; van de Sandt J. J. M.; Tong W.; Veith G.; Yang C. Current status of methods for defining the applicability domain of (quantitative) structure-activity relationships. The report and recommendations of ECVAM Workshop 52. ATLA, Altern. Lab. Anim. 2005, 33, 155–173. 10.1177/026119290503300209. 16180989

[ref7] Jaworska J.; Nikolova-Jeliazkova N.; Aldenberg T. QSAR applicability domain estimation by projection of the training set in descriptor space: A review. ATLA, Altern. Lab. Anim. 2005, 33, 445–459. 10.1177/026119290503300508. 16268757

[ref8] Gramatica P. Principles of QSAR models validation: internal and external. QSAR Comb. Sci. 2007, 26, 694–701. 10.1002/qsar.200610151.

[ref9] Gramatica P.; Giani E.; Papa E. Statistical external validation and consensus modeling: A QSPR case study for K_oc_ prediction. J. Mol. Graph. Model. 2007, 25, 755–766. 10.1016/j.jmgm.2006.06.005. 16890002

[ref10] Myers R. H. Classical and Modern Regression with Applications, 2nd ed.; PWS Pub. Co., 1990.

[ref11] Abraham M. H.; Chadha H. S.; Whiting G. S.; Mitchell R. C. Hydrogen bonding. 32. An analysis of water-octanol and water-alkane partitioning and the Δlog P parameter of Seiler. J. Pharm. Sci. 1994, 83, 1085–1100. 10.1002/jps.2600830806. 7983591

[ref12] Abraham M. H.; Andonian-Haftvan J.; Whiting G. S.; Leo A.; Taft R. S. Hydrogen bonding. Part 34. The factors that influence the solubility of gases and vapors in water at 298 K, and a new method for its determination. J. Chem. Soc., Perkin Trans. 2 2 1994, 1777–1791. 10.1039/p29940001777.

[ref13] Geisler A.; Endo S.; Goss K.-U. Partitioning of organic chemicals to storage lipids: Elucidating the dependence on fatty acid composition and temperature. Environ. Sci. Technol. 2012, 46, 9519–9524. 10.1021/es301921w. 22849558

[ref14] Bronner G.; Goss K.-U. Predicting sorption of pesticides and other multifunctional organic chemicals to soil organic carbon. Environ. Sci. Technol. 2011, 45, 1313–1319. 10.1021/es102553y. 21194210

[ref15] Endo S.; Escher B. I.; Goss K.-U. Capacities of membrane lipids to accumulate neutral organic chemicals. Environ. Sci. Technol. 2011, 45, 5912–5921. 10.1021/es200855w. 21671592

[ref16] Endo S.; Goss K.-U. Serum albumin binding of structurally diverse neutral organic compounds: Data and models. Chem. Res. Toxicol. 2011, 24, 2293–2301. 10.1021/tx200431b. 22070391

[ref17] Goss K.-U.; Bronner G. What is so special about the sorption behavior of highly fluorinated compounds?. J. Phys. Chem. A 2006, 110, 9518–9522. 10.1021/jp062684o. 16869704

[ref18] Endo S.; Goss K.-U. Predicting partition coefficients of polyfluorinated and organosilicon compounds using polyparameter linear free energy relationships (PP-LFERs). Environ. Sci. Technol. 2014, 48, 2776–2784. 10.1021/es405091h. 24491038

[ref19] Ulrich N.; Endo S.; Brown T. N.; Watanabe N.; Bronner G.; Abraham M. H.; Goss K. U. UFZ-LSER database V 3.2, 2017. http://www.ufz.de/lserd, [Internet].

[ref20] Abraham M. H.; Ibrahim A.; Acree W. E. Jr. Air to lung partition coefficients for volatile organic compounds and blood to lung partition coefficients for volatile organic compounds and drugs. Eur. J. Med. Chem. 2008, 43, 478–485. 10.1016/j.ejmech.2007.04.002. 17544548

[ref21] Mackay D.; Arnot J. A. The application of fugacity and activity to simulating the environmental fate of organic contaminants. J. Chem. Eng. Data 2011, 56, 1348–1355. 10.1021/je101158y.

[ref22] Neale P. A.; Escher B. I.; Goss K.-U.; Endo S. Evaluating dissolved organic carbon–water partitioning using polyparameter linear free energy relationships: Implications for the fate of disinfection by-products. Water Res. 2012, 46, 3637–3645. 10.1016/j.watres.2012.04.005. 22542133

[ref23] Nguyen T. H.; Goss K.-U.; Ball W. P. Polyparameter linear free energy relationships for estimating the equilibrium partition of organic compounds between water and the natural organic matter in soils and sediments. Environ. Sci. Technol. 2005, 39, 913–924. 10.1021/es048839s. 15773462

[ref24] Endo S.; Grathwohl P.; Haderlein S. B.; Schmidt T. C. LFERs for soil organic carbon-water distribution coefficients (K_OC_) at environmentally relevant sorbate concentrations. Environ. Sci. Technol. 2009, 43, 3094–3100. 10.1021/es803157e. 19534119

[ref25] Shih Y.-h.; Gschwend P. M. Evaluating activated carbon–water sorption coefficients of organic compounds using a linear solvation energy relationship approach and sorbate chemical activities. Environ. Sci. Technol. 2009, 43, 851–857. 10.1021/es801663c. 19245026

[ref26] Li N.; Wania F.; Lei Y. D.; Daly G. L. A Comprehensive and critical compilation, evaluation, and selection of physical–chemical property data for selected polychlorinated biphenyls. J. Phys. Chem. Ref. Data 2003, 32, 1545–1590. 10.1063/1.1562632.

[ref27] Jonker M. T. O. Determining octanol-water partition coefficients for extremely hydrophobic chemicals by combining ″slow stirring″ and solid-phase microextraction. Environ. Toxicol. Chem. 2016, 35, 1371–1377. 10.1002/etc.3300. 26550770

