# Supplementary material for: Applicability Domain of Polyparameter Linear Free Energy Relationship Models Evaluated by Leverage and Prediction Interval Calculation
Source: Environ Sci Technol. 2022 Apr 14;56(9):5572–9. doi: 10.1021/acs.est.2c00865 (PMC9069697; doi:10.1021/acs.est.2c00865)
Supplement: Supplementary file 1 — es2c00865_si_001.pdf [file es2c00865_si_001.pdf]

# Electronic Supporting Information for "Applicability domain of polyparameter linear free energy relationship models evaluated by leverage and prediction interval calculation"

*Satoshi Endo*[1,2,*]

[1] Health and Environmental Risk Division, National Institute for Environmental Studies (NIES), Onogawa 16-2, 305-8506 Tsukuba, Ibaraki, Japan

[2] Graduate School of Engineering, Osaka City University, Sugimoto 3-3-138, Sumiyoshi, 558-8585 Osaka, Japan

*Corresponding author contact Information:

Satoshi Endo, Health and Environmental Risk Division, National Institute for Environmental Studies (NIES), Onogawa 16-2, 305-8506 Tsukuba, Ibaraki, Japan

Phone: ++81-29-850-2695, Fax: ++81-29-850-2870, Email: endo.satoshi@nies.go.jp

Contents

SI-1 Definition and calculation of the leverage and prediction interval

SI-2 Log *K* data and solute descriptors used in this study.

Table S1. Data and descriptors for log $K_{ow}$.

Table S2. Data and descriptors for log $K_{aw}$.

Table S3. Data and descriptors for log $K_{oilw}$.

Table S4. Data and descriptors for log $K_{oc}$.

Table S5. Data and descriptors for log $K_{lipw}$.

Table S6. Data and descriptors for log $K_{BSAw}$.

Table S7. Ranges of partition coefficients and solute descriptors (min/max) considered in this study.

SI-3 List of 25 applicability domain (AD) probes

Table S8. Twenty-five applicability domain (AD) probes used to test the reported PP-LFERs.

SI-4 RMSEs for training and test data (Test 1)

Figure S1. RMSEs for training and test data.

Figure S2. RMSEs of the test data, sorted according to $h/h_{mean}$, relative to the RMSE of the training data.

SI-5 Prediction errors normalized to $SD_{training}$ plotted against *h* (Test 1)

Figure S3 (A)–(F). Prediction errors normalized to $SD_{training}$ plotted against *h*.
SI-6 Percentage of large prediction errors in interpolation and extrapolation (Test 1)
Figure S4. Percentage of prediction outliers, as defined by $|error/SD_{training}| > 3$, in the 200 repeated simulations.
SI-7 Percentage of the test data for which predictions were within the given PIs (Test 1)
Figure S5. Percentage of the test data for which predictions were within the given PIs.
SI-8 Prediction errors for PFASs and OSCs with Equation 3 (*S*, *A*, *B*, *V*, *L*)
Figure S6 (A)–(F). Prediction errors for PFASs and OSCs normalized to $SD_{training}$ against *h* (eq 3).
SI-9 Prediction errors for PFASs and OSCs with Equation 1 (*E*, *S*, *A*, *B*, *V*)
Figure S7 (A)–(F). Prediction errors for PFASs and OSCs normalized to $SD_{training}$ against *h* (eq 1).
SI-10 Evaluation of literature PP-LFERs with leverages and prediction intervals for AD probes
References
Figure S8. Leverage (bars) and PIs (triangles and circles) of 25 AD probes calculated with the training data sets of 10 PP-LFERs in the literature.

## SI-1 Definition and calculation of the leverage and prediction interval

### Leverages ($h$)

A matrix expression of the PP-LFER regression is as follows,

$$y = X\beta + \varepsilon \qquad \text{(S1)}$$

where $y$ is the vector of observations for log $K$, $\beta$ is the vector of regression coefficients, and $\varepsilon$ is the error vector. $X$ is the design matrix consisting of a column of ones and the solute descriptors of $n$ training chemicals and is expressed as,

$$X = \begin{bmatrix} 1 & E_1 & S_1 & A_1 & B_1 & V_1 \\ \vdots & & \vdots & \vdots & & \vdots \\ 1 & E_n & S_n & A_n & B_n & V_n \end{bmatrix} \qquad \text{(S2)}$$

The PP-LFER in the form of eq 1 in the main manuscript was considered here as an example. The hat matrix ($H$) is defined as,

$$H = X\,(X^{\mathrm{T}} X)^{-1} X^{\mathrm{T}} \qquad \text{(S3)}$$

The diagonals of $H$ (i.e., $h_{ii}$) are referred to as the leverages and infer the distance of each calibration compound from the others in terms of the solute descriptor combination. $h_{ii}$ is constrained to between 0 and 1, and the sum of $h_{ii}$ for the $n$ training chemicals is equal to the number of fitting parameters $p$, which is 6 for the PP-LFERs (including the regression constant). Typically, $h_{ii} = 3h_{mean}$ is considered a threshold value,[1-4] where $h_{mean}$ is the mean of $h_{ii}$ for all calibration chemicals and is equal to $p/n$. An overly high $h_{ii}$ indicates that the respective calibration compound is an outlier in terms of its descriptors and that it strongly influences the regression coefficients; removing such a compound from the calibration set is advised.

To evaluate the extrapolation for compound $j$, which is not included in the calibration set, $h$ is calculated as,

$$h = x_j^{\mathrm{T}}\,(X^{\mathrm{T}} X)^{-1} x_j \qquad \text{(S4)}$$

where,

$$x_j = [1 \quad E_j \quad S_j \quad A_j \quad B_j \quad V_j]^{\mathrm{T}} \qquad \text{(S5)}$$

A high $h$ indicates that compound $j$ is distant from the calibration data set in terms of the descriptor

values and that the prediction for log $K_j$ by the trained model is an extrapolation. Analogous to the identification of outliers in the training set, $3h_{mean}$ is typically considered the threshold value for extrapolation.[1-4] However, the extent to which the prediction deteriorates above this threshold has not been investigated for PP-LFER models.

## Prediction intervals (PIs)

The PI of the PP-LFER can be expressed as [log $K_j$ – Δ(log *K*), log $K_j$ + Δ(log *K*)], where log $K_j$ is the value for compound *j* predicted with eq S1 (i.e., log $K_j = x_j^T$ β) and Δ(log *K*) is the estimated absolute error that delineates the PI. Δ(log *K*) is calculated as,

$$\Delta(\log K) = t_{\alpha/2,n-k-1}\mathrm{SD}_{\mathrm{training}}\sqrt{1 + x_{\mathrm{j}}^T(X^TX)^{-1}x_{\mathrm{j}}} \quad \text{(S6)}$$

$$= t_{\alpha/2,n-k-1}\mathrm{SD}_{\mathrm{training}}\sqrt{1+h} \quad \text{(S7)}$$

where $t_{\alpha/2,n\text{-}k\text{-}1}$ is the two-tailed *t*-value for a given confidence level (*α*, e.g., 95%), number of training data (*n*), and number of independent variables (*k*; 5 for PP-LFERs). $\mathrm{SD}_{\mathrm{training}}$ is the standard deviation of the PP-LFER model fitted to the training data. As obvious from eq 11, the PI widens with *h* and also with $\mathrm{SD}_{\mathrm{training}}$, the quality of the model fitting. Additionally, the PI is indirectly related to *n*, because *h* and $t_{\alpha/2,n\text{-}k\text{-}1}$ decrease with *n*. The PI normalized to $\mathrm{SD}_{\mathrm{training}}$ can be expressed as,

$$\Delta(\log K)/\mathrm{SD}_{\mathrm{training}} = t_{\alpha/2,n-k-1}\sqrt{1+h} \quad \text{(S8)}$$

Notably, $h > 0$ by definition, and when $h \ll 1$, Δ(log *K*) = $t_{\alpha/2,n\text{-k-}1}$ $\mathrm{SD}_{\mathrm{training}}$ and Δ(log *K*)/$\mathrm{SD}_{\mathrm{training}}$ = $t_{\alpha/2,n\text{-}k\text{-}1}$, representing the narrowest PIs for a given model.

## SI-2 Log *K* data and solute descriptors used in this study

Table S1. Data and descriptors for log $K_{ow}$.[5]

| | Log $K_{ow}$ | *E* | *S* | *A* | *B* | *V* | *L* |
|---|---|---|---|---|---|---|---|
| n-Pentane | 3.39 | 0.00 | 0.00 | 0.00 | 0.00 | 0.8131 | 2.162 |
| 2,2-Dimethylpropane | 3.11 | 0.00 | 0.00 | 0.00 | 0.00 | 0.8131 | 1.820 |
| n-Hexane | 3.90 | 0.00 | 0.00 | 0.00 | 0.00 | 0.9540 | 2.668 |
| 3-Methylpentane | 3.60 | 0.00 | 0.00 | 0.00 | 0.00 | 0.9540 | 2.581 |
| 2,2-Dimethylbutane | 3.82 | 0.00 | 0.00 | 0.00 | 0.00 | 0.9540 | 2.352 |
| 2,3-Dimethylbutane | 3.85 | 0.00 | 0.00 | 0.00 | 0.00 | 0.9540 | 2.495 |
| n-Heptane | 4.50 | 0.00 | 0.00 | 0.00 | 0.00 | 1.0949 | 3.173 |
| n-Octane | 5.15 | 0.00 | 0.00 | 0.00 | 0.00 | 1.2358 | 3.677 |
| n-Nonane | 5.65 | 0.00 | 0.00 | 0.00 | 0.00 | 1.3767 | 4.182 |
| Cyclopropane | 1.72 | 0.18 | 0.15 | 0.00 | 0.00 | 0.4227 | 1.314 |
| Cyclopentane | 3.00 | 0.26 | 0.10 | 0.00 | 0.00 | 0.7045 | 2.477 |
| Methylcyclopentane | 3.37 | 0.23 | 0.10 | 0.00 | 0.00 | 0.8454 | 2.907 |
| Cyclohexane | 3.44 | 0.31 | 0.10 | 0.00 | 0.00 | 0.8454 | 2.964 |
| Methylcyclohexane | 3.61 | 0.24 | 0.06 | 0.00 | 0.00 | 0.9863 | 3.319 |
| Propene | 1.77 | 0.10 | 0.08 | 0.00 | 0.07 | 0.4883 | 0.946 |
| But-1-ene | 2.40 | 0.10 | 0.08 | 0.00 | 0.07 | 0.6292 | 1.491 |
| Pent-1-ene | 2.80 | 0.09 | 0.08 | 0.00 | 0.07 | 0.7701 | 2.047 |
| 2-Methylbut-2-ene | 2.67 | 0.16 | 0.08 | 0.00 | 0.07 | 0.7701 | 2.226 |
| Hex-1-ene | 3.39 | 0.08 | 0.08 | 0.00 | 0.07 | 0.9110 | 2.572 |
| Hept-1-ene | 3.99 | 0.09 | 0.08 | 0.00 | 0.07 | 1.0519 | 3.063 |
| Oct-1-ene | 4.57 | 0.09 | 0.08 | 0.00 | 0.07 | 1.1928 | 3.568 |
| Non-1-ene | 5.15 | 0.09 | 0.08 | 0.00 | 0.07 | 1.3337 | 4.073 |
| Buta-1,3-diene | 1.99 | 0.32 | 0.23 | 0.00 | 0.10 | 0.5862 | 1.543 |
| Cyclohexene | 2.86 | 0.40 | 0.20 | 0.00 | 0.10 | 0.8024 | 3.021 |
| Cyclohepta-1,3,5-triene | 2.63 | 0.76 | 0.46 | 0.00 | 0.18 | 0.8573 | 3.442 |
| Propyne | 0.94 | 0.19 | 0.25 | 0.13 | 0.15 | 0.4453 | 1.025 |
| But-1-yne | 1.46 | 0.18 | 0.23 | 0.13 | 0.15 | 0.5862 | 1.520 |
| Pent-1-yne | 1.98 | 0.17 | 0.23 | 0.13 | 0.10 | 0.7271 | 2.010 |
| Hex-1-yne | 2.73 | 0.17 | 0.23 | 0.13 | 0.10 | 0.8680 | 2.510 |
| Hept-1-yne | 3.32 | 0.16 | 0.23 | 0.13 | 0.10 | 1.0089 | 3.000 |
| Oct-1-yne | 3.92 | 0.16 | 0.23 | 0.13 | 0.10 | 1.1498 | 3.521 |
| Tetrafluoromethane | 1.18 | -0.58 | -0.26 | 0.00 | 0.00 | 0.4003 | -0.817 |
| Chloromethane | 0.91 | 0.25 | 0.43 | 0.00 | 0.08 | 0.3719 | 1.163 |
| Dichloromethane | 1.25 | 0.39 | 0.57 | 0.10 | 0.05 | 0.4943 | 2.019 |
| Trichloromethane | 1.97 | 0.43 | 0.49 | 0.15 | 0.02 | 0.6167 | 2.480 |
| Tetrachloromethane | 2.83 | 0.46 | 0.38 | 0.00 | 0.00 | 0.7391 | 2.823 |
| Chloroethane | 1.43 | 0.23 | 0.40 | 0.00 | 0.10 | 0.5128 | 1.678 |
| 1,1-Dichloroethane | 1.79 | 0.32 | 0.49 | 0.10 | 0.10 | 0.6352 | 2.316 |
| 1,2-Dichloroethane | 1.48 | 0.42 | 0.64 | 0.10 | 0.11 | 0.6352 | 2.573 |
| 1,1,1-Trichloroethane | 2.49 | 0.37 | 0.41 | 0.00 | 0.09 | 0.7576 | 2.733 |
| 1,1,2-Trichloroethane | 1.89 | 0.50 | 0.68 | 0.13 | 0.08 | 0.7576 | 3.290 |
| 1,1,2,2-Tetrachloroethane | 2.39 | 0.60 | 0.76 | 0.16 | 0.12 | 0.8800 | 3.803 |
| 1,1,1,2-Tetrachloroethane | 2.66 | 0.54 | 0.63 | 0.10 | 0.08 | 0.8800 | 3.641 |
| Pentachloroethane | 3.22 | 0.65 | 0.66 | 0.17 | 0.06 | 1.0024 | 4.267 |
| 1-Chloropropane | 2.04 | 0.22 | 0.40 | 0.00 | 0.10 | 0.6537 | 2.202 |
| 2-Chloropropane | 1.90 | 0.18 | 0.35 | 0.00 | 0.12 | 0.6537 | 1.970 |
| 1,2-Dichloropropane | 2.02 | 0.37 | 0.60 | 0.10 | 0.11 | 0.7761 | 2.857 |
| 1,3-Dichloropropane | 2.00 | 0.41 | 0.74 | 0.00 | 0.17 | 0.7761 | 3.101 |
| 1-Chlorobutane | 2.64 | 0.21 | 0.40 | 0.00 | 0.10 | 0.7946 | 2.722 |
| 2-Chlorobutane | 2.52 | 0.19 | 0.35 | 0.00 | 0.12 | 0.7946 | 2.540 |
| 2-Chloro-2-methylpropane | 2.39 | 0.14 | 0.25 | 0.00 | 0.12 | 0.7946 | 2.217 |

| | | | | | | | |
|---|---|---|---|---|---|---|---|
| 1-Chloropentane | 3.11 | 0.21 | 0.40 | 0.00 | 0.10 | 0.9355 | 3.223 |
| 1-Chlorohexane | 3.66 | 0.20 | 0.40 | 0.00 | 0.10 | 1.0764 | 3.777 |
| 1-Chloroheptane | 4.15 | 0.19 | 0.40 | 0.00 | 0.10 | 1.2173 | 4.282 |
| 1,1-Dichloroethene | 2.13 | 0.36 | 0.34 | 0.00 | 0.05 | 0.5922 | 2.110 |
| (Z)-1,2-Dichloroethene | 1.86 | 0.44 | 0.61 | 0.11 | 0.05 | 0.5922 | 2.439 |
| (E)-1,2-Dichloroethene | 2.09 | 0.43 | 0.41 | 0.09 | 0.05 | 0.5922 | 2.278 |
| Trichloroethene | 2.42 | 0.52 | 0.37 | 0.08 | 0.03 | 0.7146 | 2.997 |
| Tetrachloroethene | 3.40 | 0.64 | 0.44 | 0.00 | 0.00 | 0.8370 | 3.584 |
| Bromomethane | 1.19 | 0.40 | 0.43 | 0.00 | 0.10 | 0.4245 | 1.630 |
| Tribromomethane | 2.67 | 0.97 | 0.68 | 0.15 | 0.06 | 0.7745 | 3.784 |
| Bromoethane | 1.61 | 0.37 | 0.40 | 0.00 | 0.12 | 0.5654 | 2.120 |
| 1-Bromopropane | 2.10 | 0.37 | 0.40 | 0.00 | 0.12 | 0.7063 | 2.620 |
| 2-Bromopropane | 2.10 | 0.33 | 0.35 | 0.00 | 0.14 | 0.7063 | 2.390 |
| 1-Bromobutane | 2.75 | 0.36 | 0.40 | 0.00 | 0.12 | 0.8472 | 3.105 |
| 1-Bromo-2-methylpropane | 2.53 | 0.34 | 0.37 | 0.00 | 0.12 | 0.8472 | 2.960 |
| 2-Bromo-2-methylpropane | 2.53 | 0.31 | 0.25 | 0.00 | 0.14 | 0.8472 | 2.616 |
| 1-Bromopentane | 3.37 | 0.36 | 0.40 | 0.00 | 0.12 | 0.9881 | 3.611 |
| 1-Bromohexane | 3.80 | 0.35 | 0.40 | 0.00 | 0.12 | 1.1290 | 4.130 |
| 1-Bromoheptane | 4.36 | 0.34 | 0.40 | 0.00 | 0.12 | 1.2699 | 4.663 |
| 1-Bromooctane | 4.89 | 0.34 | 0.40 | 0.00 | 0.12 | 1.4108 | 5.000 |
| Iodomethane | 1.51 | 0.68 | 0.43 | 0.00 | 0.13 | 0.5077 | 2.106 |
| Iodoethane | 2.00 | 0.64 | 0.40 | 0.00 | 0.15 | 0.6486 | 2.573 |
| 1-Iodopropane | 2.54 | 0.63 | 0.40 | 0.00 | 0.14 | 0.7895 | 3.130 |
| 1-Iodobutane | 3.08 | 0.63 | 0.40 | 0.00 | 0.15 | 0.9304 | 3.628 |
| 1-Iodopentane | 3.62 | 0.62 | 0.40 | 0.00 | 0.15 | 1.0713 | 4.130 |
| 1-Iodohexane | 4.16 | 0.62 | 0.40 | 0.00 | 0.15 | 1.2122 | 4.620 |
| 1-Bromo-1-chloro-2,2,2-trifluoroethane | 2.30 | 0.10 | 0.39 | 0.13 | 0.05 | 0.8009 | 1.982 |
| 1-Bromo-1,2,2,2-tetrafluoroethane | 2.01 | -0.07 | 0.21 | 0.20 | 0.00 | 0.7162 | 1.370 |
| Diethyl ether | 0.89 | 0.04 | 0.25 | 0.00 | 0.45 | 0.7309 | 2.015 |
| Di-n-propyl ether | 2.03 | 0.01 | 0.25 | 0.00 | 0.45 | 1.0127 | 2.954 |
| Di-n-butyl ether | 3.21 | 0.00 | 0.25 | 0.00 | 0.45 | 1.2945 | 3.924 |
| Diisopropyl ether | 2.03 | -0.06 | 0.16 | 0.00 | 0.58 | 1.0127 | 2.530 |
| Methoxyflurane | 2.21 | 0.11 | 0.67 | 0.07 | 0.14 | 0.9102 | 2.864 |
| Isoflurane | 2.06 | -0.24 | 0.56 | 0.00 | 0.08 | 0.9009 | 1.969 |
| Tetrahydrofuran | 0.46 | 0.29 | 0.52 | 0.00 | 0.48 | 0.6223 | 2.636 |
| 2,5-Dimethyltetrahydrofuran | 1.25 | 0.20 | 0.38 | 0.00 | 0.58 | 0.9041 | 2.980 |
| Tetrahydropyran | 0.95 | 0.28 | 0.47 | 0.00 | 0.55 | 0.7632 | 3.057 |
| 1,4-Dioxane | -0.27 | 0.33 | 0.75 | 0.00 | 0.64 | 0.6810 | 2.892 |
| Butyraldehyde | 0.88 | 0.19 | 0.65 | 0.00 | 0.45 | 0.6879 | 2.270 |
| Hexanal | 1.78 | 0.15 | 0.65 | 0.00 | 0.45 | 0.9697 | 3.357 |
| 2-Propanone | -0.24 | 0.18 | 0.70 | 0.04 | 0.49 | 0.5470 | 1.696 |
| 2-Butanone | 0.29 | 0.17 | 0.70 | 0.00 | 0.51 | 0.6879 | 2.287 |
| Pentan-2-one | 0.91 | 0.14 | 0.68 | 0.00 | 0.51 | 0.8288 | 2.755 |
| Pentan-3-one | 0.82 | 0.15 | 0.66 | 0.00 | 0.51 | 0.8288 | 2.811 |
| 3-Methylbutan-2-one | 0.84 | 0.13 | 0.65 | 0.00 | 0.51 | 0.8288 | 2.692 |
| Hexan-2-one | 1.38 | 0.14 | 0.68 | 0.00 | 0.51 | 0.9697 | 3.286 |
| 4-Methylpentan-2-one | 1.31 | 0.11 | 0.65 | 0.00 | 0.51 | 0.9697 | 3.089 |
| Heptan-2-one | 1.98 | 0.12 | 0.68 | 0.00 | 0.51 | 1.1106 | 3.760 |
| Heptan-4-one | 2.04 | 0.11 | 0.66 | 0.00 | 0.51 | 1.1106 | 3.705 |
| Octan-2-one | 2.37 | 0.11 | 0.68 | 0.00 | 0.51 | 1.2515 | 4.257 |
| Nonan-2-one | 3.14 | 0.12 | 0.68 | 0.00 | 0.51 | 1.3924 | 4.735 |
| Nonan-5-one | 2.88 | 0.10 | 0.66 | 0.00 | 0.51 | 1.3924 | 4.698 |
| Decan-2-one | 3.73 | 0.11 | 0.68 | 0.00 | 0.51 | 1.5333 | 5.245 |
| Undecan-2-one | 4.09 | 0.10 | 0.68 | 0.00 | 0.51 | 1.6742 | 5.732 |
| Cyclopentanone | 0.38 | 0.37 | 0.86 | 0.00 | 0.52 | 0.7202 | 3.221 |
| Cyclohexanone | 0.81 | 0.40 | 0.86 | 0.00 | 0.56 | 0.8611 | 3.792 |
| Methyl formate | -0.26 | 0.19 | 0.68 | 0.00 | 0.38 | 0.4648 | 1.285 |

| | | | | | | | |
|---|---|---|---|---|---|---|---|
| Ethyl formate | 0.27 | 0.15 | 0.66 | 0.00 | 0.38 | 0.6057 | 1.845 |
| n-Propyl formate | 0.83 | 0.13 | 0.63 | 0.00 | 0.38 | 0.7466 | 2.433 |
| Methyl acetate | 0.18 | 0.14 | 0.64 | 0.00 | 0.45 | 0.6057 | 1.911 |
| Ethyl acetate | 0.73 | 0.11 | 0.62 | 0.00 | 0.45 | 0.7466 | 2.314 |
| n-Propyl acetate | 1.24 | 0.09 | 0.60 | 0.00 | 0.45 | 0.8875 | 2.819 |
| Isopropyl acetate | 1.02 | 0.06 | 0.57 | 0.00 | 0.47 | 0.8875 | 2.546 |
| n-Butyl acetate | 1.78 | 0.07 | 0.60 | 0.00 | 0.45 | 1.0284 | 3.353 |
| Isobutyl acetate | 1.78 | 0.05 | 0.57 | 0.00 | 0.47 | 1.0284 | 3.161 |
| n-Pentyl acetate | 2.30 | 0.07 | 0.60 | 0.00 | 0.45 | 1.1693 | 3.844 |
| Isoamyl acetate | 2.17 | 0.05 | 0.57 | 0.00 | 0.47 | 1.1693 | 3.740 |
| n-Hexyl acetate | 2.83 | 0.06 | 0.60 | 0.00 | 0.45 | 1.3102 | 4.290 |
| Methyl propanoate | 0.82 | 0.13 | 0.60 | 0.00 | 0.45 | 0.7466 | 2.431 |
| Ethyl propanoate | 1.21 | 0.09 | 0.58 | 0.00 | 0.45 | 0.8875 | 2.807 |
| n-Propyl propanoate | 1.71 | 0.07 | 0.56 | 0.00 | 0.45 | 1.0284 | 3.338 |
| n-Pentyl propanoate | 2.67 | 0.05 | 0.56 | 0.00 | 0.45 | 1.3102 | 4.331 |
| Methyl butanoate | 1.29 | 0.11 | 0.60 | 0.00 | 0.45 | 0.8875 | 2.893 |
| Ethyl butanoate | 1.71 | 0.07 | 0.58 | 0.00 | 0.45 | 1.0284 | 3.271 |
| Propyl butanoate | 2.15 | 0.05 | 0.56 | 0.00 | 0.45 | 1.1693 | 3.783 |
| Methyl pentanoate | 1.96 | 0.11 | 0.60 | 0.00 | 0.45 | 1.0284 | 3.392 |
| Ethyl pentanoate | 2.30 | 0.05 | 0.58 | 0.00 | 0.45 | 1.1693 | 3.769 |
| Methyl hexanoate | 2.42 | 0.08 | 0.60 | 0.00 | 0.45 | 1.1693 | 3.874 |
| Acetonitrile | -0.34 | 0.24 | 0.90 | 0.04 | 0.33 | 0.4042 | 1.739 |
| Propionitrile | 0.16 | 0.16 | 0.90 | 0.02 | 0.36 | 0.5451 | 2.082 |
| butyronitrile | 0.53 | 0.19 | 0.90 | 0.00 | 0.36 | 0.6860 | 2.548 |
| n-Valeronitrile | 1.12 | 0.18 | 0.90 | 0.00 | 0.36 | 0.8269 | 3.108 |
| Methylamine | -0.57 | 0.25 | 0.35 | 0.16 | 0.58 | 0.3493 | 1.300 |
| Ethylamine | -0.13 | 0.24 | 0.35 | 0.16 | 0.61 | 0.4902 | 1.677 |
| n-Propylamine | 0.48 | 0.23 | 0.35 | 0.16 | 0.61 | 0.6311 | 2.141 |
| n-Butylamine | 0.97 | 0.22 | 0.35 | 0.16 | 0.61 | 0.7720 | 2.618 |
| n-Pentylamine | 1.49 | 0.21 | 0.35 | 0.16 | 0.61 | 0.9129 | 3.139 |
| n-Hexylamine | 2.06 | 0.20 | 0.35 | 0.16 | 0.61 | 1.0538 | 3.655 |
| n-Heptylamine | 2.57 | 0.20 | 0.35 | 0.16 | 0.61 | 1.1947 | 4.166 |
| n-Octylamine | 3.09 | 0.19 | 0.35 | 0.16 | 0.61 | 1.3356 | 4.520 |
| Cyclohexylamine | 1.49 | 0.33 | 0.56 | 0.16 | 0.58 | 0.9452 | 3.796 |
| Dimethylamine | -0.38 | 0.19 | 0.30 | 0.08 | 0.66 | 0.4902 | 1.600 |
| Diethylamine | 0.58 | 0.15 | 0.30 | 0.08 | 0.68 | 0.7720 | 2.395 |
| Di-n-propylamine | 1.53 | 0.12 | 0.30 | 0.08 | 0.68 | 1.0538 | 3.351 |
| Diisopropylamine | 1.16 | 0.05 | 0.24 | 0.08 | 0.71 | 1.0538 | 2.893 |
| Di-n-butylamine | 2.83 | 0.11 | 0.30 | 0.08 | 0.68 | 1.3356 | 4.349 |
| Trimethylamine | 0.22 | 0.14 | 0.20 | 0.00 | 0.67 | 0.6311 | 1.620 |
| Triethylamine | 1.45 | 0.10 | 0.15 | 0.00 | 0.79 | 1.0538 | 3.040 |
| Nitromethane | -0.35 | 0.31 | 0.95 | 0.06 | 0.31 | 0.4237 | 1.892 |
| Nitroethane | 0.18 | 0.27 | 0.95 | 0.02 | 0.33 | 0.5646 | 2.414 |
| 1-Nitropropane | 0.87 | 0.24 | 0.95 | 0.00 | 0.31 | 0.7055 | 2.894 |
| 2-Nitropropane | 0.55 | 0.22 | 0.92 | 0.00 | 0.32 | 0.7055 | 2.550 |
| 1-Nitrobutane | 1.47 | 0.23 | 0.95 | 0.00 | 0.29 | 0.8464 | 3.415 |
| 1-Nitropentane | 2.01 | 0.21 | 0.95 | 0.00 | 0.29 | 0.9873 | 3.938 |
| Dimethylformamide | -1.01 | 0.37 | 1.31 | 0.00 | 0.74 | 0.6468 | 3.173 |
| Acetic acid | -0.17 | 0.27 | 0.64 | 0.62 | 0.44 | 0.4648 | 1.816 |
| Propanoic acid | 0.33 | 0.23 | 0.65 | 0.60 | 0.45 | 0.6057 | 3.024 |
| Butanoic acid | 0.79 | 0.21 | 0.62 | 0.60 | 0.45 | 0.7466 | 3.473 |
| Pentanoic acid | 1.39 | 0.21 | 0.60 | 0.60 | 0.45 | 0.8875 | 3.969 |
| 3-Methylbutanoic acid | 1.16 | 0.18 | 0.57 | 0.60 | 0.50 | 0.8875 | 3.140 |
| Hexanoic acid | 1.92 | 0.17 | 0.60 | 0.60 | 0.45 | 1.0284 | 3.920 |
| Water | -1.38 | 0.00 | 0.45 | 0.82 | 0.35 | 0.1673 | 0.260 |
| Methanol | -0.74 | 0.28 | 0.44 | 0.43 | 0.47 | 0.3082 | 0.970 |
| Ethanol | -0.30 | 0.25 | 0.42 | 0.37 | 0.48 | 0.4491 | 1.485 |

| | | | | | | | |
|---|---|---|---|---|---|---|---|
| Propan-1-ol | 0.25 | 0.24 | 0.42 | 0.37 | 0.48 | 0.5900 | 2.031 |
| Propan-2-ol | 0.05 | 0.21 | 0.36 | 0.33 | 0.56 | 0.5900 | 1.764 |
| Butan-1-ol | 0.88 | 0.22 | 0.42 | 0.37 | 0.48 | 0.7309 | 2.601 |
| 2-Methylpropan-1-ol | 0.76 | 0.22 | 0.39 | 0.37 | 0.48 | 0.7309 | 2.413 |
| Butan-2-ol | 0.61 | 0.22 | 0.36 | 0.33 | 0.56 | 0.7309 | 2.338 |
| 2-Methylpropan-2-ol | 0.35 | 0.28 | 0.30 | 0.31 | 0.60 | 0.7309 | 1.963 |
| Pentan-1-ol | 1.56 | 0.22 | 0.42 | 0.37 | 0.48 | 0.8718 | 3.106 |
| Pentan-2-ol | 1.19 | 0.20 | 0.36 | 0.33 | 0.56 | 0.8718 | 2.840 |
| Pentan-3-ol | 1.21 | 0.22 | 0.36 | 0.33 | 0.56 | 0.8718 | 2.860 |
| 2-Methylbutan-1-ol | 1.16 | 0.22 | 0.39 | 0.37 | 0.48 | 0.8718 | 3.011 |
| 3-Methylbutan-1-ol | 1.16 | 0.19 | 0.39 | 0.37 | 0.48 | 0.8718 | 3.011 |
| 2-Methylbutan-2-ol | 0.89 | 0.19 | 0.30 | 0.31 | 0.60 | 0.8718 | 2.630 |
| Hexan-1-ol | 2.03 | 0.21 | 0.42 | 0.37 | 0.48 | 1.0127 | 3.610 |
| Hexan-3-ol | 1.65 | 0.20 | 0.36 | 0.33 | 0.56 | 1.0127 | 3.343 |
| 2-Methylpentan-2-ol | 1.53 | 0.17 | 0.30 | 0.31 | 0.60 | 1.0127 | 3.081 |
| Heptan-1-ol | 2.72 | 0.21 | 0.42 | 0.37 | 0.48 | 1.1536 | 4.115 |
| Octan-1-ol | 3.07 | 0.20 | 0.42 | 0.37 | 0.48 | 1.2945 | 4.619 |
| Nonan-1-ol | 3.67 | 0.19 | 0.42 | 0.37 | 0.48 | 1.4354 | 5.124 |
| Cyclopentanol | 0.71 | 0.43 | 0.54 | 0.32 | 0.56 | 0.7632 | 3.241 |
| Cyclohexanol | 1.23 | 0.46 | 0.54 | 0.32 | 0.57 | 0.9041 | 3.758 |
| Cycloheptanol | 1.83 | 0.51 | 0.54 | 0.32 | 0.58 | 1.0450 | 4.407 |
| Prop-2-en-1-ol | 0.17 | 0.34 | 0.44 | 0.44 | 0.47 | 0.5470 | 1.951 |
| 2-Methoxyethanol | -0.77 | 0.27 | 0.50 | 0.30 | 0.84 | 0.6487 | 2.490 |
| 2-Ethoxyethanol | -0.10 | 0.24 | 0.50 | 0.30 | 0.83 | 0.7896 | 2.815 |
| 2,2,2-Trifluoroethanol | 0.41 | 0.02 | 0.60 | 0.57 | 0.25 | 0.5622 | 1.224 |
| 1,1,1,3,3,3-Hexafluoro-2-propanol | 1.66 | -0.24 | 0.55 | 0.77 | 0.10 | 0.8162 | 1.392 |
| Ethanethiol | 1.18 | 0.39 | 0.35 | 0.00 | 0.24 | 0.5539 | 2.173 |
| n-Propanethiol | 1.81 | 0.39 | 0.35 | 0.00 | 0.24 | 0.6948 | 2.685 |
| n-Butanethiol | 2.28 | 0.38 | 0.35 | 0.00 | 0.24 | 0.8357 | 3.111 |
| Diethyl sulfide | 1.95 | 0.37 | 0.38 | 0.00 | 0.32 | 0.8357 | 3.104 |
| Di-n-propyl sulfide | 2.96 | 0.36 | 0.38 | 0.00 | 0.32 | 1.1175 | 4.120 |
| Diethyl disulfide | 2.80 | 0.67 | 0.48 | 0.00 | 0.29 | 0.9992 | 4.210 |
| Sulfur hexafluoride | 1.68 | -0.60 | -0.20 | 0.00 | 0.00 | 0.5843 | -0.120 |
| Benzene | 2.13 | 0.61 | 0.52 | 0.00 | 0.14 | 0.7164 | 2.786 |
| Toluene | 2.73 | 0.60 | 0.52 | 0.00 | 0.14 | 0.8573 | 3.325 |
| Ethylbenzene | 3.15 | 0.61 | 0.51 | 0.00 | 0.15 | 0.9982 | 3.778 |
| o-Xylene | 3.12 | 0.66 | 0.56 | 0.00 | 0.16 | 0.9982 | 3.939 |
| m-Xylene | 3.20 | 0.62 | 0.52 | 0.00 | 0.16 | 0.9982 | 3.839 |
| p-Xylene | 3.15 | 0.61 | 0.52 | 0.00 | 0.16 | 0.9982 | 3.839 |
| n-Propylbenzene | 3.72 | 0.60 | 0.50 | 0.00 | 0.15 | 1.1391 | 4.230 |
| Isopropylbenzene | 3.66 | 0.60 | 0.49 | 0.00 | 0.16 | 1.1391 | 4.084 |
| 1,2,3-Trimethylbenzene | 3.66 | 0.73 | 0.61 | 0.00 | 0.19 | 1.1391 | 4.565 |
| 1,2,4-Trimethylbenzene | 3.56 | 0.68 | 0.56 | 0.00 | 0.19 | 1.1391 | 4.441 |
| 1,3,5-Trimethylbenzene | 3.59 | 0.65 | 0.52 | 0.00 | 0.19 | 1.1391 | 4.344 |
| 2-Ethyltoluene | 3.53 | 0.68 | 0.55 | 0.00 | 0.18 | 1.1391 | 4.346 |
| 4-Ethyltoluene | 3.63 | 0.63 | 0.51 | 0.00 | 0.18 | 1.1391 | 4.289 |
| n-Butylbenzene | 4.38 | 0.60 | 0.51 | 0.00 | 0.15 | 1.2800 | 4.730 |
| tert-Butylbenzene | 4.11 | 0.62 | 0.49 | 0.00 | 0.18 | 1.2800 | 4.413 |
| 4-Isopropyltoluene | 4.10 | 0.61 | 0.49 | 0.00 | 0.19 | 1.2800 | 4.590 |
| n-Pentylbenzene | 4.90 | 0.59 | 0.51 | 0.00 | 0.15 | 1.4209 | 5.230 |
| n-Hexylbenzene | 5.52 | 0.59 | 0.50 | 0.00 | 0.15 | 1.5618 | 5.720 |
| Styrene | 2.95 | 0.85 | 0.65 | 0.00 | 0.16 | 0.9552 | 3.856 |
| a-Methylstyrene | 3.35 | 0.85 | 0.64 | 0.00 | 0.19 | 1.0961 | 4.292 |
| Biphenyl | 4.06 | 1.36 | 0.99 | 0.00 | 0.26 | 1.3242 | 6.014 |
| Naphthalene | 3.30 | 1.34 | 0.92 | 0.00 | 0.20 | 1.0854 | 5.161 |
| 1-Methylnaphthalene | 3.87 | 1.34 | 0.94 | 0.00 | 0.22 | 1.2263 | 5.802 |
| 1,3-Dimethylnaphthalene | 4.42 | 1.39 | 0.92 | 0.00 | 0.20 | 1.3672 | 6.236 |

| | | | | | | | |
|---|---|---|---|---|---|---|---|
| 1,4-Dimethylnaphthalene | 4.42 | 1.40 | 0.91 | 0.00 | 0.20 | 1.3672 | 6.339 |
| 2,3-Dimethylnaphthalene | 4.40 | 1.43 | 0.95 | 0.00 | 0.20 | 1.3672 | 6.291 |
| 2,6-Dimethylnaphthalene | 4.31 | 1.33 | 0.91 | 0.00 | 0.20 | 1.3672 | 6.226 |
| 1-Ethylnaphthalene | 4.39 | 1.37 | 0.87 | 0.00 | 0.20 | 1.3672 | 6.136 |
| 2,3-Dihydro-1H-indene | 3.18 | 0.83 | 0.62 | 0.00 | 0.17 | 1.0305 | 4.590 |
| Acenaphthene | 3.92 | 1.60 | 1.05 | 0.00 | 0.22 | 1.2586 | 6.469 |
| Fluorene | 4.18 | 1.59 | 1.06 | 0.00 | 0.25 | 1.3565 | 6.922 |
| Anthracene | 4.45 | 2.29 | 1.34 | 0.00 | 0.28 | 1.4544 | 7.568 |
| Phenanthrene | 4.46 | 2.06 | 1.29 | 0.00 | 0.29 | 1.4544 | 7.632 |
| Pyrene | 5.18 | 2.81 | 1.71 | 0.00 | 0.28 | 1.5846 | 8.833 |
| Fluorobenzene | 2.27 | 0.48 | 0.57 | 0.00 | 0.10 | 0.7541 | 2.788 |
| Benzotrifluoride | 3.01 | 0.23 | 0.48 | 0.00 | 0.10 | 0.9704 | 2.894 |
| Chlorobenzene | 2.89 | 0.72 | 0.65 | 0.00 | 0.07 | 0.8388 | 3.657 |
| 1,2-Dichlorobenzene | 3.43 | 0.87 | 0.78 | 0.00 | 0.04 | 0.9612 | 4.518 |
| 1,3-Dichlorobenzene | 3.53 | 0.85 | 0.73 | 0.00 | 0.02 | 0.9612 | 4.410 |
| 1,4-Dichlorobenzene | 3.44 | 0.83 | 0.75 | 0.00 | 0.02 | 0.9612 | 4.435 |
| 1,2,3-Trichlorobenzene | 4.05 | 1.03 | 0.86 | 0.00 | 0.00 | 1.0836 | 5.419 |
| 1,2,4-Trichlorobenzene | 4.02 | 0.98 | 0.81 | 0.00 | 0.00 | 1.0836 | 5.248 |
| 1,3,5-Trichlorobenzene | 4.19 | 0.98 | 0.73 | 0.00 | 0.00 | 1.0836 | 5.045 |
| 1,2,3,4-Tetrachlorobenzene | 4.64 | 1.18 | 0.92 | 0.00 | 0.00 | 1.2060 | 6.171 |
| 1,2,3,5-Tetrachlorobenzene | 4.65 | 1.16 | 0.85 | 0.00 | 0.00 | 1.2060 | 5.922 |
| 1,2,4,5-Tetrachlorobenzene | 4.60 | 1.16 | 0.86 | 0.00 | 0.00 | 1.2060 | 5.926 |
| 2-Chlorotoluene | 3.42 | 0.76 | 0.65 | 0.00 | 0.07 | 0.9797 | 4.173 |
| Bromobenzene | 2.99 | 0.88 | 0.73 | 0.00 | 0.09 | 0.8914 | 4.041 |
| 4-Bromotoluene | 3.50 | 0.88 | 0.74 | 0.00 | 0.09 | 1.0323 | 4.586 |
| Iodobenzene | 3.25 | 1.19 | 0.82 | 0.00 | 0.12 | 0.9746 | 4.502 |
| Methyl phenyl ether | 2.11 | 0.71 | 0.75 | 0.00 | 0.29 | 0.9160 | 3.890 |
| Ethyl phenyl ether | 2.51 | 0.68 | 0.70 | 0.00 | 0.32 | 1.0569 | 4.242 |
| Benzaldehyde | 1.48 | 0.82 | 1.00 | 0.00 | 0.39 | 0.8730 | 4.008 |
| 4-Methylbenzaldehyde | 2.09 | 0.86 | 1.00 | 0.00 | 0.47 | 1.0139 | 4.592 |
| Acetophenone | 1.58 | 0.82 | 1.01 | 0.00 | 0.48 | 1.0139 | 4.501 |
| Methyl benzoate | 2.12 | 0.73 | 0.85 | 0.00 | 0.46 | 1.0726 | 4.704 |
| Ethyl benzoate | 2.64 | 0.69 | 0.85 | 0.00 | 0.46 | 1.2135 | 5.075 |
| Benzonitrile | 1.56 | 0.74 | 1.11 | 0.00 | 0.33 | 0.8711 | 4.039 |
| o-Toluidine | 1.32 | 0.97 | 0.92 | 0.23 | 0.45 | 0.9571 | 4.442 |
| 2,6-Dimethylaniline | 1.84 | 0.97 | 0.89 | 0.20 | 0.46 | 1.0980 | 5.028 |
| 2-Chloroaniline | 1.91 | 1.03 | 0.92 | 0.25 | 0.31 | 0.9386 | 4.674 |
| 3-Chloroaniline | 1.99 | 1.05 | 1.10 | 0.30 | 0.30 | 0.9386 | 4.909 |
| 4-Chloroaniline | 1.83 | 1.06 | 1.13 | 0.30 | 0.31 | 0.9386 | 4.889 |
| 2-Nitroaniline | 1.85 | 1.18 | 1.37 | 0.30 | 0.36 | 0.9904 | 5.627 |
| 3-Nitroaniline | 1.37 | 1.20 | 1.71 | 0.40 | 0.35 | 0.9904 | 5.880 |
| 4-Nitroaniline | 1.39 | 1.22 | 1.92 | 0.46 | 0.35 | 0.9904 | 6.042 |
| 1-Naphthylamine | 2.25 | 1.67 | 1.20 | 0.20 | 0.57 | 1.1852 | 6.490 |
| 2-Naphthylamine | 2.28 | 1.67 | 1.28 | 0.22 | 0.55 | 1.1852 | 6.540 |
| N,N-Dimethylaniline | 1.98 | 0.96 | 0.84 | 0.00 | 0.41 | 1.0980 | 4.701 |
| Nitrobenzene | 1.85 | 0.87 | 1.11 | 0.00 | 0.28 | 0.8906 | 4.557 |
| 2-Nitrotoluene | 2.30 | 0.87 | 1.11 | 0.00 | 0.28 | 1.0315 | 4.878 |
| 3-Nitrotoluene | 2.42 | 0.87 | 1.10 | 0.00 | 0.25 | 1.0315 | 5.097 |
| Benzamide | 0.64 | 0.99 | 1.50 | 0.49 | 0.67 | 0.9728 | 5.182 |
| Phenol | 1.46 | 0.81 | 0.89 | 0.60 | 0.30 | 0.7751 | 3.766 |
| o-Cresol | 1.98 | 0.84 | 0.86 | 0.52 | 0.30 | 0.9160 | 4.218 |
| p-Cresol | 1.97 | 0.82 | 0.87 | 0.57 | 0.31 | 0.9160 | 4.312 |
| 2,4-Dimethylphenol | 2.30 | 0.84 | 0.79 | 0.52 | 0.40 | 1.0569 | 4.770 |
| 2,5-Dimethylphenol | 2.33 | 0.84 | 0.79 | 0.54 | 0.37 | 1.0569 | 4.774 |
| 2,6-Dimethylphenol | 2.36 | 0.84 | 0.79 | 0.39 | 0.38 | 1.0569 | 4.680 |
| 3,4-Dimethylphenol | 2.23 | 0.83 | 0.90 | 0.55 | 0.38 | 1.0569 | 4.980 |
| 3,5-Dimethylphenol | 2.35 | 0.82 | 0.84 | 0.57 | 0.36 | 1.0569 | 4.856 |

| | | | | | | | |
|---|---|---|---|---|---|---|---|
| 3-Ethylphenol | 2.40 | 0.81 | 0.91 | 0.55 | 0.37 | 1.0569 | 4.741 |
| 4-Ethylphenol | 2.58 | 0.80 | 0.90 | 0.55 | 0.36 | 1.0569 | 4.737 |
| 4-tert-Butylphenol | 3.31 | 0.81 | 0.91 | 0.56 | 0.40 | 1.3387 | 5.264 |
| 2-Fluorophenol | 1.71 | 0.66 | 0.69 | 0.61 | 0.26 | 0.8128 | 3.453 |
| 4-Fluorophenol | 1.77 | 0.67 | 0.97 | 0.63 | 0.23 | 0.8128 | 3.844 |
| 2-Chlorophenol | 2.15 | 0.85 | 0.88 | 0.32 | 0.31 | 0.8975 | 4.178 |
| 3-Chlorophenol | 2.50 | 0.91 | 1.06 | 0.69 | 0.15 | 0.8975 | 4.773 |
| 4-Chlorophenol | 2.40 | 0.92 | 1.08 | 0.67 | 0.20 | 0.8975 | 4.775 |
| 4-Bromophenol | 2.59 | 1.08 | 1.17 | 0.67 | 0.20 | 0.9501 | 5.135 |
| 2-Iodophenol | 2.65 | 1.36 | 1.00 | 0.40 | 0.35 | 1.0333 | 4.964 |
| 2-Methoxyphenol | 1.32 | 0.84 | 0.91 | 0.22 | 0.52 | 0.9747 | 4.449 |
| 3-Methoxyphenol | 1.58 | 0.88 | 1.17 | 0.59 | 0.39 | 0.9747 | 4.803 |
| 3-Cyanophenol | 1.70 | 0.93 | 1.55 | 0.84 | 0.25 | 0.9298 | 5.181 |
| 4-Cyanophenol | 1.60 | 0.94 | 1.63 | 0.80 | 0.29 | 0.9298 | 5.420 |
| 2-Nitrophenol | 1.85 | 1.02 | 1.05 | 0.05 | 0.37 | 0.9493 | 4.760 |
| 3-Nitrophenol | 2.00 | 1.05 | 1.57 | 0.79 | 0.23 | 0.9493 | 5.692 |
| 4-Nitrophenol | 1.91 | 1.07 | 1.72 | 0.82 | 0.26 | 0.9493 | 5.876 |
| 1-Naphthol | 2.84 | 1.52 | 1.10 | 0.66 | 0.34 | 1.1441 | 6.284 |
| 2-Naphthol | 2.70 | 1.52 | 1.08 | 0.61 | 0.40 | 1.1441 | 6.200 |
| Benzyl alcohol | 1.10 | 0.80 | 0.87 | 0.33 | 0.56 | 0.9160 | 4.221 |
| 2-Phenylethanol | 1.36 | 0.81 | 0.86 | 0.31 | 0.65 | 1.0569 | 4.628 |
| 3-Phenylpropan-1-ol | 1.88 | 0.82 | 0.90 | 0.30 | 0.67 | 1.1978 | 5.180 |
| Thiophenol | 2.52 | 1.00 | 0.80 | 0.09 | 0.16 | 0.8799 | 4.110 |
| Phenylmethylsulphide | 2.47 | 1.07 | 0.92 | 0.00 | 0.26 | 1.0208 | 4.659 |
| Pyridine | 0.65 | 0.63 | 0.84 | 0.00 | 0.52 | 0.6753 | 3.022 |
| 3-Methylpyridine | 1.20 | 0.63 | 0.81 | 0.00 | 0.54 | 0.8162 | 3.631 |
| 3-Ethylpyridine | 1.66 | 0.64 | 0.79 | 0.00 | 0.57 | 0.9571 | 4.093 |
| 4-Ethylpyridine | 1.65 | 0.63 | 0.80 | 0.00 | 0.57 | 0.9571 | 4.124 |
| 2-Chloropyridine | 1.22 | 0.74 | 1.03 | 0.00 | 0.37 | 0.7977 | 3.875 |
| 3-Chloropyridine | 1.33 | 0.73 | 0.83 | 0.00 | 0.41 | 0.7977 | 3.783 |
| 3-Cyanopyridine | 0.23 | 0.75 | 1.26 | 0.00 | 0.62 | 0.8300 | 4.164 |
| 4-Cyanopyridine | 0.46 | 0.75 | 1.21 | 0.00 | 0.59 | 0.8300 | 4.033 |
| 2-Methylpyrazine | 0.23 | 0.63 | 0.86 | 0.00 | 0.67 | 0.7751 | 3.254 |
| Thiophene | 1.81 | 0.69 | 0.57 | 0.00 | 0.15 | 0.6411 | 2.819 |
| 2-Methylthiophene | 2.33 | 0.69 | 0.56 | 0.00 | 0.16 | 0.7820 | 3.308 |

Table S2. Data and descriptors for log $K_{aw}$.[6]

| | Log $K_{aw}$ | *E* | *S* | *A* | *B* | *V* | *L* |
|---|---|---|---|---|---|---|---|
| Methane | 1.46 | 0.00 | 0.00 | 0.00 | 0.00 | 0.2495 | -0.323 |
| Ethane | 1.34 | 0.00 | 0.00 | 0.00 | 0.00 | 0.3904 | 0.492 |
| Propane | 1.44 | 0.00 | 0.00 | 0.00 | 0.00 | 0.5313 | 1.050 |
| n-Butane | 1.52 | 0.00 | 0.00 | 0.00 | 0.00 | 0.6722 | 1.615 |
| 2-Methylpropane | 1.70 | 0.00 | 0.00 | 0.00 | 0.00 | 0.6722 | 1.409 |
| n-Pentane | 1.70 | 0.00 | 0.00 | 0.00 | 0.00 | 0.8131 | 2.162 |
| 2-Methylbutane | 1.75 | 0.00 | 0.00 | 0.00 | 0.00 | 0.8131 | 2.013 |
| 2,2-Dimethylpropane | 1.84 | 0.00 | 0.00 | 0.00 | 0.00 | 0.8131 | 1.820 |
| n-Hexane | 1.82 | 0.00 | 0.00 | 0.00 | 0.00 | 0.9540 | 2.668 |
| 2-Methylpentane | 1.84 | 0.00 | 0.00 | 0.00 | 0.00 | 0.9540 | 2.503 |
| 3-Methylpentane | 1.84 | 0.00 | 0.00 | 0.00 | 0.00 | 0.9540 | 2.581 |
| 2,2-Dimethylbutane | 1.84 | 0.00 | 0.00 | 0.00 | 0.00 | 0.9540 | 2.352 |
| 2,3-Dimethylbutane | 1.72 | 0.00 | 0.00 | 0.00 | 0.00 | 0.9540 | 2.495 |
| n-Heptane | 1.96 | 0.00 | 0.00 | 0.00 | 0.00 | 1.0949 | 3.173 |
| 2-Methylhexane | 2.15 | 0.00 | 0.00 | 0.00 | 0.00 | 1.0949 | 3.001 |
| 3-Methylhexane | 1.99 | 0.00 | 0.00 | 0.00 | 0.00 | 1.0949 | 3.044 |
| 2,2-Dimethylpentane | 2.11 | 0.00 | 0.00 | 0.00 | 0.00 | 1.0949 | 2.796 |

| | | | | | | | |
|---|---|---|---|---|---|---|---|
| 2,3-Dimethylpentane | 1.85 | 0.00 | 0.00 | 0.00 | 0.00 | 1.0949 | 3.016 |
| 2,4-Dimethylpentane | 2.08 | 0.00 | 0.00 | 0.00 | 0.00 | 1.0949 | 2.809 |
| 3,3-Dimethylpentane | 1.88 | 0.00 | 0.00 | 0.00 | 0.00 | 1.0949 | 2.946 |
| n-Octane | 2.11 | 0.00 | 0.00 | 0.00 | 0.00 | 1.2358 | 3.677 |
| 3-Methylheptane | 2.18 | 0.00 | 0.00 | 0.00 | 0.00 | 1.2358 | 3.510 |
| 2,2,4-Trimethylpentane | 2.12 | 0.00 | 0.00 | 0.00 | 0.00 | 1.2358 | 3.106 |
| 2,3,4-Trimethylpentane | 1.88 | 0.00 | 0.00 | 0.00 | 0.00 | 1.2358 | 3.481 |
| n-Nonane | 2.30 | 0.00 | 0.00 | 0.00 | 0.00 | 1.3767 | 4.182 |
| 2,2,5-Trimethylhexane | 2.15 | 0.00 | 0.00 | 0.00 | 0.00 | 1.3767 | 3.567 |
| n-Decane | 2.32 | 0.00 | 0.00 | 0.00 | 0.00 | 1.5176 | 4.686 |
| Cyclopropane | 0.55 | 0.18 | 0.15 | 0.00 | 0.00 | 0.4227 | 1.314 |
| Cyclopentane | 0.88 | 0.26 | 0.10 | 0.00 | 0.00 | 0.7045 | 2.477 |
| Methylcyclopentane | 1.17 | 0.23 | 0.10 | 0.00 | 0.00 | 0.8454 | 2.816 |
| n-Propylcyclopentane | 1.56 | 0.23 | 0.10 | 0.00 | 0.00 | 1.1272 | 3.803 |
| Cyclohexane | 0.90 | 0.31 | 0.10 | 0.00 | 0.00 | 0.8454 | 2.964 |
| Methylcyclohexane | 1.25 | 0.24 | 0.10 | 0.00 | 0.00 | 0.9863 | 3.323 |
| cis- 1,2-Dimethylcyclohexane | 1.16 | 0.28 | 0.10 | 0.00 | 0.00 | 1.1272 | 3.795 |
| trans- 1,4-Dimethylcvclohexane | 1.55 | 0.19 | 0.10 | 0.00 | 0.00 | 1.1272 | 3.538 |
| Ethene | 0.94 | 0.11 | 0.10 | 0.00 | 0.07 | 0.3474 | 0.289 |
| Propene | 0.97 | 0.10 | 0.08 | 0.00 | 0.07 | 0.4883 | 0.946 |
| But- 1 -ene | 1.01 | 0.10 | 0.08 | 0.00 | 0.07 | 0.6292 | 1.491 |
| Pent-1-ene | 1.23 | 0.09 | 0.08 | 0.00 | 0.07 | 0.7701 | 2.047 |
| (z)-Pent-2-ene | 0.96 | 0.14 | 0.08 | 0.00 | 0.07 | 0.7701 | 2.211 |
| 3-Methylbut-1-ene | 1.34 | 0.06 | 0.08 | 0.00 | 0.07 | 0.7701 | 1.910 |
| 2-Methylbut-2-ene | 0.96 | 0.16 | 0.08 | 0.00 | 0.07 | 0.7701 | 2.226 |
| Hex-l-ene | 1.16 | 0.08 | 0.08 | 0.00 | 0.07 | 0.9110 | 2.572 |
| 2-Methylpent-l-ene | 1.08 | 0.09 | 0.08 | 0.00 | 0.07 | 0.9110 | 2.588 |
| Hept-1-ene | 1.22 | 0.09 | 0.08 | 0.00 | 0.07 | 1.0519 | 3.063 |
| Oct-1-ene | 1.41 | 0.09 | 0.08 | 0.00 | 0.07 | 1.1928 | 3.568 |
| Non-l-ene | 1.51 | 0.00 | 0.08 | 0.00 | 0.07 | 1.3337 | 4.073 |
| Buta-1,3-diene | 0.45 | 0.32 | 0.23 | 0.00 | 0.10 | 0.5862 | 1.543 |
| 2-Methylbuta- 1,3-diene | 0.50 | 0.31 | 0.23 | 0.00 | 0.10 | 0.7271 | 2.101 |
| 2,3-Dimethylbuta- 1,3-diene | 0.29 | 0.35 | 0.23 | 0.00 | 0.14 | 0.8680 | 2.690 |
| Cyclopentene | 0.41 | 0.34 | 0.20 | 0.00 | 0.10 | 0.6615 | 2.402 |
| Cyclohexene | 0.27 | 0.40 | 0.20 | 0.00 | 0.10 | 0.8024 | 3.021 |
| 1-Methylcyclohexene | 0.49 | 0.39 | 0.20 | 0.00 | 0.10 | 0.9433 | 3.483 |
| Cyclohepta-1 3,5-triene | -0.73 | 0.76 | 0.46 | 0.00 | 0.18 | 0.8573 | 3.442 |
| Propyne | -0.35 | 0.19 | 0.25 | 0.13 | 0.15 | 0.4453 | 1.025 |
| But-1-yne | -0.12 | 0.18 | 0.23 | 0.13 | 0.15 | 0.5862 | 1.520 |
| Pent-1-yne | 0.01 | 0.17 | 0.23 | 0.13 | 0.10 | 0.7271 | 2.010 |
| Hex-1-yne | 0.21 | 0.17 | 0.23 | 0.13 | 0.10 | 0.8680 | 2.510 |
| Hept-1-yne | 0.44 | 0.16 | 0.23 | 0.13 | 0.10 | 1.0089 | 3.000 |
| Oct-1-yne | 0.52 | 0.16 | 0.23 | 0.13 | 0.10 | 1.1498 | 3.521 |
| Tetrafluoromethane | 2.29 | -0.28 | -0.20 | 0.00 | 0.00 | 0.3203 | -0.800 |
| Chloromethane | -0.40 | 0.25 | 0.43 | 0.00 | 0.08 | 0.3719 | 1.163 |
| Dichloromethane | -0.96 | 0.39 | 0.57 | 0.10 | 0.05 | 0.4943 | 2.019 |
| Trichloromethane | -0.79 | 0.43 | 0.49 | 0.15 | 0.02 | 0.6167 | 2.480 |
| Tetrachloromethane | 0.06 | 0.46 | 0.38 | 0.00 | 0.00 | 0.7391 | 2.823 |
| Chloroethane | -0.46 | 0.23 | 0.40 | 0.00 | 0.10 | 0.5128 | 1.678 |
| 1,1-Dichloroethane | -0.62 | 0.32 | 0.49 | 0.10 | 0.10 | 0.6352 | 2.316 |
| 1,2-Dichloroethane | -1.31 | 0.42 | 0.64 | 0.10 | 0.11 | 0.6352 | 2.573 |
| 1, 1, 1 -Trichloroethane | -0.14 | 0.37 | 0.41 | 0.00 | 0.09 | 0.7576 | 2.733 |
| 1, 1,2-Trichloroethane | -1.46 | 0.50 | 0.68 | 0.13 | 0.08 | 0.7576 | 3.290 |
| 1, 1,2,2-Tetrachloroethane | -1.81 | 0.60 | 0.76 | 0.16 | 0.12 | 0.8800 | 3.803 |
| 1, 1, 1,2-Tetrachloroethane | -0.94 | 0.54 | 0.63 | 0.10 | 0.08 | 0.8800 | 3.641 |
| Pentachloroethane | -1.02 | 0.65 | 0.66 | 0.17 | 0.06 | 1.0024 | 4.267 |
| 1-Chloropropane | -0.24 | 0.22 | 0.40 | 0.00 | 0.10 | 0.6537 | 2.202 |

| | | | | | | | |
|---|---|---|---|---|---|---|---|
| 2-Chloropropane | -0.18 | 0.18 | 0.35 | 0.00 | 0.12 | 0.6537 | 1.970 |
| 1,2-Dichloropropane | -0.93 | 0.37 | 0.60 | 0.10 | 0.11 | 0.7761 | 2.857 |
| 1,3-Dichloropropane | -1.39 | 0.41 | 0.74 | 0.00 | 0.17 | 0.7761 | 3.101 |
| 1-Chlorobutane | -0.12 | 0.21 | 0.40 | 0.00 | 0.10 | 0.7946 | 2.722 |
| 2-Chlorobutane | 0.00 | 0.19 | 0.35 | 0.00 | 0.12 | 0.7946 | 2.540 |
| 2-Chloro-2-methylpropane | 0.80 | 0.14 | 0.25 | 0.00 | 0.12 | 0.7946 | 2.217 |
| 1-Chloropentane | -0.05 | 0.21 | 0.40 | 0.00 | 0.10 | 0.9355 | 3.223 |
| 1-Chlorohexane | 0.00 | 0.20 | 0.40 | 0.00 | 0.10 | 1.0764 | 3.777 |
| 1-Chloroheptane | 0.21 | 0.19 | 0.40 | 0.00 | 0.10 | 1.2173 | 4.282 |
| 1,1-Dichloroethene | 0.18 | 0.36 | 0.34 | 0.00 | 0.05 | 0.5922 | 2.110 |
| (Z)- 1,2-Dichloroethene | -0.86 | 0.44 | 0.61 | 0.11 | 0.05 | 0.5922 | 2.439 |
| (E)- 1,2,-Dichloroethene | -0.57 | 0.43 | 0.41 | 0.09 | 0.05 | 0.5922 | 2.278 |
| Trichloroethene | -0.32 | 0.52 | 0.40 | 0.08 | 0.03 | 0.7146 | 2.997 |
| Tetrachloroethene | 0.07 | 0.64 | 0.42 | 0.00 | 0.00 | 0.8370 | 3.584 |
| 1-Chloroprop-2-ene | -0.42 | 0.33 | 0.56 | 0.00 | 0.05 | 0.6106 | 2.109 |
| Bromomethane | -0.60 | 0.40 | 0.43 | 0.00 | 0.10 | 0.4245 | 1.630 |
| Dibromomethane | -1.44 | 0.71 | 0.67 | 0.10 | 0.10 | 0.5995 | 2.886 |
| Tribromomethane | -1.56 | 0.97 | 0.68 | 0.15 | 0.09 | 0.7745 | 3.784 |
| Bromoethane | -0.54 | 0.37 | 0.40 | 0.00 | 0.12 | 0.5654 | 2.120 |
| 1-Bromopropane | -0.41 | 0.37 | 0.40 | 0.00 | 0.12 | 0.7063 | 2.620 |
| 2-Bromopropane | -0.35 | 0.33 | 0.35 | 0.00 | 0.14 | 0.7063 | 2.390 |
| 1-Bromobutane | -0.29 | 0.36 | 0.40 | 0.00 | 0.12 | 0.8472 | 3.105 |
| 1 -Bromo-2-methylpropane | -0.02 | 0.34 | 0.37 | 0.00 | 0.12 | 0.8472 | 2.960 |
| 2-Bromo-2-methylpropane | 0.62 | 0.31 | 0.25 | 0.00 | 0.14 | 0.8472 | 2.616 |
| 1-Bromopentane | -0.07 | 0.36 | 0.40 | 0.00 | 0.12 | 0.9881 | 3.611 |
| 1-Bromohexane | 0.13 | 0.35 | 0.40 | 0.00 | 0.12 | 1.1290 | 4.130 |
| 1-Bromoheptane | 0.25 | 0.34 | 0.40 | 0.00 | 0.12 | 1.2699 | 4.663 |
| 1-Bromooctane | 0.38 | 0.34 | 0.40 | 0.00 | 0.12 | 1.4108 | 5.000 |
| Iodomethane | -0.65 | 0.68 | 0.43 | 0.00 | 0.13 | 0.5077 | 2.106 |
| Iodoethane | -0.54 | 0.64 | 0.40 | 0.00 | 0.15 | 0.6486 | 2.573 |
| 1-Iodopropane | -0.39 | 0.63 | 0.40 | 0.00 | 0.15 | 0.7895 | 3.130 |
| 1-Iodobutane | -0.18 | 0.63 | 0.40 | 0.00 | 0.15 | 0.9304 | 3.628 |
| 1-Iodopentane | -0.10 | 0.62 | 0.40 | 0.00 | 0.15 | 1.0713 | 4.130 |
| 1-Iodohexane | 0.06 | 0.62 | 0.40 | 0.00 | 0.15 | 1.2122 | 4.620 |
| Halothane | -0.08 | 0.10 | 0.38 | 0.15 | 0.03 | 0.7410 | 2.177 |
| Teflurane | 0.37 | -0.07 | 0.21 | 0.20 | 0.00 | 0.6360 | 1.370 |
| Diethyl ether | -1.17 | 0.04 | 0.25 | 0.00 | 0.45 | 0.7309 | 2.015 |
| Di-n-propyl ether | -0.85 | 0.01 | 0.25 | 0.00 | 0.45 | 1.0127 | 2.954 |
| Diisopropyl ether | -0.39 | 0.00 | 0.19 | 0.00 | 0.45 | 1.0127 | 2.482 |
| Di-n-butyl ether | -0.61 | 0.00 | 0.25 | 0.00 | 0.45 | 1.2945 | 3.924 |
| Methoxyflurane | -0.82 | 0.11 | 0.67 | 0.17 | 0.05 | 0.8700 | 2.864 |
| Isoflurane | 0.07 | -0.24 | 0.50 | 0.10 | 0.10 | 0.8010 | 1.576 |
| Tetrahydrofuran | -2.55 | 0.29 | 0.52 | 0.00 | 0.48 | 0.6223 | 2.636 |
| 2-Methylletrahydrofuran | -2.42 | 0.24 | 0.48 | 0.00 | 0.53 | 0.7632 | 2.820 |
| 2,5-Dimethyltetrahydrofuran | -2.14 | 0.20 | 0.38 | 0.00 | 0.58 | 0.9041 | 2.980 |
| Tetrahydropyran | -2.29 | 0.28 | 0.47 | 0.00 | 0.55 | 0.8288 | 3.057 |
| 1,4-Dioxane | -3.71 | 0.33 | 0.75 | 0.00 | 0.64 | 0.6810 | 2.892 |
| Formaldehyde | -2.02 | 0.22 | 0.70 | 0.00 | 0.33 | 0.2652 | 0.730 |
| Acetaldehyde | -2.57 | 0.21 | 0.67 | 0.00 | 0.45 | 0.4061 | 1.230 |
| Propionaldehyde | -2.52 | 0.20 | 0.65 | 0.00 | 0.45 | 0.5470 | 1.815 |
| Butyraldehyde | -2.33 | 0.19 | 0.65 | 0.00 | 0.45 | 0.6879 | 2.270 |
| Isobutyraldehyde | -2.10 | 0.14 | 0.62 | 0.00 | 0.45 | 0.6879 | 2.120 |
| Pentanal | -2.22 | 0.16 | 0.65 | 0.00 | 0.45 | 0.8288 | 2.851 |
| Hexanal | -2.06 | 0.15 | 0.65 | 0.00 | 0.45 | 0.9697 | 3.357 |
| Heptanal | -1.96 | 0.14 | 0.65 | 0.00 | 0.45 | 1.1106 | 3.865 |
| Octanal | -1.68 | 0.16 | 0.65 | 0.00 | 0.45 | 1.2515 | 4.361 |
| Nonanal | -1.52 | 0.15 | 0.65 | 0.00 | 0.45 | 1.3924 | 4.856 |

| | | | | | | | |
|---|---|---|---|---|---|---|---|
| (E)-But-2-enal | -3.10 | 0.39 | 0.80 | 0.00 | 0.49 | 0.6449 | 2.570 |
| (E)-Hex-2-enal | -2.70 | 0.40 | 0.80 | 0.00 | 0.45 | 0.7860 | 3.400 |
| Propanone | -2.79 | 0.18 | 0.70 | 0.04 | 0.51 | 0.5470 | 1.696 |
| Butanone | -2.72 | 0.17 | 0.70 | 0.00 | 0.51 | 0.6879 | 2.287 |
| Pentan-2-one | -2.58 | 0.14 | 0.68 | 0.00 | 0.51 | 0.8288 | 2.755 |
| Pentan-3-one | -2.50 | 0.15 | 0.66 | 0.00 | 0.51 | 0.8288 | 2.811 |
| 3-Methylbutan-2-one | -2.38 | 0.13 | 0.65 | 0.00 | 0.51 | 0.8288 | 2.692 |
| Hexan-2-one | -2.41 | 0.14 | 0.68 | 0.00 | 0.51 | 0.9676 | 3.262 |
| 4-Methylpentan-2-one | -2.24 | 0.11 | 0.65 | 0.00 | 0.51 | 0.9676 | 3.089 |
| Heptan-2-one | -2.23 | 0.12 | 0.68 | 0.00 | 0.51 | 1.1106 | 3.760 |
| Heptan-4-one | -2.14 | 0.11 | 0.66 | 0.00 | 0.51 | 1.1106 | 3.705 |
| Octan-2-one | -2.11 | 0.11 | 0.68 | 0.00 | 0.51 | 1.2515 | 4.257 |
| Nonan-2-one | -1.83 | 0.12 | 0.68 | 0.00 | 0.51 | 1.3924 | 4.735 |
| Nonan-5-one | -1.94 | 0.10 | 0.66 | 0.00 | 0.51 | 1.3924 | 4.698 |
| Decan-2-one | -1.72 | 0.11 | 0.68 | 0.00 | 0.51 | 1.5333 | 5.245 |
| Undecan-2-one | -1.58 | 0.10 | 0.68 | 0.00 | 0.51 | 1.6742 | 5.732 |
| Cyclopentanone | -3.45 | 0.37 | 0.86 | 0.00 | 0.52 | 0.7202 | 3.221 |
| Cyclohexanone | -3.60 | 0.40 | 0.86 | 0.00 | 0.56 | 0.8611 | 3.792 |
| Methyl formate | -2.04 | 0.19 | 0.68 | 0.00 | 0.38 | 0.4648 | 1.285 |
| Ethyl formate | -1.88 | 0.15 | 0.66 | 0.00 | 0.38 | 0.6057 | 1.845 |
| n-Propyl formate | -1.82 | 0.13 | 0.63 | 0.00 | 0.38 | 0.7466 | 2.433 |
| Isopropyl formate | -1.48 | 0.09 | 0.60 | 0.00 | 0.40 | 0.7466 | 2.230 |
| isobutyl formate | -1.63 | 0.10 | 0.60 | 0.00 | 0.40 | 0.8875 | 2.789 |
| Isoamyl formate | -1.56 | 0.09 | 0.60 | 0.00 | 0.40 | 1.0284 | 3.306 |
| Methyl acetate | -2.30 | 0.14 | 0.64 | 0.00 | 0.45 | 0.6057 | 1.911 |
| Ethyl acetate | -2.16 | 0.11 | 0.62 | 0.00 | 0.45 | 0.7466 | 2.314 |
| n-Propyl acetate | -2.05 | 0.09 | 0.60 | 0.00 | 0.45 | 0.8875 | 2.819 |
| Isopropyl acetate | -1.94 | 0.06 | 0.57 | 0.00 | 0.47 | 0.8875 | 2.546 |
| n-Butyl acetate | -1.94 | 0.07 | 0.60 | 0.00 | 0.45 | 1.0284 | 3.353 |
| Isobutyl acetate | -1.73 | 0.05 | 0.57 | 0.00 | 0.47 | 1.0284 | 3.161 |
| n-Pentyl acetate | -1.84 | 0.07 | 0.60 | 0.00 | 0.45 | 1.1693 | 3.844 |
| Isoamyl acetate | -1.62 | 0.05 | 0.57 | 0.00 | 0.47 | 1.1693 | 3.740 |
| n-Hexyl acetate | -1.66 | 0.06 | 0.60 | 0.00 | 0.45 | 1.3102 | 4.351 |
| Methyl propanoate | -2.15 | 0.13 | 0.60 | 0.00 | 0.45 | 0.7466 | 2.431 |
| Ethyl propanoate | -1.97 | 0.09 | 0.58 | 0.00 | 0.45 | 0.8875 | 2.807 |
| n-Propyl propanoate | -1.79 | 0.07 | 0.56 | 0.00 | 0.45 | 1.0284 | 3.338 |
| n-Pentyl propanoate | -1.55 | 0.05 | 0.56 | 0.00 | 0.45 | 1.3102 | 4.331 |
| Methyl butanoate | -2.08 | 0.11 | 0.60 | 0.00 | 0.45 | 0.8875 | 2.893 |
| Ethyl butanoate | -1.83 | 0.07 | 0.58 | 0.00 | 0.45 | 1.0284 | 3.271 |
| n-Propyl butanoate | -1.67 | 0.05 | 0.56 | 0.00 | 0.45 | 1.1693 | 3.783 |
| Methyl pentanoate | -1.88 | 0.11 | 0.60 | 0.00 | 0.45 | 1.0284 | 3.392 |
| Ethyl pentanoate | -1.83 | 0.05 | 0.58 | 0.00 | 0.45 | 1.1693 | 3.769 |
| Methyl hexanoate | -1.83 | 0.08 | 0.60 | 0.00 | 0.45 | 1.1693 | 3.874 |
| Ethvl hexanoate | -1.64 | 0.04 | 0.58 | 0.00 | 0.45 | 1.3102 | 4.251 |
| Isobutyl isobutanoate | -1.24 | 0.00 | 0.50 | 0.00 | 0.47 | 1.3102 | 3.885 |
| Acetonitrile | -2.85 | 0.24 | 0.90 | 0.04 | 0.33 | 0.4042 | 1.739 |
| Propanonitrile | -2.82 | 0.16 | 0.90 | 0.02 | 0.36 | 0.5451 | 2.082 |
| 1-Cyanopropane | -2.67 | 0.19 | 0.90 | 0.00 | 0.36 | 0.6860 | 2.548 |
| 1-Cyanobutane | -2.58 | 0.18 | 0.90 | 0.00 | 0.36 | 0.8269 | 3.108 |
| Ammonia | -3.15 | 0.14 | 0.35 | 0.14 | 0.62 | 0.2084 | 0.680 |
| Methylamine | -3.34 | 0.25 | 0.35 | 0.16 | 0.58 | 0.3439 | 1.300 |
| Ethylamine | -3.30 | 0.24 | 0.35 | 0.16 | 0.61 | 0.4902 | 1.677 |
| n-Propylamine | -3.22 | 0.23 | 0.35 | 0.16 | 0.61 | 0.6311 | 2.141 |
| n-Butylamine | -3.11 | 0.22 | 0.35 | 0.16 | 0.61 | 0.7720 | 2.618 |
| n-Pentylamine | -3.00 | 0.21 | 0.35 | 0.16 | 0.61 | 0.9129 | 3.139 |
| n-Hexylamine | -2.90 | 0.20 | 0.35 | 0.16 | 0.61 | 1.0538 | 3.655 |
| n-Heptylamine | -2.78 | 0.20 | 0.35 | 0.16 | 0.61 | 1.1947 | 4.166 |

| | | | | | | | |
|---|---|---|---|---|---|---|---|
| n-Octylamine | -2.68 | 0.19 | 0.35 | 0.16 | 0.61 | 1.3356 | 4.520 |
| Cyclohexylamine | -3.37 | 0.33 | 0.56 | 0.16 | 0.58 | 0.9452 | 3.796 |
| Dimethylamine | -3.15 | 0.19 | 0.30 | 0.08 | 0.66 | 0.4902 | 1.600 |
| Diethylamine | -2.99 | 0.15 | 0.30 | 0.08 | 0.68 | 0.7720 | 2.395 |
| Di-n-propylamine | -2.68 | 0.12 | 0.30 | 0.08 | 0.68 | 1.0538 | 3.351 |
| Diisopropylamine | -2.36 | 0.05 | 0.24 | 0.08 | 0.71 | 1.0538 | 2.893 |
| Di-n-butylamine | -2.38 | 0.11 | 0.30 | 0.08 | 0.68 | 1.3356 | 4.349 |
| Trimethylamine | -2.35 | 0.14 | 0.20 | 0.00 | 0.67 | 0.6311 | 1.620 |
| Triethylamine | -2.36 | 0.10 | 0.15 | 0.00 | 0.79 | 1.0538 | 3.040 |
| Nitromethane | -2.95 | 0.31 | 0.95 | 0.06 | 0.32 | 0.4237 | 1.892 |
| Nitroethane | -2.72 | 0.27 | 0.95 | 0.02 | 0.33 | 0.5646 | 2.414 |
| 1-Nitropropane | -2.45 | 0.24 | 0.95 | 0.00 | 0.31 | 0.7055 | 2.894 |
| 2-Nitropropane | -2.30 | 0.22 | 0.92 | 0.00 | 0.32 | 0.7055 | 2.550 |
| 1-Nitrobutane | -2.27 | 0.23 | 0.95 | 0.00 | 0.29 | 0.8464 | 3.415 |
| 1-Nitropentane | -2.07 | 0.21 | 0.95 | 0.00 | 0.29 | 0.9873 | 3.938 |
| N,N-Dimethylformamide | -5.73 | 0.37 | 1.31 | 0.00 | 0.73 | 0.6468 | 3.173 |
| Acetic acid | -4.91 | 0.27 | 0.65 | 0.61 | 0.45 | 0.4648 | 1.750 |
| Propanoic acid | -4.74 | 0.23 | 0.65 | 0.60 | 0.45 | 0.6057 | 2.290 |
| Butanoic acid | -4.66 | 0.21 | 0.62 | 0.60 | 0.45 | 0.7466 | 2.830 |
| Pentanoic aeid | -4.52 | 0.21 | 0.60 | 0.60 | 0.45 | 0.8875 | 3.380 |
| 3-Meihylbutanoic acid | -4.47 | 0.18 | 0.57 | 0.60 | 0.50 | 0.8875 | 3.140 |
| Hexanoic acid | -4.56 | 0.17 | 0.60 | 0.60 | 0.45 | 1.0284 | 3.920 |
| Water | -4.64 | 0.00 | 0.45 | 0.82 | 0.35 | 0.1673 | 0.260 |
| Methanol | -3.74 | 0.28 | 0.44 | 0.43 | 0.47 | 0.3082 | 0.970 |
| Ethanol | -3.67 | 0.25 | 0.42 | 0.37 | 0.48 | 0.4491 | 1.485 |
| Propan-l-ol | -3.56 | 0.24 | 0.42 | 0.37 | 0.48 | 0.5900 | 2.031 |
| Propan-2-ol | -3.48 | 0.21 | 0.36 | 0.33 | 0.56 | 0.5900 | 1.764 |
| Butan-l-ol | -3.46 | 0.22 | 0.42 | 0.37 | 0.48 | 0.7309 | 2.601 |
| 2-Methylpropan-1 -ol | -3.30 | 0.22 | 0.39 | 0.37 | 0.48 | 0.7309 | 2.413 |
| Butan-2-ol | -3.39 | 0.22 | 0.36 | 0.33 | 0.56 | 0.7309 | 2.338 |
| 2-Methylpropan-2-ol | -3.28 | 0.18 | 0.30 | 0.31 | 0.60 | 0.7309 | 1.963 |
| Pentan-1-ol | -3.35 | 0.22 | 0.42 | 0.37 | 0.48 | 0.8718 | 3.106 |
| Pentan-2-ol | -3.22 | 0.20 | 0.36 | 0.33 | 0.56 | 0.8718 | 2.840 |
| Pentan-3-ol | -3.19 | 0.22 | 0.36 | 0.33 | 0.56 | 0.8718 | 2.860 |
| 2-Methylbutan-l-ol | -3.24 | 0.22 | 0.39 | 0.37 | 0.48 | 0.8718 | 3.011 |
| 3-Methylbulan-l-ol | -3.24 | 0.19 | 0.39 | 0.37 | 0.48 | 0.8718 | 3.011 |
| 2-Methylbutan-2-ol | -3.25 | 0.19 | 0.30 | 0.31 | 0.60 | 0.8718 | 2.630 |
| Hexan-l-ol | -3.23 | 0.21 | 0.42 | 0.37 | 0.48 | 1.0127 | 3.610 |
| Hexan-3-ol | -2.98 | 0.20 | 0.36 | 0.33 | 0.56 | 1.0127 | 3.343 |
| 2-Methylpentan-2-ol | -2.88 | 0.17 | 0.30 | 0.31 | 0.60 | 1.0127 | 3.081 |
| 4-Methylpentan-2-ol | -2.74 | 0.17 | 0.33 | 0.33 | 0.56 | 1.0127 | 3.179 |
| 2-Methylpentan-3-ol | -2.85 | 0.21 | 0.33 | 0.33 | 0.56 | 1.0127 | 3.240 |
| Heptan-l-ol | -3.09 | 0.21 | 0.42 | 0.37 | 0.48 | 1.1536 | 4.115 |
| Octan- 1 -ol | -3.00 | 0.20 | 0.42 | 0.37 | 0.48 | 1.2950 | 4.619 |
| Nonan-1-ol | -2.85 | 0.19 | 0.42 | 0.37 | 0.48 | 1.4354 | 5.124 |
| Decan- 1 -ol | -2.67 | 0.19 | 0.42 | 0.37 | 0.48 | 1.5763 | 5.628 |
| Cyclopentanol | -4.03 | 0.43 | 0.54 | 0.32 | 0.56 | 0.7630 | 3.241 |
| Cyclohexanol | -4.01 | 0.46 | 0.54 | 0.32 | 0.57 | 0.9040 | 3.758 |
| Cycloheptanol | -4.02 | 0.51 | 0.54 | 0.32 | 0.58 | 1.0450 | 4.407 |
| Prop-2-en-1-ol | -3.69 | 0.34 | 0.44 | 0.44 | 0.47 | 0.5470 | 1.951 |
| 2-Methoxyethanol | -4.96 | 0.27 | 0.50 | 0.30 | 0.84 | 0.6487 | 2.490 |
| 2-Ethoxyethanol | -4.91 | 0.24 | 0.50 | 0.30 | 0.83 | 0.7900 | 2.815 |
| 2-Butoxyethanol | -4.59 | 0.20 | 0.50 | 0.30 | 0.83 | 1.0720 | 3.806 |
| 2,2,2-Trifluoroethanol | -3.16 | 0.02 | 0.60 | 0.57 | 0.25 | 0.5022 | 1.224 |
| Hexafluoropropanol | -2.76 | -0.24 | 0.55 | 0.77 | 0.10 | 0.6962 | 1.392 |
| Ethanethiol | -0.84 | 0.39 | 0.35 | 0.00 | 0.24 | 0.5539 | 2.173 |
| n-Propanethiol | -0.78 | 0.39 | 0.35 | 0.00 | 0.24 | 0.6948 | 2.685 |

| | | | | | | | |
|---|---|---|---|---|---|---|---|
| n-Butanethiol | -0.73 | 0.38 | 0.35 | 0.00 | 0.24 | 0.8357 | 3.111 |
| Diethyl sulfide | -1.07 | 0.37 | 0.38 | 0.00 | 0.32 | 0.8357 | 3.104 |
| Di-n-propyl sulfide | -0.94 | 0.36 | 0.38 | 0.00 | 0.32 | 1.1175 | 4.120 |
| Diisopropyl sulfide | -0.89 | 0.33 | 0.32 | 0.00 | 0.37 | 1.1175 | 3.600 |
| Diethyl disulfide | -1.20 | 0.67 | 0.48 | 0.00 | 0.29 | 0.9990 | 4.210 |
| Sulfur hexafluoride | 2.23 | -0.60 | -0.20 | 0.00 | 0.00 | 0.4643 | -0.120 |
| Triethyl phosphate | -5.53 | 0.00 | 1.00 | 0.00 | 1.06 | 1.3934 | 4.750 |
| N-Methylpiperidine | -2.85 | 0.32 | 0.34 | 0.00 | 0.70 | 0.9452 | 3.330 |
| Benzene | -0.63 | 0.61 | 0.52 | 0.00 | 0.14 | 0.7164 | 2.786 |
| Toluene | -0.65 | 0.60 | 0.52 | 0.00 | 0.14 | 0.8573 | 3.325 |
| Ethylbenzene | -0.58 | 0.61 | 0.51 | 0.00 | 0.15 | 0.9982 | 3.778 |
| o-Xylene | -0.66 | 0.66 | 0.56 | 0.00 | 0.16 | 0.9982 | 3.939 |
| m-Xylene | -0.61 | 0.62 | 0.52 | 0.00 | 0.16 | 0.9982 | 3.839 |
| p-Xylene | -0.59 | 0.61 | 0.52 | 0.00 | 0.16 | 0.9982 | 3.839 |
| n-Propylbenzene | -0.39 | 0.60 | 0.50 | 0.00 | 0.15 | 1.1390 | 4.230 |
| Isopropylbenzene | -0.22 | 0.60 | 0.49 | 0.00 | 0.16 | 1.1391 | 4.084 |
| 1,2,3-Trimethylbenzene | -0.89 | 0.73 | 0.61 | 0.00 | 0.19 | 1.1390 | 4.565 |
| 1,2,4-Trimethylbenzene | -0.63 | 0.68 | 0.56 | 0.00 | 0.19 | 1.1391 | 4.441 |
| 1,3,5-Trimethylbenzene | -0.66 | 0.65 | 0.52 | 0.00 | 0.19 | 1.1391 | 4.344 |
| 2-Ethyltoluene | -0.76 | 0.68 | 0.55 | 0.00 | 0.18 | 1.1391 | 4.346 |
| 4-Ethyltoluene | -0.70 | 0.63 | 0.51 | 0.00 | 0.18 | 1.1391 | 4.289 |
| n-Butylbenzene | -0.29 | 0.60 | 0.51 | 0.00 | 0.15 | 1.2800 | 4.730 |
| Isobutylbenzene | 0.12 | 0.58 | 0.47 | 0.00 | 0.15 | 1.2800 | 4.500 |
| sec-Butylbenzene | -0.33 | 0.60 | 0.48 | 0.00 | 0.16 | 1.2800 | 4.506 |
| tert-Butylbenzene | -0.32 | 0.62 | 0.49 | 0.00 | 0.16 | 1.2800 | 4.413 |
| 4-Isopropyltoluene | -0.50 | 0.61 | 0.49 | 0.00 | 0.19 | 1.2800 | 4.590 |
| n-Pentylbenzene | -0.17 | 0.59 | 0.51 | 0.00 | 0.15 | 1.4209 | 5.230 |
| n-Hexylbenzene | -0.03 | 0.59 | 0.50 | 0.00 | 0.15 | 1.5618 | 5.720 |
| Styrene | -0.91 | 0.85 | 0.65 | 0.00 | 0.16 | 0.9552 | 3.856 |
| a-Methylstyrene | -0.91 | 0.85 | 0.64 | 0.00 | 0.19 | 1.0961 | 4.292 |
| Biphenyl | -1.95 | 1.36 | 0.99 | 0.00 | 0.22 | 1.3242 | 6.014 |
| Naphthalene | -1.76 | 1.34 | 0.92 | 0.00 | 0.20 | 1.0854 | 5.161 |
| 1-Methylnaphthalene | -1.79 | 1.34 | 0.90 | 0.00 | 0.20 | 1.2263 | 5.789 |
| 1,3-Dimethvlnaphthalene | -1.81 | 1.39 | 0.92 | 0.00 | 0.20 | 1.3672 | 6.236 |
| 1,4-Dimelbvlnaphthalene | -2.07 | 1.40 | 0.91 | 0.00 | 0.20 | 1.3672 | 6.339 |
| 2,3-Dimethylnaphthalene | -2.04 | 1.43 | 0.95 | 0.00 | 0.20 | 1.3672 | 6.291 |
| 2,6-Dimethylnaphthalene | -1.93 | 1.33 | 0.91 | 0.00 | 0.20 | 1.3672 | 6.226 |
| 1-Ethylnaphthalene | -1.76 | 1.37 | 0.87 | 0.00 | 0.20 | 1.3672 | 6.136 |
| Indane | -1.07 | 0.83 | 0.62 | 0.00 | 0.17 | 1.0305 | 4.590 |
| Acenaphthene | -2.31 | 1.60 | 1.04 | 0.00 | 0.20 | 1.2586 | 6.469 |
| Fluorene | -2.46 | 1.59 | 1.03 | 0.00 | 0.20 | 1.3565 | 6.922 |
| Fluorobenzene | -0.59 | 0.48 | 0.57 | 0.00 | 0.10 | 0.7341 | 2.788 |
| Benzotrifluoride | -0.18 | 0.23 | 0.48 | 0.00 | 0.10 | 0.9100 | 2.894 |
| Chlorobenzene | -0.82 | 0.72 | 0.65 | 0.00 | 0.07 | 0.8388 | 3.657 |
| 1,2-Dichlorobenzene | -1.00 | 0.87 | 0.78 | 0.00 | 0.04 | 0.9612 | 4.518 |
| 1,3-Dichlorobenzene | -0.72 | 0.85 | 0.73 | 0.00 | 0.02 | 0.9612 | 4.410 |
| 1,4-Dichlorob,enzene | -0.74 | 0.83 | 0.75 | 0.00 | 0.02 | 0.9612 | 4.435 |
| 1,2,3-Trichlerobenzene | -0.91 | 1.03 | 0.86 | 0.00 | 0.00 | 1.0836 | 5.419 |
| 1,2,4-Trichlorobenzene | -0.82 | 0.98 | 0.81 | 0.00 | 0.00 | 1.0836 | 5.248 |
| 1,3,5-Trichlorobenzene | -0.57 | 0.98 | 0.73 | 0.00 | 0.00 | 1.0836 | 5.045 |
| 1,2,3,4-Tetrachlorobenzene | -0.98 | 1.18 | 0.92 | 0.00 | 0.00 | 1.2060 | 6.171 |
| 1,2,3,5-Tetrachlorobenzene | -1.19 | 1.16 | 0.85 | 0.00 | 0.00 | 1.2060 | 5.922 |
| 1,2,4,5-Tetrachlorobenzene | -0.98 | 1.16 | 0.86 | 0.00 | 0.00 | 1.2060 | 5.926 |
| 2-Chlorotoluene | -0.84 | 0.76 | 0.65 | 0.00 | 0.07 | 0.9797 | 4.173 |
| Bromobenzene | -1.07 | 0.88 | 0.73 | 0.00 | 0.09 | 0.8914 | 4.041 |
| 4-Bromotoluene | -1.02 | 0.88 | 0.74 | 0.00 | 0.09 | 1.0323 | 4.586 |
| lodobenzene | -1.28 | 1.19 | 0.82 | 0.00 | 0.12 | 0.9746 | 4.502 |

| | | | | | | | |
|---|---|---|---|---|---|---|---|
| Methyl phenyl ether/ Anisol | -1.80 | 0.71 | 0.74 | 0.00 | 0.29 | 0.9160 | 3.890 |
| Ethyl phenyl ether | -1.63 | 0.68 | 0.70 | 0.00 | 0.32 | 1.0569 | 4.242 |
| Benzaldehyde | -2.95 | 0.82 | 1.00 | 0.00 | 0.39 | 0.8730 | 4.008 |
| 4-Methylbenzaldehyde | -3.13 | 0.86 | 1.00 | 0.00 | 0.42 | 1.0139 | 4.592 |
| Acetophenone | -3.36 | 0.82 | 1.01 | 0.00 | 0.49 | 1.0139 | 4.501 |
| 4-Methylacetophenone | -3.45 | 0.84 | 1.00 | 0.00 | 0.52 | 1.1548 | 5.080 |
| Methyl benzoate | -2.88 | 0.73 | 0.85 | 0.00 | 0.48 | 1.0726 | 4.704 |
| Ethyl benzoate | -2.67 | 0.69 | 0.85 | 0.00 | 0.46 | 1.2135 | 5.075 |
| Benzonitrile | -3.09 | 0.74 | 1.11 | 0.00 | 0.33 | 0.8711 | 4.039 |
| o-Toluidine 1,2 Methylaniline | -4.06 | 0.97 | 0.92 | 0.23 | 0.45 | 0.9571 | 4.442 |
| p-Toluidine 1,4 Methylaniline | -4.09 | 0.92 | 0.95 | 0.23 | 0.45 | 0.9571 | 4.452 |
| 2,6-Dimethylaniline | -3.82 | 0.97 | 0.89 | 0.20 | 0.46 | 1.0980 | 5.028 |
| 2-Chloroaniline | -3.60 | 1.03 | 0.92 | 0.25 | 0.31 | 0.9386 | 4.674 |
| 3-Chloroaniline | -4.27 | 1.05 | 1.10 | 0.30 | 0.30 | 0.9396 | 4.909 |
| 4-Chloroaniline | -4.33 | 1.06 | 1.13 | 0.30 | 0.32 | 0.9386 | 4.889 |
| 2-Methoxyaniline | -4.49 | 0.99 | 1.03 | 0.23 | 0.50 | 1.0158 | 4.818 |
| 3-Methoxyaniline | -5.35 | 1.03 | 1.22 | 0.25 | 0.55 | 1.0158 | 5.023 |
| 4-Methoxyaniline | -5.49 | 1.05 | 1.19 | 0.23 | 0.61 | 1.0158 | 4.949 |
| 2-Nitroaniline | -5.41 | 1.18 | 1.37 | 0.30 | 0.36 | 0.9904 | 5.627 |
| 3-Nitroaniline | -6.49 | 1.20 | 1.71 | 0.40 | 0.35 | 0.9904 | 5.880 |
| 4-Nitroaniline | -7.54 | 1.22 | 1.91 | 0.42 | 0.38 | 0.9904 | 6.343 |
| 1-Naphthylamine | -5.34 | 1.67 | 1.26 | 0.20 | 0.57 | 1.1850 | 6.490 |
| 2-Naphthylamine | -5.48 | 1.67 | 1.28 | 0.22 | 0.55 | 1.1850 | 6.540 |
| N-Methylaniline | -3.44 | 0.95 | 0.90 | 0.17 | 0.43 | 0.9571 | 4.478 |
| N,N-Dimethylaniline | -2.53 | 0.96 | 0.84 | 0.00 | 0.42 | 1.0980 | 4.701 |
| Nitrobenzene | -3.02 | 0.87 | 1.11 | 0.00 | 0.28 | 0.8906 | 4.557 |
| 2-Nitrotoluene | -2.63 | 0.87 | 1.11 | 0.00 | 0.28 | 1.0315 | 4.878 |
| 3-Nitrotoluene | -2.53 | 0.87 | 1.10 | 0.00 | 0.28 | 1.0315 | 5.097 |
| Benzamide | -8.07 | 0.99 | 1.50 | 0.49 | 0.67 | 0.9728 | 5.767 |
| Phenol | -4.85 | 0.81 | 0.89 | 0.60 | 0.31 | 0.7751 | 3.766 |
| O-Cresol | -4.31 | 0.84 | 0.86 | 0.52 | 0.31 | 0.9160 | 4.218 |
| P-Cresol | -4.50 | 0.82 | 0.87 | 0.57 | 0.32 | 0.9160 | 4.312 |
| 2,3-Dimethylphenol | -4.52 | 0.85 | 0.81 | 0.53 | 0.36 | 1.0569 | 4.952 |
| 2,4-Dimethylphenol | -4.41 | 0.84 | 0.80 | 0.53 | 0.39 | 1.0569 | 4.770 |
| 2,5-Dimethylphenol | -4.34 | 0.94 | 0.79 | 0.54 | 0.37 | 1.0569 | 4.774 |
| 2,6-Dimethylphenot | -3.86 | 0.86 | 0.79 | 0.39 | 0.39 | 1.0569 | 4.680 |
| 3,4-Dimethylphenol | -4.77 | 0.83 | 0.86 | 0.56 | 0.39 | 1.0569 | 4.980 |
| 3,5-Dimethylphenol | -4.60 | 0.82 | 0.84 | 0.57 | 0.36 | 1.0569 | 4.856 |
| 3-Ethylphenol | -4.59 | 0.81 | 0.91 | 0.55 | 0.37 | 1.0569 | 4.741 |
| 4-Ethylphenol | -4.50 | 0.80 | 0.90 | 0.55 | 0.36 | 1.0569 | 4.737 |
| 4-n-Propylphenol | -4.33 | 0.79 | 0.88 | 0.55 | 0.37 | 1.1978 | 5.185 |
| 4-tert-Butylphenol | -4.34 | 0.81 | 0.89 | 0.56 | 0.39 | 1.3387 | 5.264 |
| 2-Fluorophenol | -3.88 | 0.66 | 0.69 | 0.61 | 0.26 | 0.7928 | 3.453 |
| 4-Fluorophenol | -4.54 | 0.67 | 0.97 | 0.63 | 0.23 | 0.7928 | 3.844 |
| 2-Chlorophenol | -3.34 | 0.85 | 0.88 | 0.32 | 0.31 | 0.8975 | 4.178 |
| 3-Chlorophenol | -4.85 | 0.91 | 1.06 | 0.69 | 0.15 | 0.8975 | 4.773 |
| 4-Chlorophenol | -5.16 | 0.92 | 1.08 | 0.67 | 0.21 | 0.8975 | 4.775 |
| 4-Chloro-3-methylphenol | -4.98 | 0.92 | 1.02 | 0.65 | 0.23 | 1.0384 | 5.290 |
| 4-Bromophenol | -5.23 | 1.08 | 1.17 | 0.67 | 0.20 | 0.9501 | 5.135 |
| 2-lodophenol | -4.55 | 1.36 | 1.00 | 0.40 | 0.35 | 1.0335 | 4.964 |
| 2-Methoxyphenol | -4.09 | 0.84 | 0.91 | 0.22 | 0.52 | 0.9747 | 4.449 |
| 3-Methoxyphenol | -5.62 | 0.88 | 1.17 | 0.59 | 0.38 | 0.9747 | 4.803 |
| 3-Cyanophenol | -7.08 | 0.93 | 1.55 | 0.77 | 0.28 | 0.9298 | 5.180 |
| 4-Cyanophenol | -7.46 | 0.94 | 1.63 | 0.79 | 0.30 | 0.9298 | 5.420 |
| 2-Nitrophenol | -3.36 | 1.02 | 1.05 | 0.05 | 0.37 | 0.9493 | 4.760 |
| 3-Nitrophenol | -7.06 | 1.05 | 1.57 | 0.79 | 0.23 | 0.9493 | 5.692 |
| 4-Nitrophenol | -7.81 | 1.07 | 1.72 | 0.82 | 0.26 | 0.9493 | 5.876 |

| | | | | | | | |
|---|---|---|---|---|---|---|---|
| 1-Naphthol | -5.63 | 1.52 | 1.05 | 0.61 | 0.37 | 1.1440 | 6.130 |
| 2-Naphthol | -5.95 | 1.52 | 1.08 | 0.61 | 0.40 | 1.1440 | 6.200 |
| Benzyl alcohol | -4.86 | 0.80 | 0.87 | 0.33 | 0.56 | 0.9160 | 4.221 |
| 2-Phenylethanol | -4.98 | 0.81 | 0.91 | 0.30 | 0.65 | 1.0569 | 4.628 |
| 3-Phenylpropanol | -5.08 | 0.82 | 0.90 | 0.30 | 0.67 | 1.1978 | 5.180 |
| Thiophenol | -1.87 | 1.00 | 0.80 | 0.09 | 0.16 | 0.8799 | 4.110 |
| Phenyl rnethyl sulfide | -2.00 | 1.07 | 0.92 | 0.00 | 0.26 | 1.0280 | 4.659 |
| Pyridine | -3.44 | 0.63 | 0.84 | 0.00 | 0.52 | 0.6753 | 3.022 |
| 2-Methylpyridine | -3.40 | 0.60 | 0.75 | 0.00 | 0.57 | 0.8162 | 3.422 |
| 3-Methylpyridine | -3.50 | 0.63 | 0.81 | 0.00 | 0.54 | 0.8162 | 3.631 |
| 4-Methylpyridine | -3.62 | 0.63 | 0.82 | 0.00 | 0.55 | 0.8162 | 3.640 |
| 2,3-Dimethylpyridine | -3.54 | 0.66 | 0.77 | 0.00 | 0.62 | 0.9571 | 4.045 |
| 2,4-Dimethylpyridine | -3.57 | 0.63 | 0.76 | 0.00 | 0.63 | 0.9571 | 4.006 |
| 2,5-Dimethylpyridine | -3.46 | 0.63 | 0.74 | 0.00 | 0.62 | 0.9571 | 3.986 |
| 2,6-Dimethylpyridine | -3.37 | 0.61 | 0.70 | 0.00 | 0.62 | 0.9571 | 3.760 |
| 3,4-Dimethylpyridine | -3.83 | 0.68 | 0.85 | 0.00 | 0.61 | 0.9571 | 4.317 |
| 3,5-Dimelhylpyridine | -3.55 | 0.66 | 0.79 | 0.00 | 0.60 | 0.9571 | 4.214 |
| 2-Ethylpyridine | -3.18 | 0.61 | 0.70 | 0.00 | 0.59 | 0.9571 | 3.844 |
| 3-Ethylpyridine | -3.37 | 0.64 | 0.79 | 0.00 | 0.57 | 0.9571 | 4.093 |
| 4-Ethylpyridine | -3.47 | 0.63 | 0.80 | 0.00 | 0.57 | 0.9571 | 4.124 |
| 2-Chloropyridine | -3.22 | 0.74 | 1.03 | 0.00 | 0.37 | 0.7977 | 3.875 |
| 3-Chloropyridine | -2.94 | 0.73 | 0.83 | 0.00 | 0.41 | 0.7977 | 3.783 |
| 3-Cyanopyridine | -4.95 | 0.75 | 1.26 | 0.00 | 0.62 | 0.8300 | 4.164 |
| 4-Cyanopyridine | -4.42 | 0.75 | 1.21 | 0.00 | 0.59 | 0.8300 | 4.033 |
| 3-Formylpyridine | -5.21 | 0.82 | 1.16 | 0.00 | 0.76 | 0.8319 | 4.258 |
| 3-Acetylpyridine | -6.06 | 0.80 | 1.17 | 0.00 | 0.90 | 0.9728 | 4.880 |
| 4-Acetylpyridine | -5.59 | 0.77 | 1.13 | 0.00 | 0.84 | 0.9728 | 4.660 |
| Quinoline | -4.20 | 1.27 | 0.97 | 0.00 | 0.54 | 1.0443 | 5.457 |
| 2-Methylpyrazine | -4.04 | 0.63 | 0.86 | 0.00 | 0.67 | 0.7751 | 3.254 |
| Thiophene | -1.04 | 0.69 | 0.56 | 0.00 | 0.15 | 0.6411 | 2.819 |
| 2-Methylthiophene | -1.01 | 0.69 | 0.56 | 0.00 | 0.16 | 0.7820 | 3.308 |

Table S3. Data and descriptors for log $K_{oilw}$.[7]

| | Log $K_{oilw}$ | *E* | *S* | *A* | *B* | *V* | *L* |
|---|---|---|---|---|---|---|---|
| 1,1,1,2-Tetrachloroethane | 2.85 | 0.54 | 0.63 | 0.10 | 0.08 | 0.88 | 3.64 |
| 1,1,1-Trichloroethane | 2.66 | 0.37 | 0.41 | 0.00 | 0.09 | 0.76 | 2.73 |
| 1,1,2,2-Tetrachloroethane | 2.46 | 0.60 | 0.76 | 0.16 | 0.12 | 0.88 | 3.80 |
| 1,1,2-Trichloroethane | 2.09 | 0.50 | 0.68 | 0.13 | 0.13 | 0.76 | 3.29 |
| 1,1-Dichloro-1-fluoroethane | 1.34 | 0.84 | 0.43 | 0.01 | 0.05 | 0.67 | 1.92 |
| 1,1-Dichloroethane | 1.74 | 0.32 | 0.49 | 0.10 | 0.10 | 0.64 | 2.32 |
| 1,1-Dichloroethene | 2.19 | 0.36 | 0.34 | 0.00 | 0.05 | 0.59 | 2.11 |
| 1,2,3-Trimethylbenzene | 3.32 | 0.73 | 0.61 | 0.00 | 0.19 | 1.14 | 4.57 |
| 1,2,4-Trifluorobenzene | 2.67 | 0.31 | 0.65 | 0.00 | 0.02 | 0.83 | 2.85 |
| 1,2,4-Trimethylbenzene | 3.43 | 0.68 | 0.56 | 0.00 | 0.19 | 1.14 | 4.44 |
| 1,2-Dibromoethane | 1.67 | 0.75 | 0.76 | 0.10 | 0.17 | 0.74 | 3.38 |
| 1,2-Dichlorobenzene | 3.71 | 0.87 | 0.78 | 0.00 | 0.04 | 0.96 | 4.32 |
| 1,2-Dichloroethane | 1.60 | 0.42 | 0.64 | 0.10 | 0.11 | 0.64 | 2.57 |
| 1,2-Dichloropropane | 1.99 | 0.37 | 0.68 | 0.00 | 0.15 | 0.78 | 2.87 |
| 1,2-Difluorobenzene | 2.62 | 0.39 | 0.63 | 0.00 | 0.06 | 0.79 | 2.84 |
| 1,3,5-Trifluorobenzene | 2.93 | 0.27 | 0.49 | 0.00 | 0.00 | 0.83 | 2.66 |
| 1,3,5-Trimethylbenzene | 3.49 | 0.65 | 0.52 | 0.00 | 0.19 | 1.14 | 4.34 |
| 1,3-Dichlorobenzene | 3.84 | 0.85 | 0.73 | 0.00 | 0.02 | 0.96 | 4.41 |
| 1,4-Difluorobenzene | 2.58 | 0.38 | 0.60 | 0.00 | 0.06 | 0.79 | 2.77 |
| 1-Bromo-2-chloroethane | 2.07 | 0.08 | 0.70 | 0.10 | 0.09 | 0.69 | 2.98 |
| 1-Butanol | 0.02 | 0.22 | 0.42 | 0.37 | 0.48 | 0.73 | 2.60 |

| | | | | | | | |
|---|---|---|---|---|---|---|---|
| 1-Chlorobutane | 2.78 | 0.21 | 0.40 | 0.00 | 0.10 | 0.79 | 2.72 |
| 1-Chloropentane | 3.48 | 0.21 | 0.38 | 0.00 | 0.09 | 0.94 | 3.22 |
| 1-Chloropropane | 2.22 | 0.22 | 0.40 | 0.00 | 0.10 | 0.65 | 2.20 |
| 1-Hexanol | 1.36 | 0.21 | 0.42 | 0.37 | 0.48 | 1.01 | 3.61 |
| 1-Methoxy-2-propanol | -1.53 | 0.22 | 0.54 | 0.31 | 0.82 | 0.79 | 2.66 |
| 1-Nitropropane | 0.97 | 0.24 | 0.95 | 0.00 | 0.31 | 0.71 | 2.89 |
| 1-Pentanol | 0.54 | 0.22 | 0.42 | 0.37 | 0.48 | 0.87 | 3.11 |
| 1-Propanol | -0.48 | 0.24 | 0.42 | 0.37 | 0.48 | 0.59 | 2.03 |
| 2,2,4-Trimethylpentane | 4.64 | 0.00 | 0.00 | 0.00 | 0.00 | 1.24 | 3.11 |
| 2,2-Dichloro-1,1,1-trifluoroethane | 1.81 | -0.16 | 0.40 | 0.22 | 0.00 | 0.75 | 1.75 |
| 2,2-Dimethylbutane | 3.79 | 0.00 | 0.00 | 0.00 | 0.00 | 0.95 | 2.35 |
| 2,3,4-Trimethylpentane | 5.05 | 0.00 | 0.00 | 0.00 | 0.00 | 1.24 | 3.48 |
| 2-Butoxyethanol | -0.21 | 0.20 | 0.50 | 0.30 | 0.83 | 1.07 | 3.81 |
| 2-Chloropropane | 2.03 | 0.18 | 0.35 | 0.00 | 0.12 | 0.65 | 1.97 |
| 2-Ethoxyethanol | -1.27 | 0.24 | 0.52 | 0.31 | 0.81 | 0.79 | 2.79 |
| 2-Fluoropropane | 1.38 | 0.00 | 0.32 | 0.00 | 0.10 | 0.57 | 1.07 |
| 2-Heptanone | 1.81 | 0.12 | 0.68 | 0.00 | 0.51 | 1.11 | 3.76 |
| 2-Hexanone | 1.19 | 0.14 | 0.68 | 0.00 | 0.51 | 0.97 | 3.29 |
| 2-Isopropoxyethanol | -1.16 | 0.20 | 0.47 | 0.30 | 0.91 | 0.93 | 3.17 |
| 2-Methoxyethanol | -1.67 | 0.27 | 0.50 | 0.30 | 0.84 | 0.79 | 2.49 |
| 2-Methyl-1-propanol | -0.06 | 0.22 | 0.39 | 0.37 | 0.48 | 0.73 | 2.41 |
| 2-Methyl-2-propanol | -0.73 | 0.28 | 0.30 | 0.31 | 0.60 | 0.73 | 1.96 |
| 2-Methylpentane | 3.99 | 0.00 | 0.00 | 0.00 | 0.00 | 0.95 | 2.50 |
| 2-Nitropropane | 0.61 | 0.22 | 0.92 | 0.00 | 0.33 | 0.71 | 2.55 |
| 2-Pentanone | 0.65 | 0.14 | 0.68 | 0.00 | 0.51 | 0.83 | 2.76 |
| 2-Propanol | -0.82 | 0.21 | 0.36 | 0.33 | 0.56 | 0.59 | 1.76 |
| 3-Methyl-1-butanol | 0.41 | 0.19 | 0.39 | 0.37 | 0.48 | 0.87 | 3.01 |
| 3-Methylhexane | 4.61 | 0.00 | 0.00 | 0.00 | 0.00 | 1.09 | 3.04 |
| 3-Methylpentane | 4.07 | 0.00 | 0.00 | 0.00 | 0.00 | 0.95 | 2.58 |
| 3-Pentanone | 0.56 | 0.15 | 0.66 | 0.00 | 0.51 | 0.83 | 2.81 |
| 4-Methyl-2-pentanone | 1.05 | 0.11 | 0.65 | 0.00 | 0.51 | 0.97 | 3.09 |
| Acetone | -0.32 | 0.18 | 0.70 | 0.04 | 0.49 | 0.55 | 1.70 |
| Allylbenzene | 2.96 | 0.72 | 0.60 | 0.00 | 0.22 | 1.10 | 4.14 |
| Benzene | 2.12 | 0.61 | 0.52 | 0.00 | 0.14 | 0.72 | 2.79 |
| Bromochloromethane | 1.49 | 0.54 | 0.80 | 0.01 | 0.06 | 0.55 | 2.45 |
| 2-Butanone | 0.18 | 0.17 | 0.70 | 0.00 | 0.51 | 0.69 | 2.29 |
| Butane | 3.03 | 0.00 | 0.00 | 0.00 | 0.00 | 0.67 | 1.62 |
| Butyl acetate | 1.59 | 0.07 | 0.60 | 0.00 | 0.45 | 1.03 | 3.35 |
| Carbon tetrachloride | 3.18 | 0.46 | 0.38 | 0.00 | 0.00 | 0.74 | 2.82 |
| 1,2-Dichlorotetrafluoroethane | 3.00 | -0.19 | 0.05 | 0.00 | 0.00 | 0.79 | 1.43 |
| 1,1,2,2,3,3,4,4-Octafluorobutane | 2.50 | -0.79 | 0.08 | 0.15 | 0.15 | 0.97 | 1.46 |
| 1,1,2,2,3,3-Hexafluoropropane | 1.49 | -0.59 | 0.21 | 0.15 | 0.10 | 0.76 | 0.62 |
| 1,1,2,2-Tetrafluoroethane | 0.92 | -0.39 | 0.24 | 0.10 | 0.12 | 0.54 | 0.39 |
| 1,1-Difluoroethane | 0.91 | -0.25 | 0.49 | 0.04 | 0.05 | 0.47 | 0.52 |
| Halothane | 2.28 | 0.10 | 0.39 | 0.13 | 0.05 | 0.80 | 1.95 |
| Teflurane | 1.80 | -0.07 | 0.21 | 0.20 | 0.02 | 0.72 | 1.37 |
| 1,1,1,2-Tetrafluoroethane | 1.03 | -0.39 | 0.16 | 0.16 | 0.05 | 0.54 | 0.40 |
| Fluroxene | 1.26 | 0.18 | 0.30 | 0.00 | 0.27 | 0.80 | 1.60 |
| Carbon tetrafluoride | 1.12 | -0.58 | -0.26 | 0.00 | 0.00 | 0.40 | -0.82 |
| 1,3-Difluoropropane | 0.66 | -0.20 | 0.55 | 0.12 | 0.21 | 0.61 | 1.35 |
| Enflurane | 2.03 | -0.24 | 0.54 | 0.01 | 0.10 | 0.90 | 2.01 |
| Isoflurane | 1.99 | -0.24 | 0.56 | 0.00 | 0.08 | 0.90 | 1.97 |
| Desflurane | 1.88 | -0.47 | 0.38 | 0.05 | 0.04 | 0.82 | 0.99 |
| Sevoflurane | 1.87 | -0.47 | 0.56 | 0.00 | 0.10 | 0.99 | 1.50 |
| Chlorobenzene | 2.73 | 0.72 | 0.65 | 0.00 | 0.07 | 0.84 | 3.66 |
| Chlorodibromomethane | 2.12 | 0.78 | 0.68 | 0.12 | 0.10 | 0.72 | 3.30 |
| Chloroethane | 1.58 | 0.23 | 0.40 | 0.00 | 0.10 | 0.51 | 1.68 |

| | | | | | | | |
|---|---|---|---|---|---|---|---|
| Chloroform | 2.02 | 0.43 | 0.49 | 0.15 | 0.02 | 0.62 | 2.48 |
| cis-1,2-Dichloroethene | 1.59 | 0.44 | 0.61 | 0.11 | 0.05 | 0.59 | 2.44 |
| Cycloheptane | 4.82 | 0.35 | 0.10 | 0.00 | 0.00 | 0.99 | 3.70 |
| Cyclohexane | 4.05 | 0.31 | 0.10 | 0.00 | 0.00 | 0.85 | 2.96 |
| Cyclopentane | 3.50 | 0.26 | 0.10 | 0.00 | 0.00 | 0.70 | 2.48 |
| Cyclopropane | 1.73 | 0.41 | 0.23 | 0.00 | 0.00 | 0.42 | 1.31 |
| Decane | 6.39 | 0.00 | 0.00 | 0.00 | 0.00 | 1.52 | 4.69 |
| Dibromethane | 1.76 | 0.71 | 0.69 | 0.11 | 0.07 | 0.60 | 2.89 |
| Dichloromethane | 1.28 | 0.39 | 0.57 | 0.10 | 0.05 | 0.49 | 2.02 |
| Diethyl ether | 0.79 | 0.00 | 0.25 | 0.00 | 0.45 | 0.73 | 2.02 |
| Difluoromethane | 0.53 | -0.32 | 0.49 | 0.06 | 0.05 | 0.32 | 0.04 |
| Divinyl ether | 1.69 | 0.26 | 0.39 | 0.00 | 0.13 | 0.64 | 1.76 |
| Ethane | 1.73 | 0.00 | 0.00 | 0.00 | 0.00 | 0.39 | 0.49 |
| Ethanol | -1.14 | 0.25 | 0.42 | 0.37 | 0.48 | 0.45 | 1.49 |
| Ethene | 0.84 | 0.11 | 0.10 | 0.00 | 0.07 | 0.35 | 0.29 |
| Ethyl acetate | 0.46 | 0.11 | 0.62 | 0.00 | 0.45 | 0.75 | 2.31 |
| Ethyl tert-butyl ether | 1.37 | -0.02 | 0.16 | 0.00 | 0.60 | 1.01 | 2.72 |
| Ethyl tert-pentyl ether | 1.93 | 0.00 | 0.16 | 0.00 | 0.61 | 1.15 | 3.26 |
| Ethylbenzene | 3.14 | 0.61 | 0.51 | 0.00 | 0.15 | 1.00 | 3.78 |
| Fluorobenzene | 2.41 | 0.48 | 0.57 | 0.00 | 0.10 | 0.75 | 2.79 |
| Fluoroethane | 0.62 | 0.05 | 0.35 | 0.00 | 0.10 | 0.43 | 0.58 |
| Fluorochloromethane | 0.77 | 0.04 | 0.61 | 0.07 | 0.04 | 0.41 | 0.98 |
| Heptane | 4.71 | 0.00 | 0.00 | 0.00 | 0.00 | 1.09 | 3.17 |
| Hexafluorobenzene | 2.45 | 0.09 | 0.56 | 0.00 | 0.01 | 0.82 | 2.35 |
| Hexane | 4.11 | 0.00 | 0.00 | 0.00 | 0.00 | 0.95 | 2.67 |
| Isobutyl acetate | 1.60 | 0.05 | 0.57 | 0.00 | 0.47 | 1.03 | 3.16 |
| Isopentyl acetate | 2.11 | 0.05 | 0.57 | 0.00 | 0.47 | 1.17 | 3.74 |
| Isopropyl acetate | 1.02 | 0.06 | 0.57 | 0.00 | 0.47 | 0.89 | 2.55 |
| Isopropylbenzene | 3.52 | 0.60 | 0.49 | 0.00 | 0.16 | 1.14 | 4.08 |
| Methoxyflurane | 2.11 | 0.11 | 0.67 | 0.07 | 0.14 | 0.91 | 2.86 |
| Methane | 0.75 | 0.00 | 0.00 | 0.00 | 0.00 | 0.25 | -0.32 |
| Methanol | -1.95 | 0.28 | 0.44 | 0.43 | 0.47 | 0.31 | 0.97 |
| Methyl acetate | -0.02 | 0.14 | 0.64 | 0.00 | 0.45 | 0.61 | 1.91 |
| Methyl chloride | 0.80 | 0.25 | 0.43 | 0.00 | 0.08 | 0.37 | 1.16 |
| Methylcyclopentane | 4.00 | 0.23 | 0.10 | 0.00 | 0.00 | 0.85 | 2.91 |
| Methylpentafluorobenzene | 3.27 | 0.06 | 0.59 | 0.00 | 0.01 | 1.05 | 3.24 |
| m-Methylstyrene | 3.23 | 0.87 | 0.65 | 0.00 | 0.18 | 1.10 | 4.38 |
| m-Xylene | 3.16 | 0.62 | 0.52 | 0.00 | 0.16 | 1.00 | 3.84 |
| Nonane | 5.82 | 0.00 | 0.00 | 0.00 | 0.00 | 1.38 | 4.18 |
| o-Xylene | 3.12 | 0.66 | 0.56 | 0.00 | 0.16 | 1.00 | 3.94 |
| p-Xylene | 3.16 | 0.61 | 0.52 | 0.00 | 0.16 | 1.00 | 3.84 |
| Pentachloroethane | 2.93 | 0.65 | 0.66 | 0.17 | 0.06 | 1.00 | 4.27 |
| Pentafluorobenzene | 2.31 | 0.15 | 0.68 | 0.00 | 0.02 | 0.90 | 2.58 |
| Pentane | 3.49 | 0.00 | 0.00 | 0.00 | 0.00 | 0.81 | 2.16 |
| Pentyl acetate | 2.11 | 0.07 | 0.60 | 0.00 | 0.45 | 1.17 | 3.84 |
| p-Methylstyrene | 3.21 | 0.87 | 0.65 | 0.00 | 0.18 | 1.10 | 4.40 |
| Propane | 2.35 | 0.00 | 0.00 | 0.00 | 0.00 | 0.53 | 1.05 |
| Propyl acetate | 1.02 | 0.09 | 0.60 | 0.00 | 0.45 | 0.89 | 2.82 |
| Propylbenzene | 3.65 | 0.60 | 0.50 | 0.00 | 0.15 | 1.14 | 4.23 |
| Tetrachloroethene | 3.57 | 0.64 | 0.44 | 0.00 | 0.00 | 0.84 | 3.58 |
| Toluene | 2.67 | 0.60 | 0.52 | 0.00 | 0.14 | 0.86 | 3.33 |
| trans-1,2-Dichloroethene | 2.06 | 0.43 | 0.41 | 0.09 | 0.05 | 0.59 | 2.28 |
| Trichloroethene | 2.80 | 0.52 | 0.37 | 0.08 | 0.03 | 0.71 | 3.00 |
| Tridecane | 8.16 | 0.00 | 0.00 | 0.00 | 0.00 | 1.94 | 6.20 |
| Triethylamine | 1.04 | 0.10 | 0.15 | 0.00 | 0.79 | 1.05 | 3.04 |
| Undecane | 7.03 | 0.00 | 0.00 | 0.00 | 0.00 | 1.66 | 5.19 |
| alpha-Pinene | 4.58 | 0.45 | 0.14 | 0.00 | 0.12 | 1.26 | 4.31 |

| | | | | | | | |
|---|---|---|---|---|---|---|---|
| 1,2-dimethoxyethane | -0.33 | 0.12 | 0.67 | 0.00 | 0.68 | 0.79 | 2.65 |
| 1,4-dioxane | -0.24 | 0.33 | 0.75 | 0.00 | 0.64 | 0.68 | 2.89 |
| 1-heptanol | 1.84 | 0.21 | 0.42 | 0.37 | 0.48 | 1.15 | 4.12 |
| 2-methylpyridine | 0.61 | 0.60 | 0.75 | 0.00 | 0.58 | 0.82 | 3.42 |
| 3-methylpyridine | 0.84 | 0.63 | 0.81 | 0.00 | 0.54 | 0.82 | 3.63 |
| 4-methylpyridine | 0.82 | 0.63 | 0.82 | 0.00 | 0.54 | 0.82 | 3.64 |
| benzyl alcohol | 0.12 | 0.80 | 0.87 | 0.39 | 0.56 | 0.92 | 4.22 |
| bromobenzene | 3.27 | 0.88 | 0.73 | 0.00 | 0.09 | 0.89 | 4.04 |
| butyl formate | 1.46 | 0.12 | 0.63 | 0.00 | 0.38 | 0.89 | 2.96 |
| butyl propanoate | 2.35 | 0.06 | 0.56 | 0.00 | 0.47 | 1.17 | 3.83 |
| butylbenzene | 4.13 | 0.60 | 0.51 | 0.00 | 0.15 | 1.28 | 4.73 |
| 1,1-difluoro-2-chloroethene | 1.63 | -0.34 | 0.29 | 0.15 | 0.00 | 0.55 | 0.72 |
| 1-chloro-2,2,2-trifluoroethane | 1.34 | 0.01 | 0.40 | 0.15 | 0.00 | 0.63 | 1.17 |
| bis-(2,2,2-trifluoroethyl)ether | 1.92 | -0.51 | 0.03 | 0.08 | 0.36 | 0.96 | 1.42 |
| cyclohexene | 3.33 | 0.40 | 0.28 | 0.00 | 0.09 | 0.80 | 2.95 |
| cyclopentanone | 0.39 | 0.37 | 0.86 | 0.00 | 0.52 | 0.72 | 3.22 |
| difluorochloromethane | 0.72 | 0.00 | 0.25 | 0.20 | 0.00 | 0.45 | 0.69 |
| diisopropyl ether | 1.29 | -0.06 | 0.16 | 0.00 | 0.58 | 1.01 | 2.53 |
| dimethoxymethane | 0.18 | 0.10 | 0.46 | 0.00 | 0.52 | 0.65 | 1.89 |
| dimethyl ether | 0.13 | 0.00 | 0.27 | 0.00 | 0.41 | 0.45 | 1.29 |
| dimethylacetamide | -1.25 | 0.36 | 1.35 | 0.00 | 0.77 | 0.79 | 3.64 |
| dimethylformamide | -1.57 | 0.37 | 1.31 | 0.00 | 0.74 | 0.65 | 3.17 |
| di-n-butyl ether | 3.14 | 0.00 | 0.25 | 0.00 | 0.45 | 1.29 | 3.92 |
| dodecane | 7.59 | 0.00 | 0.00 | 0.00 | 0.00 | 1.80 | 5.70 |
| ethyl formate | 0.16 | 0.15 | 0.66 | 0.00 | 0.38 | 0.61 | 1.85 |
| ethyl propanoate | 1.13 | 0.09 | 0.58 | 0.00 | 0.45 | 0.89 | 2.81 |
| iodoethane | 1.85 | 0.64 | 0.40 | 0.00 | 0.14 | 0.65 | 2.57 |
| fluorotrichloromethane | 2.28 | 0.21 | 0.24 | 0.00 | 0.07 | 0.65 | 1.95 |
| methyl formate | -0.48 | 0.19 | 0.68 | 0.00 | 0.38 | 0.46 | 1.29 |
| methylcyclohexane | 4.54 | 0.24 | 0.06 | 0.00 | 0.00 | 0.99 | 3.32 |
| nitroethane | 0.45 | 0.27 | 0.95 | 0.02 | 0.33 | 0.56 | 2.41 |
| nitromethane | -0.02 | 0.31 | 0.95 | 0.06 | 0.31 | 0.42 | 1.89 |
| N,N-dimethylaniline | 2.29 | 0.96 | 0.81 | 0.00 | 0.41 | 1.10 | 4.70 |
| pentadecane | 9.31 | 0.00 | 0.00 | 0.00 | 0.00 | 2.22 | 7.21 |
| piperidine | 1.01 | 0.42 | 0.46 | 0.13 | 0.68 | 0.80 | 3.30 |
| propyl bromide | 2.47 | 0.37 | 0.40 | 0.00 | 0.12 | 0.71 | 2.62 |
| propyl formate | 0.87 | 0.13 | 0.63 | 0.00 | 0.38 | 0.75 | 2.43 |
| pyridine | 0.15 | 0.63 | 0.84 | 0.00 | 0.52 | 0.68 | 3.02 |
| tetradecane | 8.74 | 0.00 | 0.00 | 0.00 | 0.00 | 2.08 | 6.71 |
| tetrahydrofuran | 0.62 | 0.29 | 0.52 | 0.00 | 0.48 | 0.62 | 2.64 |
| halopropane | 2.91 | -0.07 | 0.28 | 0.20 | 0.00 | 0.86 | 2.03 |
| CF3CHFOCHFCl | 2.15 | -0.24 | 0.50 | 0.00 | 0.11 | 0.90 | 1.87 |
| Cyclooctane | 5.34 | 0.41 | 0.10 | 0.00 | 0.00 | 1.13 | 4.33 |
| Octan-1-ol | 2.46 | 0.20 | 0.42 | 0.37 | 0.48 | 1.29 | 4.62 |
| Nonan-1-ol | 3.01 | 0.19 | 0.42 | 0.37 | 0.48 | 1.44 | 5.12 |
| Hexanal | 1.92 | 0.15 | 0.65 | 0.00 | 0.45 | 0.97 | 3.36 |
| Heptanal | 2.46 | 0.14 | 0.65 | 0.00 | 0.45 | 1.11 | 3.87 |
| Octanal | 3.01 | 0.16 | 0.65 | 0.00 | 0.45 | 1.25 | 4.36 |
| Nonanal | 3.42 | 0.15 | 0.65 | 0.00 | 0.45 | 1.39 | 4.86 |
| 1-Chloroheptane | 4.37 | 0.19 | 0.40 | 0.00 | 0.10 | 1.22 | 4.28 |
| 1-Chlorooctane | 4.95 | 0.19 | 0.40 | 0.00 | 0.10 | 1.36 | 4.77 |
| 1-Hexene | 3.51 | 0.08 | 0.08 | 0.00 | 0.07 | 0.91 | 2.57 |
| 1-Heptene | 4.04 | 0.09 | 0.08 | 0.00 | 0.07 | 1.05 | 3.06 |
| 1-Octene | 4.60 | 0.09 | 0.08 | 0.00 | 0.07 | 1.19 | 3.57 |
| 1-Nonene | 5.15 | 0.09 | 0.08 | 0.00 | 0.07 | 1.33 | 4.07 |
| 1-Decene | 5.64 | 0.09 | 0.08 | 0.00 | 0.07 | 1.47 | 4.57 |
| 1,2,4-Trichlorobenzene | 4.17 | 0.98 | 0.81 | 0.00 | 0.00 | 1.08 | 5.25 |

| | | | | | | | |
|---|---|---|---|---|---|---|---|
| Di-n-propyl ether | 2.06 | 0.01 | 0.25 | 0.00 | 0.45 | 1.01 | 2.95 |
| Dipentyl ether | 4.16 | 0.00 | 0.25 | 0.00 | 0.45 | 1.58 | 4.80 |
| 2-Octanone | 2.31 | 0.11 | 0.68 | 0.00 | 0.51 | 1.25 | 4.26 |
| 2-Nonanone | 2.79 | 0.12 | 0.68 | 0.00 | 0.51 | 1.39 | 4.74 |
| 1-Nitrobutane | 1.53 | 0.23 | 0.95 | 0.00 | 0.29 | 0.85 | 3.42 |
| 1-Nitrohexane | 2.58 | 0.20 | 0.95 | 0.00 | 0.29 | 1.13 | 4.42 |
| 4-Ethylpyridine | 1.14 | 0.63 | 0.80 | 0.00 | 0.57 | 0.96 | 4.12 |
| 1-Chloro-4-nitrobenzene | 2.38 | 0.98 | 1.18 | 0.00 | 0.24 | 1.01 | 5.22 |
| Nitrobenzene | 1.92 | 0.87 | 1.11 | 0.00 | 0.28 | 0.89 | 4.56 |
| 2-Nitrotoluene | 2.40 | 0.87 | 1.11 | 0.00 | 0.28 | 1.03 | 4.88 |
| 2,6-Dinitrotoluene | 1.94 | 1.15 | 1.60 | 0.00 | 0.45 | 1.21 | 6.30 |
| 4-Nitroanisole | 2.32 | 0.97 | 1.21 | 0.00 | 0.40 | 1.09 | 5.85 |
| 1,4-Dimethoxybenzene | 2.12 | 0.81 | 1.00 | 0.00 | 0.50 | 1.12 | 5.04 |
| 4-Chlorophenol | 1.51 | 0.92 | 1.08 | 0.67 | 0.20 | 0.90 | 4.78 |
| Ethyl benzoate | 2.59 | 0.69 | 0.85 | 0.00 | 0.46 | 1.21 | 5.20 |
| 2-Ethyl-1-hexanol | 2.04 | 0.21 | 0.39 | 0.37 | 0.48 | 1.29 | 4.38 |
| 3-Ethyl-3-hexanol | 1.70 | 0.20 | 0.30 | 0.31 | 0.60 | 1.29 | 4.29 |
| 4-Ethyl-3-hexanol | 1.92 | 0.17 | 0.36 | 0.33 | 0.57 | 1.29 | 4.18 |
| 3-Ethyl-3-pentanol | 1.13 | 0.20 | 0.30 | 0.31 | 0.60 | 1.15 | 3.79 |
| 2,4-Dinitrotoluene | 2.34 | 1.15 | 1.60 | 0.00 | 0.47 | 1.21 | 6.26 |
| 2-(Chlorodifluoromethoxy)-1,1,1-trifluoroethane | 2.73 | -0.24 | 0.14 | 0.00 | 0.06 | 0.90 | 1.58 |
| 1-Fluropropane | 0.98 | 0.03 | 0.35 | 0.00 | 0.13 | 0.69 | 1.10 |
| Hexachloroethane | 3.71 | 0.68 | 0.68 | 0.00 | 0.00 | 1.12 | 4.72 |
| Biphenyl | 4.14 | 1.36 | 0.99 | 0.00 | 0.26 | 1.32 | 6.01 |
| 1,1,1,2,3,4,4,4-Octafluorobutane | 2.44 | -0.79 | 0.20 | 0.13 | 0.05 | 0.97 | 1.10 |
| Hexadecane | 9.88 | 0.00 | 0.00 | 0.00 | 0.00 | 2.36 | 7.71 |
| Isopropyl bromide | 2.28 | 0.33 | 0.35 | 0.00 | 0.14 | 0.71 | 2.39 |
| 1,1,1,2,2,3,3,4,4-Nonafluorobutane | 2.82 | -0.78 | -0.30 | 0.10 | 0.10 | 1.01 | 0.42 |
| beta-Pinene | 4.11 | 0.53 | 0.24 | 0.00 | 0.19 | 1.26 | 4.39 |
| Limonene | 4.17 | 0.49 | 0.28 | 0.00 | 0.21 | 1.32 | 4.73 |
| Fluoromethane | 0.00 | 0.07 | 0.35 | 0.00 | 0.09 | 0.29 | 0.06 |
| Tricyclo[5.2.1.0(2,6)]decane | 4.62 | 0.59 | 0.45 | 0.00 | 0.06 | 1.19 | 4.84 |
| Methyl tert-butyl ether | 0.89 | 0.02 | 0.21 | 0.00 | 0.59 | 0.87 | 2.38 |
| Sulfur hexafluoride | 1.85 | -0.60 | -0.20 | 0.00 | 0.00 | 0.58 | -0.12 |
| Vinyl chloride | 1.58 | 0.26 | 0.38 | 0.00 | 0.05 | 0.47 | 1.40 |
| Dimethyl sulfoxide | -2.66 | 0.52 | 1.72 | 0.00 | 0.97 | 0.61 | 3.46 |
| Formic acid | -1.69 | 0.34 | 0.75 | 0.76 | 0.33 | 0.32 | 1.55 |
| 3-Carene | 4.71 | 0.51 | 0.22 | 0.00 | 0.10 | 1.26 | 4.65 |
| Vinyl bromide | 1.34 | 0.56 | 0.50 | 0.00 | 0.07 | 0.52 | 1.85 |
| Allyl chloride | 1.84 | 0.33 | 0.56 | 0.00 | 0.05 | 0.61 | 2.11 |
| Styrene | 2.68 | 0.85 | 0.65 | 0.00 | 0.16 | 0.96 | 3.86 |
| Octane | 5.27 | 0.00 | 0.00 | 0.00 | 0.00 | 1.24 | 3.68 |
| 3-Chlorophenol | 1.66 | 0.91 | 1.06 | 0.69 | 0.15 | 0.90 | 4.77 |
| Benzyl acetate | 1.70 | 0.80 | 1.06 | 0.00 | 0.65 | 1.21 | 5.01 |
| 1-Naphthol | 2.19 | 1.52 | 1.05 | 0.61 | 0.37 | 1.14 | 6.13 |
| 4-Bromophenol | 1.61 | 1.08 | 1.17 | 0.67 | 0.20 | 0.95 | 5.14 |
| N,N-Diethylaniline | 3.17 | 0.95 | 0.80 | 0.00 | 0.50 | 1.38 | 5.29 |
| 4-n-Propylphenol | 2.19 | 0.79 | 0.88 | 0.55 | 0.37 | 1.20 | 5.19 |
| 1,3-Dinitrobenzene | 1.42 | 1.15 | 1.60 | 0.00 | 0.47 | 1.06 | 5.90 |
| Anthracene | 4.83 | 2.29 | 1.34 | 0.00 | 0.28 | 1.45 | 7.57 |
| Phenanthrene | 4.80 | 2.06 | 1.29 | 0.00 | 0.29 | 1.45 | 7.63 |
| Fluoranthene | 5.18 | 2.38 | 1.55 | 0.00 | 0.20 | 1.58 | 8.83 |
| Pyrene | 5.26 | 2.81 | 1.71 | 0.00 | 0.29 | 1.58 | 8.83 |
| Fluorene | 4.39 | 1.59 | 1.03 | 0.00 | 0.20 | 1.36 | 6.92 |
| Acenaphthene | 3.97 | 1.60 | 1.04 | 0.00 | 0.20 | 1.26 | 6.47 |

Table S4. Data and descriptors for log $K_{oc}$.[8]

| | Log $K_{oc}$ | *E* | *S* | *A* | *B* | *V* | *L* |
|---|---|---|---|---|---|---|---|
| Naphthalene | 2.48 | 1.34 | 0.92 | 0.00 | 0.20 | 1.09 | 5.16 |
| 1,2-Dimethylnaphthalene | 3.64 | 1.43 | 0.92 | 0.00 | 0.20 | 1.37 | 6.44 |
| Acenaphthene | 3.22 | 1.60 | 1.04 | 0.00 | 0.20 | 1.26 | 6.47 |
| Fluorene | 3.63 | 1.59 | 1.03 | 0.00 | 0.20 | 1.36 | 6.92 |
| Phenanthrene | 4.26 | 2.06 | 1.29 | 0.00 | 0.29 | 1.45 | 7.63 |
| Cyclohexene | 1.84 | 0.40 | 0.20 | 0.00 | 0.10 | 0.80 | 3.02 |
| 1-Methylcyclohexene | 2.08 | 0.39 | 0.20 | 0.00 | 0.10 | 0.94 | 3.48 |
| Chlorobenzene | 2.08 | 0.72 | 0.65 | 0.00 | 0.07 | 0.84 | 3.66 |
| 1,4-Dichlorobenzene | 2.88 | 0.83 | 0.75 | 0.00 | 0.02 | 0.96 | 4.44 |
| 1,2,4-Trichlorobenzene | 2.95 | 0.98 | 0.81 | 0.00 | 0.00 | 1.08 | 5.25 |
| 1,2,3,4-Tetrachlorobenzene | 3.51 | 1.18 | 0.92 | 0.00 | 0.00 | 1.21 | 6.17 |
| Propiophenone | 1.99 | 0.80 | 0.95 | 0.00 | 0.51 | 1.15 | 4.97 |
| Pentanophenone | 2.32 | 0.80 | 0.95 | 0.00 | 0.50 | 1.44 | 5.90 |
| Heptanophenone | 3.16 | 0.72 | 0.95 | 0.00 | 0.50 | 1.72 | 6.88 |
| 2-Nitroanisole | 1.89 | 0.73 | 1.47 | 0.22 | 0.30 | 1.09 | 5.60 |
| 3-Nitroanisole | 2.11 | 0.87 | 1.20 | 0.14 | 0.25 | 1.09 | 5.59 |
| 4-Nitroanisole | 2.28 | 0.97 | 1.21 | 0.00 | 0.24 | 1.09 | 5.85 |
| 2-Chloronitrobenzene | 2.08 | 1.02 | 1.25 | 0.08 | 0.24 | 1.01 | 5.24 |
| 1-Nitrooctane | 2.47 | 0.19 | 0.95 | 0.00 | 0.29 | 1.41 | 5.43 |
| Nitrobenzene | 1.90 | 0.87 | 1.11 | 0.00 | 0.28 | 0.89 | 4.56 |
| 2-Nitrotoluene | 1.82 | 0.87 | 1.11 | 0.00 | 0.28 | 1.03 | 4.88 |
| 2,4-Dinitrotoluene | 2.23 | 1.18 | 1.27 | 0.07 | 0.51 | 1.21 | 6.27 |
| 2,4,6-Trinitrotoluene | 1.92 | 1.40 | 1.70 | 0.13 | 0.59 | 1.38 | 7.03 |
| Anisole | 1.46 | 0.71 | 0.75 | 0.00 | 0.29 | 0.92 | 3.89 |
| Benzofuran | 2.04 | 0.89 | 0.83 | 0.00 | 0.15 | 0.91 | 4.36 |
| 2-Methylbenzofuran | 2.37 | 0.95 | 0.83 | 0.00 | 0.15 | 1.05 | 4.68 |
| Dibenzofuran | 3.51 | 1.41 | 1.02 | 0.00 | 0.17 | 1.27 | 6.72 |
| Thiophene | 1.33 | 0.69 | 0.56 | 0.00 | 0.15 | 0.64 | 2.82 |
| Methyl benzoate | 1.55 | 0.73 | 0.85 | 0.00 | 0.46 | 1.07 | 4.70 |
| Ethyl benzoate | 1.92 | 0.69 | 0.85 | 0.00 | 0.46 | 1.21 | 5.20 |
| Diethyl phthalate | 1.53 | 0.73 | 1.40 | 0.00 | 0.88 | 1.71 | 6.79 |
| Di-n-propyl phthalate | 2.01 | 0.71 | 1.40 | 0.00 | 0.88 | 1.99 | 7.70 |
| Di-n-butyl phthalate | 3.20 | 0.67 | 1.40 | 0.00 | 0.88 | 2.27 | 8.60 |
| Di-n-pentyl phthalate | 3.73 | 0.68 | 1.40 | 0.00 | 0.88 | 2.56 | 9.52 |
| 1,2-Dicyanobenzene | 1.69 | 0.71 | 1.95 | 0.00 | 0.27 | 1.29 | 5.64 |
| 1-Naphthol | 2.36 | 1.52 | 1.05 | 0.61 | 0.37 | 1.14 | 6.13 |
| 2-Chlorophenol | 1.85 | 0.85 | 0.88 | 0.32 | 0.31 | 0.90 | 4.18 |
| 4-Chlorophenol | 1.94 | 0.92 | 1.08 | 0.67 | 0.20 | 0.90 | 4.78 |
| 3,4-Dichlorophenol | 2.40 | 1.02 | 1.14 | 0.85 | 0.03 | 1.02 | 5.71 |
| 2,4,5-Trichlorophenol | 2.90 | 1.07 | 0.92 | 0.73 | 0.10 | 1.14 | 5.73 |
| 2-Chloroaniline | 1.59 | 1.03 | 0.92 | 0.25 | 0.31 | 0.94 | 4.67 |
| 2,6-Dimethylaniline | 1.38 | 0.97 | 0.89 | 0.20 | 0.46 | 1.10 | 5.03 |
| Indole | 2.04 | 1.20 | 1.12 | 0.44 | 0.22 | 0.95 | 5.51 |
| Estradiol | 2.76 | 1.80 | 1.77 | 0.86 | 1.10 | 2.20 | 11.11 |
| Ibuprofen | 2.63 | 0.70 | 0.92 | 0.60 | 0.60 | 1.78 | 6.90 |
| Bisphenol A | 2.49 | 1.61 | 1.56 | 0.99 | 0.91 | 1.86 | 8.95 |
| 1-Chloropentane | 2.47 | 0.21 | 0.40 | 0.00 | 0.10 | 0.94 | 3.22 |
| 1-Chloroheptane | 3.36 | 0.19 | 0.40 | 0.00 | 0.10 | 1.22 | 4.28 |
| 1-Chlorooctane | 3.83 | 0.19 | 0.40 | 0.00 | 0.10 | 1.36 | 4.77 |
| Di-n-butyl ether | 1.65 | 0.00 | 0.25 | 0.00 | 0.45 | 1.29 | 3.92 |
| Di-n-pentyl ether | 2.78 | 0.00 | 0.25 | 0.00 | 0.45 | 1.58 | 4.80 |
| Di-n-hexyl ether | 3.60 | 0.00 | 0.25 | 0.00 | 0.45 | 1.86 | 5.74 |
| 1-Heptene | 2.84 | 0.09 | 0.08 | 0.00 | 0.07 | 1.05 | 3.06 |
| 1-Octene | 3.33 | 0.09 | 0.08 | 0.00 | 0.07 | 1.19 | 3.57 |

| | | | | | | | |
|---|---|---|---|---|---|---|---|
| 1-Nonene | 3.84 | 0.09 | 0.08 | 0.00 | 0.07 | 1.33 | 4.07 |
| 1-Decene | 4.39 | 0.09 | 0.08 | 0.00 | 0.07 | 1.47 | 4.58 |
| 2-Octanone | 1.24 | 0.11 | 0.68 | 0.00 | 0.51 | 1.25 | 4.26 |
| 2-Nonanone | 1.87 | 0.12 | 0.68 | 0.00 | 0.51 | 1.39 | 4.74 |
| 2-Decanone | 2.37 | 0.11 | 0.68 | 0.00 | 0.51 | 1.53 | 5.25 |
| 2-Undecanone | 2.82 | 0.10 | 0.68 | 0.00 | 0.51 | 1.67 | 5.73 |
| 1-Nitropentane | 1.40 | 0.21 | 0.95 | 0.00 | 0.29 | 0.99 | 3.94 |
| 1-Nitrohexane | 1.95 | 0.20 | 0.95 | 0.00 | 0.29 | 1.13 | 4.42 |
| Ethyl tert-butyl ether | 0.64 | -0.02 | 0.16 | 0.00 | 0.60 | 1.01 | 2.72 |
| 2,2,4-Trimethylpentane | 3.60 | 0.00 | 0.00 | 0.00 | 0.00 | 1.24 | 3.11 |
| Ethyl tert-pentyl ether | 0.99 | 0.00 | 0.16 | 0.00 | 0.61 | 1.15 | 3.26 |
| 3-Ethyl-3-hexanol | 1.05 | 0.20 | 0.30 | 0.31 | 0.60 | 1.29 | 4.29 |
| 4-Ethyl-3-hexanol | 1.28 | 0.17 | 0.36 | 0.33 | 0.57 | 1.29 | 4.18 |
| 2-Ethyl-1-hexanol | 1.51 | 0.21 | 0.39 | 0.37 | 0.48 | 1.29 | 4.38 |
| Isoflurane | 1.26 | -0.24 | 0.56 | 0.00 | 0.08 | 0.90 | 1.97 |
| Enflurane | 1.19 | -0.24 | 0.54 | 0.01 | 0.10 | 0.90 | 2.01 |
| Halothane | 1.46 | 0.10 | 0.39 | 0.13 | 0.05 | 0.80 | 1.95 |
| Methoxyflurane | 1.44 | 0.11 | 0.67 | 0.07 | 0.14 | 0.91 | 2.86 |
| Benzene | 1.32 | 0.61 | 0.52 | 0.00 | 0.14 | 0.72 | 2.79 |
| Toluene | 1.77 | 0.60 | 0.52 | 0.00 | 0.14 | 0.86 | 3.33 |
| Ethylbenzene | 2.08 | 0.61 | 0.51 | 0.00 | 0.15 | 1.00 | 3.78 |
| Propylbenzene | 2.53 | 0.60 | 0.50 | 0.00 | 0.15 | 1.14 | 4.23 |
| Butylbenzene | 2.99 | 0.60 | 0.51 | 0.00 | 0.15 | 1.28 | 4.73 |
| Pentylbenzene | 3.51 | 0.59 | 0.51 | 0.00 | 0.15 | 1.42 | 5.23 |
| Hexylbenzene | 3.93 | 0.59 | 0.50 | 0.00 | 0.15 | 1.56 | 5.72 |

Table S5. Data and descriptors for log $K_{lipw}$.[9]

| | Log $K_{lipw}$ | *E* | *S* | *A* | *B* | *V* | *L* |
|---|---|---|---|---|---|---|---|
| n-hexane | 3.91 | 0.00 | 0.00 | 0.00 | 0.00 | 0.9540 | 2.668 |
| n-heptane | 4.55 | 0.00 | 0.00 | 0.00 | 0.00 | 1.0949 | 3.173 |
| n-octane | 4.68 | 0.00 | 0.00 | 0.00 | 0.00 | 1.2358 | 3.677 |
| 2,2,4-trimethylpentane | 4.61 | 0.00 | 0.00 | 0.00 | 0.00 | 1.2358 | 3.106 |
| cyclohexane | 3.27 | 0.31 | 0.10 | 0.00 | 0.00 | 0.8454 | 2.964 |
| tetrachloromethane | 2.61 | 0.46 | 0.38 | 0.00 | 0.00 | 0.7391 | 2.823 |
| trichloroethene | 2.43 | 0.52 | 0.37 | 0.08 | 0.03 | 0.7146 | 2.997 |
| tetrachloroethene | 3.08 | 0.64 | 0.44 | 0.00 | 0.00 | 0.8370 | 3.584 |
| tribromomethane | 2.33 | 0.97 | 0.68 | 0.15 | 0.06 | 0.7745 | 3.784 |
| di-n-butyl ether | 2.78 | 0.00 | 0.25 | 0.00 | 0.45 | 1.2945 | 3.924 |
| di-n-pentyl ether | 3.77 | 0.00 | 0.25 | 0.00 | 0.45 | 1.5763 | 4.875 |
| acetone | 0.06 | 0.18 | 0.70 | 0.04 | 0.49 | 0.5470 | 1.696 |
| 2-octanone | 2.42 | 0.11 | 0.68 | 0.00 | 0.51 | 1.2515 | 4.257 |
| 2-nonanone | 2.84 | 0.12 | 0.68 | 0.00 | 0.51 | 1.3924 | 4.735 |
| 2-decanone | 3.16 | 0.11 | 0.68 | 0.00 | 0.51 | 1.5333 | 5.245 |
| cyclopentanone | 0.30 | 0.37 | 0.86 | 0.00 | 0.52 | 0.7200 | 3.220 |
| cyclohexanone | 0.54 | 0.40 | 0.86 | 0.00 | 0.56 | 0.8600 | 3.790 |
| ethyl acetate | 0.46 | 0.11 | 0.62 | 0.00 | 0.45 | 0.7466 | 2.314 |
| propyl acetate | 1.01 | 0.09 | 0.60 | 0.00 | 0.45 | 0.8875 | 2.819 |
| methanol | -0.53 | 0.28 | 0.44 | 0.43 | 0.47 | 0.3082 | 0.970 |
| ethanol | -0.26 | 0.25 | 0.42 | 0.37 | 0.48 | 0.4491 | 1.485 |
| 1-propanol | 0.17 | 0.24 | 0.42 | 0.37 | 0.48 | 0.5900 | 2.031 |
| 2-propanol | -0.04 | 0.21 | 0.36 | 0.33 | 0.56 | 0.5900 | 1.764 |
| 1-butanol | 0.51 | 0.22 | 0.42 | 0.37 | 0.48 | 0.7309 | 2.601 |
| tert-butanol | 0.16 | 0.28 | 0.30 | 0.31 | 0.60 | 0.7309 | 1.963 |
| 1-pentanol | 1.08 | 0.22 | 0.42 | 0.37 | 0.48 | 0.8700 | 3.110 |
| 3-pentanol | 1.00 | 0.22 | 0.36 | 0.33 | 0.56 | 0.8718 | 2.860 |

| | | | | | | | |
|---|---|---|---|---|---|---|---|
| 1-hexanol | 1.88 | 0.21 | 0.42 | 0.37 | 0.48 | 1.0127 | 3.610 |
| 1-heptanol | 2.38 | 0.21 | 0.42 | 0.37 | 0.48 | 1.1500 | 4.120 |
| 4-heptanol | 1.70 | 0.18 | 0.36 | 0.33 | 0.56 | 1.1500 | 3.850 |
| 1-octanol | 2.66 | 0.20 | 0.42 | 0.37 | 0.48 | 1.2900 | 4.620 |
| cyclopentanol | 0.52 | 0.43 | 0.54 | 0.32 | 0.56 | 0.7600 | 3.240 |
| cyclohexanol | 1.01 | 0.46 | 0.54 | 0.32 | 0.57 | 0.9041 | 3.758 |
| cycloheptanol | 1.51 | 0.51 | 0.54 | 0.32 | 0.58 | 1.0500 | 4.410 |
| ethylene glycol | -0.79 | 0.40 | 0.90 | 0.58 | 0.78 | 0.5078 | 2.661 |
| 2-butoxyethanol | 0.60 | 0.20 | 0.53 | 0.26 | 0.83 | 1.0714 | 3.656 |
| benzyl alcohol | 1.14 | 0.80 | 0.87 | 0.33 | 0.56 | 0.9160 | 4.221 |
| p-xylene | 2.98 | 0.61 | 0.52 | 0.00 | 0.16 | 0.9982 | 3.839 |
| chlorobenzene | 2.91 | 0.72 | 0.65 | 0.00 | 0.07 | 0.8388 | 3.657 |
| 1,2-dichlorobenzene | 3.64 | 0.87 | 0.78 | 0.00 | 0.04 | 0.9612 | 4.518 |
| 1,3-dichlorobenzene | 3.71 | 0.85 | 0.73 | 0.00 | 0.02 | 0.9612 | 4.410 |
| 1,4-dichlorobenzene | 3.57 | 0.83 | 0.75 | 0.00 | 0.02 | 0.9612 | 4.435 |
| 1,2,3-trichlorobenzene | 4.19 | 1.03 | 0.86 | 0.00 | 0.00 | 1.0836 | 5.419 |
| 1,2,4-trichlorobenzene | 4.20 | 0.98 | 0.81 | 0.00 | 0.00 | 1.0836 | 5.248 |
| 1,3,5-trichlorobenzene | 4.16 | 0.98 | 0.73 | 0.00 | 0.00 | 1.0836 | 5.045 |
| 1,2,3,5-tetrachlorobenzene | 4.77 | 1.16 | 0.85 | 0.00 | 0.00 | 1.2060 | 5.922 |
| 1,2,4,5-tetrachlorobenzene | 4.73 | 1.16 | 0.86 | 0.00 | 0.00 | 1.2060 | 5.926 |
| pentachlorobenzene | 5.18 | 1.33 | 0.92 | 0.06 | 0.00 | 1.3284 | 6.630 |
| hexachlorobenzene | 5.64 | 1.49 | 0.99 | 0.00 | 0.00 | 1.4508 | 7.390 |
| 2,4,5-trichlorotoluene | 4.72 | 1.06 | 0.85 | 0.00 | 0.00 | 1.2245 | 5.805 |
| 1,4-dibromobenzene | 4.30 | 1.15 | 0.86 | 0.00 | 0.04 | 1.0664 | 5.324 |
| 2,2',4,6-tetrachlorobiphenyl (PCB 50) | 5.92 | 1.86 | 1.48 | 0.00 | 0.15 | 1.8138 | 7.854 |
| 2,2',5,5'-tetrachlorobiphenyl (PCB 52) | 5.94 | 1.90 | 1.48 | 0.00 | 0.15 | 1.8138 | 8.144 |
| 3,3',4,5-tetrachlorobiphenyl (PCB 78) | 6.54 | 1.94 | 1.44 | 0.00 | 0.11 | 1.8138 | 9.039 |
| 2,2',4,5',6-pentachlorobiphenyl (PCB 103) | 6.32 | 2.01 | 1.61 | 0.00 | 0.13 | 1.9362 | 8.429 |
| 2,2',4,6,6'-pentachlorobiphenyl (PCB 104) | 6.13 | 1.98 | 1.61 | 0.00 | 0.13 | 1.9362 | 8.244 |
| 2,2',3,3',6,6'-he'achlorobiphenyl (PCB 136) | 6.50 | 2.14 | 1.74 | 0.00 | 0.11 | 2.0586 | 9.117 |
| 2,2',3,4,4',5,6'-heptachlorobiphenyl (PCB 182) | 6.83 | 2.30 | 1.87 | 0.00 | 0.09 | 2.1810 | 9.958 |
| phenanthrene | 4.95 | 2.06 | 1.29 | 0.00 | 0.29 | 1.4544 | 7.632 |
| anthracene | 5.21 | 2.29 | 1.34 | 0.00 | 0.28 | 1.4544 | 7.568 |
| fluoranthene | 5.58 | 2.38 | 1.55 | 0.00 | 0.24 | 1.5846 | 8.827 |
| pyrene | 5.71 | 2.81 | 1.71 | 0.00 | 0.28 | 1.5846 | 8.833 |
| benzo[a]anthracene | 6.44 | 2.99 | 1.70 | 0.00 | 0.33 | 1.8234 | 10.291 |
| chrysene | 6.40 | 3.03 | 1.73 | 0.00 | 0.33 | 1.8234 | 10.334 |
| benzo[b]fluoranthene | 7.11 | 3.19 | 1.82 | 0.00 | 0.40 | 1.9536 | 11.632 |
| benzo[k]fluoranthene | 7.13 | 3.19 | 1.91 | 0.00 | 0.33 | 1.9536 | 11.607 |
| benzo[a]pyrene | 7.19 | 3.63 | 1.96 | 0.00 | 0.37 | 1.9536 | 11.736 |
| benzo[ghi]perylene | 7.78 | 4.07 | 1.90 | 0.00 | 0.45 | 2.0838 | 13.264 |
| dibenz[a,h]anthracene | 7.72 | 4.00 | 2.04 | 0.00 | 0.44 | 2.1924 | 12.960 |
| dibenz[a,c]anthracene | 7.49 | 4.00 | 1.93 | 0.00 | 0.44 | 2.1924 | 12.998 |
| indeno[1,2,3-cd]pyrene | 7.86 | 3.61 | 1.93 | 0.00 | 0.42 | 2.0838 | 12.699 |
| nitrobenzene | 2.01 | 0.87 | 1.11 | 0.00 | 0.28 | 0.8906 | 4.557 |
| 2-nitrotoluene | 2.41 | 0.87 | 1.11 | 0.00 | 0.28 | 1.0315 | 4.878 |
| quinoline | 1.67 | 1.27 | 0.97 | 0.00 | 0.54 | 1.0443 | 5.457 |
| diethylphthalate | 1.77 | 0.73 | 1.40 | 0.00 | 0.88 | 1.7106 | 6.787 |
| dibutylphthalate | 3.87 | 0.70 | 1.40 | 0.00 | 0.86 | 2.2742 | 8.605 |
| phenol | 1.96 | 0.81 | 0.89 | 0.60 | 0.30 | 0.7751 | 3.766 |
| 2-methylphenol | 2.45 | 0.84 | 0.86 | 0.52 | 0.30 | 0.9160 | 4.218 |
| 3-methylphenol | 2.34 | 0.82 | 0.88 | 0.57 | 0.34 | 0.9160 | 4.310 |
| 4-methylphenol | 2.35 | 0.82 | 0.87 | 0.57 | 0.31 | 0.9160 | 4.312 |
| 2-ethylphenol | 2.81 | 0.83 | 0.84 | 0.52 | 0.37 | 1.0569 | 4.612 |
| 4-ethylphenol | 2.78 | 0.80 | 0.90 | 0.55 | 0.36 | 1.0569 | 4.737 |
| 2,6-dimethylphenol | 2.47 | 0.84 | 0.79 | 0.39 | 0.38 | 1.0569 | 4.680 |
| 2-n-propylphenol | 3.13 | 0.82 | 0.86 | 0.52 | 0.37 | 1.1978 | 4.951 |

| | | | | | | | |
|---|---|---|---|---|---|---|---|
| 4-n-propylphenol | 2.92 | 0.79 | 0.88 | 0.55 | 0.37 | 1.1978 | 5.185 |
| 4-isopropylphenol | 3.25 | 0.79 | 0.89 | 0.55 | 0.38 | 1.2000 | 4.980 |
| 3,4,5-trimethylphenol | 2.66 | 0.83 | 0.89 | 0.58 | 0.42 | 1.1978 | 5.568 |
| 4-n-butylphenol | 3.13 | 0.80 | 0.88 | 0.55 | 0.37 | 1.3400 | 5.640 |
| 2-sec-butylphenol | 3.47 | 0.82 | 0.91 | 0.52 | 0.41 | 1.3387 | 5.050 |
| 2-tert-butylphenol | 3.51 | 0.82 | 0.92 | 0.52 | 0.40 | 1.3387 | 5.021 |
| 4-tert-butylphenol | 3.48 | 0.81 | 0.91 | 0.56 | 0.40 | 1.3387 | 5.264 |
| 4-tert-amylphenol | 3.54 | 0.81 | 0.89 | 0.56 | 0.41 | 1.4796 | 5.775 |
| 2-phenylphenol | 3.43 | 1.55 | 1.40 | 0.56 | 0.49 | 1.3829 | 7.227 |
| 4-phenylphenol | 3.52 | 1.56 | 1.41 | 0.59 | 0.45 | 1.3830 | 7.052 |
| bisphenol A | 3.92 | 1.61 | 1.56 | 0.99 | 0.91 | 1.8643 | 9.603 |
| 2-chlorophenol | 2.76 | 0.85 | 0.88 | 0.32 | 0.31 | 0.8975 | 4.178 |
| 3-chlorophenol | 2.78 | 0.91 | 1.06 | 0.69 | 0.15 | 0.8975 | 4.773 |
| 4-chlorophenol | 2.73 | 0.92 | 1.08 | 0.67 | 0.20 | 0.8975 | 4.775 |
| 4-chloro-3-methylphenol | 3.32 | 0.92 | 1.02 | 0.67 | 0.22 | 1.0384 | 5.290 |
| 2,4-dichlorophenol | 3.57 | 0.96 | 0.82 | 0.54 | 0.17 | 1.0199 | 4.896 |
| 2,6-dichlorophenol | 2.86 | 0.90 | 0.86 | 0.36 | 0.24 | 1.0199 | 5.086 |
| 3,4-dichlorophenol | 3.76 | 1.02 | 1.24 | 0.93 | 0.00 | 1.0199 | 5.708 |
| 2,4,5-trichlorophenol | 4.46 | 1.07 | 0.92 | 0.73 | 0.10 | 1.1423 | 5.725 |
| 2,4,6-trichlorophenol | 3.80 | 1.01 | 0.80 | 0.68 | 0.15 | 1.1423 | 5.664 |
| 3,4,5-trichlorophenol | 4.71 | 1.13 | 0.92 | 0.99 | 0.00 | 1.1423 | 6.351 |
| 2,3,4,5-tetrachlorophenol | 4.76 | 1.17 | 0.88 | 0.70 | 0.13 | 1.2647 | 6.353 |
| pentachlorophenol | 5.10 | 1.22 | 0.86 | 0.61 | 0.09 | 1.3871 | 7.489 |
| 4-fluorophenol | 2.19 | 0.67 | 0.97 | 0.63 | 0.23 | 0.8100 | 3.840 |
| 4-bromophenol | 2.40 | 1.08 | 1.17 | 0.67 | 0.20 | 0.9500 | 5.140 |
| 4-iodophenol | 2.55 | 1.38 | 1.22 | 0.68 | 0.20 | 1.0300 | 5.490 |
| 2-nitrophenol | 1.89 | 1.02 | 1.05 | 0.05 | 0.37 | 0.9493 | 4.760 |
| 3-nitrophenol | 2.56 | 1.05 | 1.57 | 0.79 | 0.23 | 0.9493 | 5.692 |
| 4-nitrophenol | 2.72 | 1.07 | 1.72 | 0.82 | 0.26 | 0.9493 | 5.876 |
| 2,4-dinitrophenol | 2.67 | 1.20 | 1.49 | 0.09 | 0.56 | 1.1235 | 5.981 |
| 2,6-dinitrophenol | 2.03 | 1.22 | 2.04 | 0.17 | 0.48 | 1.1235 | 6.189 |
| 3,4-dinitrophenol | 3.17 | 1.32 | 2.25 | 1.14 | 0.16 | 1.1235 | 5.953 |
| 2-sec-butyl-4,6-dinitrophenol | 3.73 | 0.90 | 1.75 | 0.17 | 0.46 | 1.6871 | 7.755 |
| 4-cyanophenol | 2.11 | 0.94 | 1.63 | 0.80 | 0.29 | 0.9298 | 5.420 |
| aniline | 1.63 | 0.96 | 0.96 | 0.26 | 0.41 | 0.8162 | 3.934 |
| 3,4-dimethylaniline | 2.11 | 0.96 | 0.97 | 0.20 | 0.49 | 1.0980 | 5.089 |
| 3-nitroaniline | 2.17 | 1.20 | 1.71 | 0.40 | 0.35 | 0.9904 | 5.880 |
| N,N-dimethylaniline | 2.33 | 0.96 | 0.84 | 0.00 | 0.41 | 1.0980 | 4.701 |
| estrone | 3.60 | 1.73 | 2.05 | 0.50 | 1.08 | 2.1558 | 10.780 |
| 17beta-estradiol | 3.33 | 1.80 | 1.77 | 0.86 | 1.10 | 2.1988 | 11.107 |
| estriol | 1.96 | 1.97 | 1.74 | 1.06 | 1.63 | 2.2575 | 11.430 |
| progesterone | 3.28 | 1.45 | 3.29 | 0.00 | 1.14 | 2.6215 | 12.050 |
| diazepam | 2.99 | 2.08 | 1.55 | 0.00 | 1.28 | 2.0739 | 10.480 |
| lidocaine | 2.15 | 1.10 | 1.47 | 0.06 | 1.24 | 2.0589 | 8.448 |
| diclofenac | 4.45 | 1.81 | 1.85 | 0.55 | 0.77 | 2.0250 | 11.025 |
| ibuprofen | 3.80 | 0.73 | 0.70 | 0.56 | 0.79 | 1.7771 | 7.184 |
| salicylic acid | 2.55 | 0.90 | 0.85 | 0.73 | 0.37 | 0.9904 | 4.732 |

Table S6. Data and descriptors for log $K_{BSAw}$.[10]

| | Log $K_{lipw}$ | *E* | *S* | *A* | *B* | *V* | *L* |
|---|---|---|---|---|---|---|---|
| n-hexane | 3.09 | 0.00 | 0.00 | 0.00 | 0.00 | 0.9540 | 2.668 |
| n-heptane | 3.59 | 0.00 | 0.00 | 0.00 | 0.00 | 1.0949 | 3.173 |
| n-octane | 4.01 | 0.00 | 0.00 | 0.00 | 0.00 | 1.2358 | 3.677 |
| n-nonane | 4.45 | 0.00 | 0.00 | 0.00 | 0.00 | 1.3767 | 4.182 |
| cyclohexane | 2.01 | 0.31 | 0.10 | 0.00 | 0.00 | 0.8454 | 2.964 |

| | | | | | | | |
|---|---|---|---|---|---|---|---|
| cycloheptane | 2.52 | 0.35 | 0.10 | 0.00 | 0.00 | 0.9863 | 3.704 |
| cyclooctane | 2.98 | 0.41 | 0.10 | 0.00 | 0.00 | 1.1272 | 4.329 |
| 1-nonene | 4.22 | 0.09 | 0.08 | 0.00 | 0.07 | 1.3337 | 4.073 |
| hept-1-yne | 2.49 | 0.16 | 0.23 | 0.13 | 0.10 | 1.0089 | 3.000 |
| 1-chlorooctane | 3.85 | 0.19 | 0.40 | 0.00 | 0.09 | 1.3582 | 4.708 |
| tetrachloromethane | 1.77 | 0.46 | 0.38 | 0.00 | 0.00 | 0.7391 | 2.823 |
| trichloroethene | 1.88 | 0.52 | 0.37 | 0.08 | 0.03 | 0.7146 | 2.997 |
| tetrachloroethene | 2.40 | 0.64 | 0.44 | 0.00 | 0.00 | 0.8370 | 3.584 |
| tribromomethane | 1.95 | 0.97 | 0.68 | 0.15 | 0.06 | 0.7745 | 3.784 |
| g-HCH | 2.46 | 1.45 | 1.28 | 0.00 | 0.50 | 1.5798 | 7.467 |
| isoflurane | 1.58 | -0.24 | 0.56 | 0.00 | 0.08 | 0.9009 | 1.969 |
| enflurane | 1.59 | -0.24 | 0.40 | 0.07 | 0.13 | 0.9009 | 1.750 |
| halothane | 1.62 | 0.10 | 0.39 | 0.13 | 0.05 | 0.8009 | 1.982 |
| methoxyflurane | 1.77 | 0.11 | 0.67 | 0.07 | 0.14 | 0.9102 | 2.831 |
| di-n-butyl ether | 2.01 | 0.00 | 0.25 | 0.00 | 0.45 | 1.2945 | 3.924 |
| di-n-pentyl ether | 3.00 | 0.00 | 0.25 | 0.00 | 0.45 | 1.5763 | 4.875 |
| 2-octanone | 2.09 | 0.11 | 0.68 | 0.00 | 0.51 | 1.2515 | 4.257 |
| 2-nonanone | 2.48 | 0.12 | 0.68 | 0.00 | 0.51 | 1.3924 | 4.735 |
| 2-decanone | 2.88 | 0.11 | 0.68 | 0.00 | 0.51 | 1.5333 | 5.245 |
| 1-nitrooctane | 3.38 | 0.19 | 0.95 | 0.00 | 0.29 | 1.4100 | 5.430 |
| tri-n-butyl phosphate | 2.47 | -0.10 | 0.90 | 0.00 | 1.21 | 2.2390 | 7.522 |
| benzene | 1.58 | 0.61 | 0.52 | 0.00 | 0.14 | 0.7164 | 2.786 |
| toluene | 2.26 | 0.60 | 0.52 | 0.00 | 0.14 | 0.8573 | 3.325 |
| ethylbenzene | 2.70 | 0.61 | 0.51 | 0.00 | 0.15 | 0.9982 | 3.778 |
| n-propylbenzene | 2.95 | 0.60 | 0.50 | 0.00 | 0.15 | 1.1391 | 4.230 |
| styrene | 2.76 | 0.85 | 0.65 | 0.00 | 0.16 | 0.9552 | 3.856 |
| chlorobenzene | 2.32 | 0.72 | 0.65 | 0.00 | 0.07 | 0.8388 | 3.657 |
| 1,2-dichlorobenzene | 3.03 | 0.87 | 0.78 | 0.00 | 0.04 | 0.9612 | 4.518 |
| 1,2,4-trimethylbenzene | 3.35 | 0.68 | 0.56 | 0.00 | 0.19 | 1.1391 | 4.441 |
| 1,4-dibromobenzene | 3.97 | 1.15 | 0.86 | 0.00 | 0.04 | 1.0664 | 5.324 |
| 1,2,4-Trichlorobenzene | 3.60 | 0.98 | 0.81 | 0.00 | 0.00 | 1.0836 | 5.248 |
| 1,2,3,4-Tetrachlorobenzene | 4.21 | 1.18 | 0.92 | 0.00 | 0.00 | 1.2060 | 6.171 |
| hexafluorobenzene | 1.55 | 0.09 | 0.56 | 0.00 | 0.01 | 0.9426 | 2.345 |
| methylpentafluorobenzene | 2.32 | 0.16 | 0.59 | 0.00 | 0.01 | 1.0458 | 3.244 |
| indene | 2.92 | 1.00 | 0.77 | 0.00 | 0.20 | 0.9880 | 4.559 |
| naphthalene | 3.56 | 1.34 | 0.92 | 0.00 | 0.20 | 1.0854 | 5.161 |
| dibenzofuran | 3.79 | 1.41 | 1.02 | 0.00 | 0.17 | 1.2743 | 6.716 |
| dibenzothiophene | 4.16 | 1.96 | 1.31 | 0.00 | 0.20 | 1.3791 | 7.588 |
| phenanthrene | 4.15 | 2.06 | 1.29 | 0.00 | 0.29 | 1.4544 | 7.632 |
| fluoranthene | 4.28 | 2.38 | 1.55 | 0.00 | 0.24 | 1.5846 | 8.827 |
| pyrene | 4.76 | 2.81 | 1.71 | 0.00 | 0.28 | 1.5846 | 8.833 |
| chrysene | 4.46 | 3.03 | 1.73 | 0.00 | 0.33 | 1.8234 | 10.334 |
| benzo[b]fluoranthene | 4.42 | 3.19 | 1.82 | 0.00 | 0.40 | 1.9536 | 11.632 |
| benzo[ghi]perylene | 4.76 | 4.07 | 1.90 | 0.00 | 0.45 | 2.0838 | 13.447 |
| anisole | 2.16 | 0.71 | 0.75 | 0.00 | 0.29 | 0.9160 | 3.890 |
| valerophenone | 2.70 | 0.80 | 0.95 | 0.00 | 0.50 | 1.4366 | 5.900 |
| benzophenone | 2.62 | 1.45 | 1.50 | 0.00 | 0.50 | 1.4808 | 7.254 |
| di-n-propyl phthalate | 2.84 | 0.71 | 1.40 | 0.00 | 0.88 | 1.9924 | 8.271 |
| 2-nitrotoluene | 2.12 | 0.87 | 1.11 | 0.00 | 0.28 | 1.0315 | 4.878 |
| 2,4-dinitrotoluene | 1.73 | 1.17 | 1.27 | 0.07 | 0.51 | 1.2057 | 6.258 |
| 1-nitronaphthalene | 3.17 | 1.60 | 1.59 | 0.00 | 0.29 | 1.2596 | 7.056 |
| 4-nitroanisole | 2.48 | 0.97 | 1.29 | 0.00 | 0.40 | 1.0902 | 5.620 |
| N,N-diethylaniline | 2.27 | 0.95 | 0.80 | 0.00 | 0.50 | 1.3798 | 5.287 |
| 1-hexanol | 1.64 | 0.21 | 0.42 | 0.37 | 0.48 | 1.0127 | 3.610 |
| 1-heptanol | 2.18 | 0.21 | 0.42 | 0.37 | 0.48 | 1.1536 | 4.115 |
| 1-octanol | 2.74 | 0.20 | 0.42 | 0.37 | 0.48 | 1.2945 | 4.619 |
| 1-nonanol | 3.10 | 0.19 | 0.42 | 0.37 | 0.48 | 1.4354 | 5.120 |

| | | | | | | | |
|---|---|---|---|---|---|---|---|
| 4-ethyl-3-hexanol | 1.48 | 0.17 | 0.36 | 0.33 | 0.57 | 1.2945 | 4.177 |
| 4-chlorobenzyl alcohol | 2.10 | 0.91 | 0.96 | 0.40 | 0.50 | 1.0380 | 4.938 |
| 4-n-propylphenol | 2.59 | 0.79 | 0.88 | 0.55 | 0.37 | 1.1978 | 5.185 |
| 2-phenylphenol | 2.62 | 1.55 | 1.40 | 0.56 | 0.49 | 1.3829 | 7.227 |
| 4-fluorophenol | 1.57 | 0.67 | 0.97 | 0.63 | 0.23 | 0.8128 | 3.844 |
| 3-chlorophenol | 2.35 | 0.91 | 1.06 | 0.69 | 0.15 | 0.8975 | 4.773 |
| 4-chlorophenol | 2.43 | 0.92 | 1.08 | 0.67 | 0.20 | 0.8975 | 4.775 |
| 4-bromophenol | 2.81 | 1.08 | 1.17 | 0.67 | 0.20 | 0.9501 | 5.004 |
| 4-iodophenol | 3.41 | 1.38 | 1.22 | 0.68 | 0.20 | 1.0333 | 5.492 |
| bisphenol A | 2.88 | 1.61 | 1.56 | 0.99 | 0.91 | 1.8643 | 9.239 |
| 4-nitroaniline | 1.69 | 1.22 | 1.92 | 0.46 | 0.35 | 0.9904 | 6.042 |
| 2-chloroaniline | 1.95 | 1.03 | 0.92 | 0.25 | 0.31 | 0.9386 | 4.674 |
| 4-iodoaniline | 2.95 | 1.53 | 1.28 | 0.31 | 0.40 | 1.0744 | 5.695 |
| 4-aminobiphenyl | 2.55 | 1.57 | 1.48 | 0.26 | 0.48 | 1.4240 | 7.698 |
| indole | 2.25 | 1.20 | 1.12 | 0.44 | 0.22 | 0.9464 | 5.310 |
| carbazole | 3.52 | 1.79 | 1.42 | 0.47 | 0.26 | 1.3154 | 7.842 |
| metolachlor | 1.74 | 1.15 | 1.01 | 0.07 | 1.38 | 2.2811 | 9.350 |
| atrazine | 1.77 | 1.22 | 1.29 | 0.17 | 1.01 | 1.6196 | 7.783 |
| diazepam | 2.68 | 2.08 | 1.55 | 0.00 | 1.28 | 2.0739 | 11.010 |
| estrone | 2.69 | 1.73 | 2.05 | 0.50 | 1.08 | 2.1558 | 10.780 |

**Table S7. Ranges of partition coefficients and solute descriptors (min/max) considered in this study.**

| | *n* of compounds | Log *K* | Descriptors | | | | | | SD[a] | Ref |
|---|---|---|---|---|---|---|---|---|---|---|
| | | | *E* | *S* | *A* | *B* | *V* | *L* | | |
| Log $K_{ow}$ | 314 | -1.38/5.65 | -0.60/2.81 | -0.20/1.91 | 0.00/0.82 | 0.00/0.84 | 0.17/1.67 | -0.82/8.83 | 0.154 | 5 |
| Log $K_{aw}$ | 390 | -8.07/2.32 | -0.60/1.67 | -0.20/1.91 | 0.00/0.82 | 0.00/1.06 | 0.17/1.67 | -0.80/6.92 | 0.156 | 6 |
| Log $K_{oilw}$ | 247 | -2.66/9.88 | -0.79/2.81 | -0.30/1.72 | 0.00/0.76 | 0.00/0.97 | 0.25/2.36 | -0.82/8.83 | 0.286 | 7 |
| Log $K_{oc}$ | 79 | 0.64/4.39 | -0.24/2.06 | 0.00/1.95 | 0.00/0.99 | 0.00/1.10 | 0.64/2.56 | 1.95/11.11 | 0.250 | 8 |
| Log $K_{lipw}$ | 131 | -0.79/7.86 | 0.00/4.07 | 0.00/3.29 | 0.00/1.14 | 0.00/1.63 | 0.31/2.62 | 0.97/13.26 | 0.285 | 9 |
| Log $K_{BSAw}$ | 82 | 1.48/4.76 | -0.24/4.07 | 0.00/2.05 | 0.00/0.99 | 0.00/1.38 | 0.71/2.28 | 1.75/13.45 | 0.422 | 10 |

[a] Standard deviation of PP-LFER (eq 1) when all compounds are used for model training.

## SI-3 List of 25 applicability domain (AD) probes

**Table S8. Twenty-five applicability domain (AD) probes used to test the reported PP-LFERs.**

| | | | *E* | *S* | *A* | *B* | *V* | *L* |
|---|---|---|---|---|---|---|---|---|
| aliphatic | nonpolar | dichloromethane | 0.39 | 0.57 | 0.10 | 0.05 | 0.494 | 2.019 |
| | | hexachloroethane | 0.68 | 0.68 | 0.00 | 0.00 | 1.125 | 4.718 |
| | | *n*-hexadecane | 0.00 | 0.00 | 0.00 | 0.00 | 2.363 | 7.714 |
| | H-acceptor | methyl *tert*-butyl ether | 0.02 | 0.28 | 0.00 | 0.54 | 0.872 | 2.270 |
| | | molinate | 0.88 | 1.09 | 0.00 | 0.70 | 1.547 | 6.578 |
| | | tri-*n*-butyl phosphate | -0.10 | 0.90 | 0.00 | 1.21 | 2.239 | 7.370 |
| | H-donor | *tert*-butyl alcohol | 0.18 | 0.30 | 0.31 | 0.60 | 0.731 | 1.963 |
| | | decanoic acid | 0.12 | 0.64 | 0.62 | 0.45 | 1.592 | 5.698 |
| aromatic | nonpolar | benzene | 0.61 | 0.52 | 0.00 | 0.14 | 0.716 | 2.786 |
| | | hexachlorobenzene | 1.49 | 0.99 | 0.00 | 0.00 | 1.451 | 7.624 |
| | | phenanthrene | 2.06 | 1.29 | 0.00 | 0.29 | 1.454 | 7.632 |
| | | PCB 180 | 2.29 | 1.87 | 0.00 | 0.09 | 2.181 | 10.415 |
| | | benzo[*ghi*]perylene | 3.61 | 2.11 | 0.00 | 0.44 | 2.084 | 12.707 |
| | H-acceptor | nitrobenzene | 0.87 | 1.11 | 0.00 | 0.28 | 0.891 | 4.557 |
| | | benzophenone | 1.45 | 1.50 | 0.00 | 0.50 | 1.481 | 6.955 |
| | | di-*n*-butyl phthalate | 0.69 | 1.30 | 0.00 | 0.94 | 2.274 | 8.553 |
| | H-donor | phenol | 0.81 | 0.89 | 0.60 | 0.30 | 0.775 | 3.766 |
| | | pentachlorophenol | 1.22 | 0.87 | 0.96 | 0.01 | 1.387 | 6.822 |
| | | bisphenol A | 1.61 | 1.56 | 0.99 | 0.91 | 1.864 | 9.603 |
| multifunctional polar | | caffeine | 1.50 | 1.82 | 0.08 | 1.25 | 1.363 | 7.838 |
| | | metolachlor | 1.15 | 1.01 | 0.07 | 1.38 | 2.281 | 8.863 |
| | | diuron | 1.28 | 1.60 | 0.57 | 0.70 | 1.599 | 8.060 |
| | | estradiol | 1.80 | 1.77 | 0.86 | 1.10 | 2.199 | 11.107 |
| neutral PFAS | | 8:2 FTOH | -1.56 | 0.14 | 0.62 | 0.31 | 2.220 | 3.470 |
| organosilicon | | D5 | -0.70 | -0.10 | 0.00 | 0.50 | 2.931 | 5.242 |

8:2 FTOH, 1H,1H,2H,2H-perfluorodecan-1-ol; D5, decamethylcyclopentasiloxane.

## SI-4 RMSEs for training and test data (Test 1)

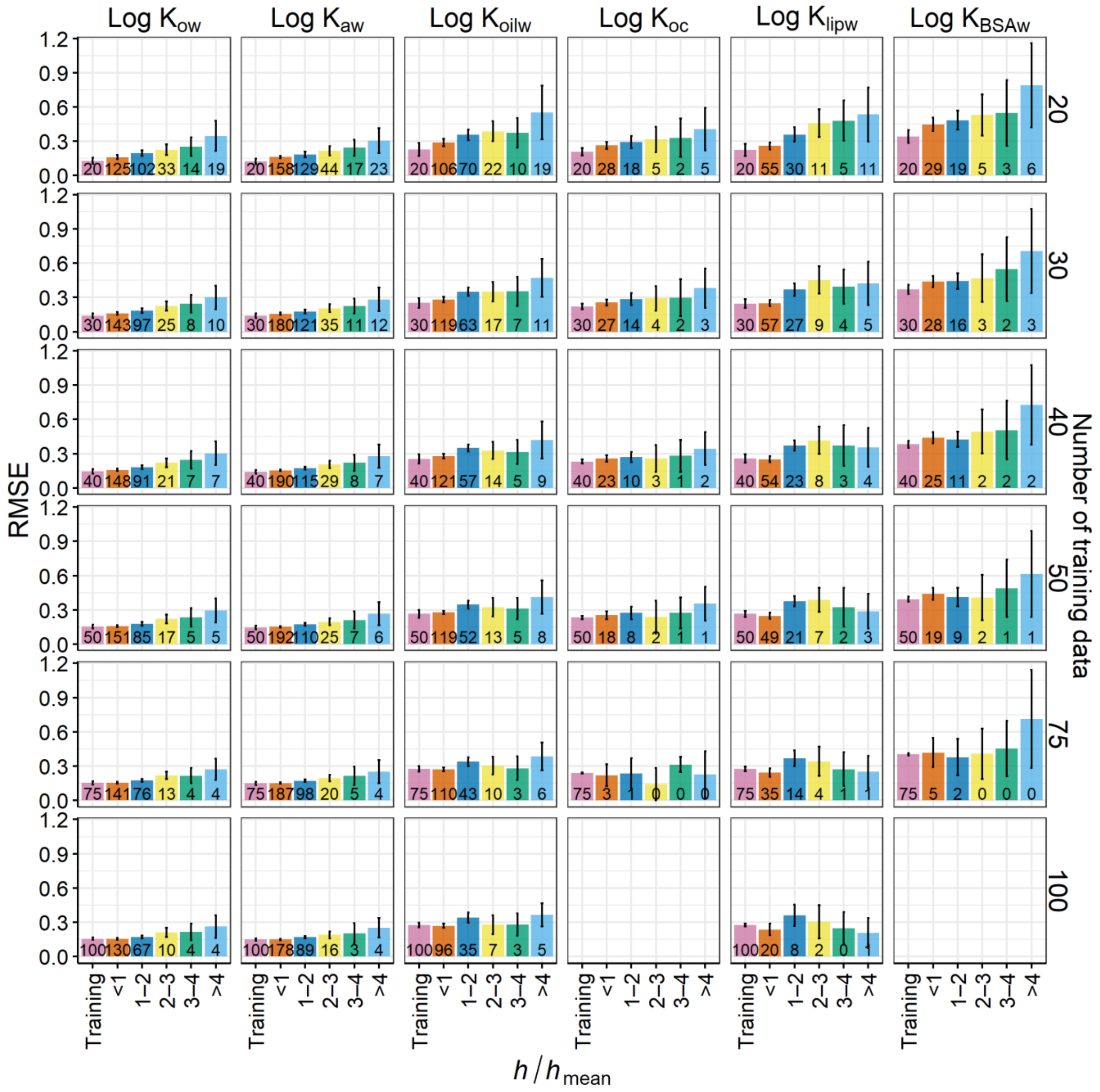


Figure S1. RMSEs for training and test data. Columns and error bars indicate the means and the standard deviations, respectively, for 200 repeated simulations. Test data were sorted into five bins according to their $h/h_{mean}$. Numbers in the columns indicate the mean number of data.

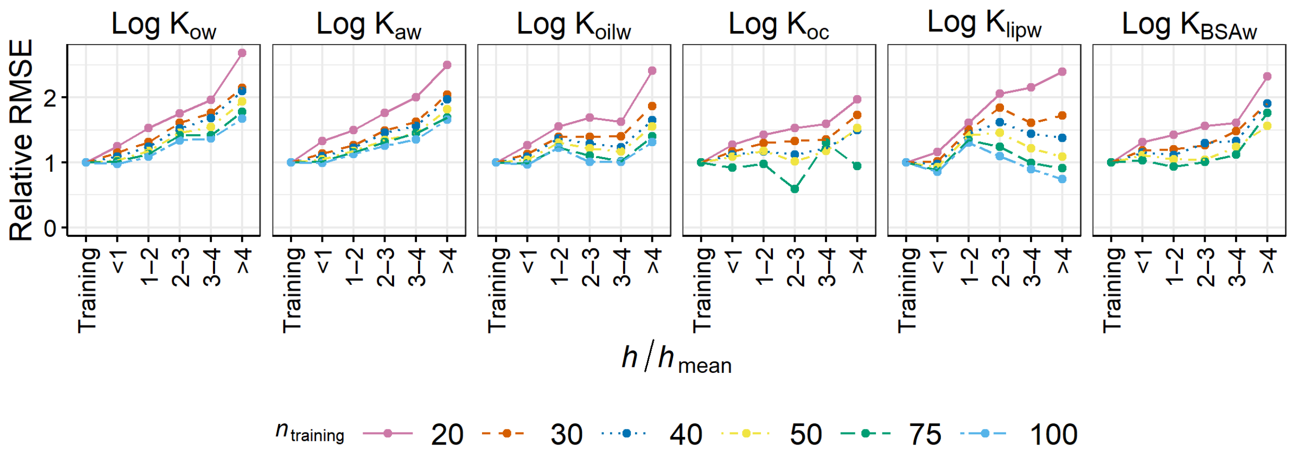


Figure S2. RMSEs of the test data, sorted according to $h/h_{\text{mean}}$, relative to the RMSE of the training data.

## SI-5 Prediction errors normalized to $SD_{training}$ plotted against $h$ (Test 1)

(A) Log $K_{ow}$

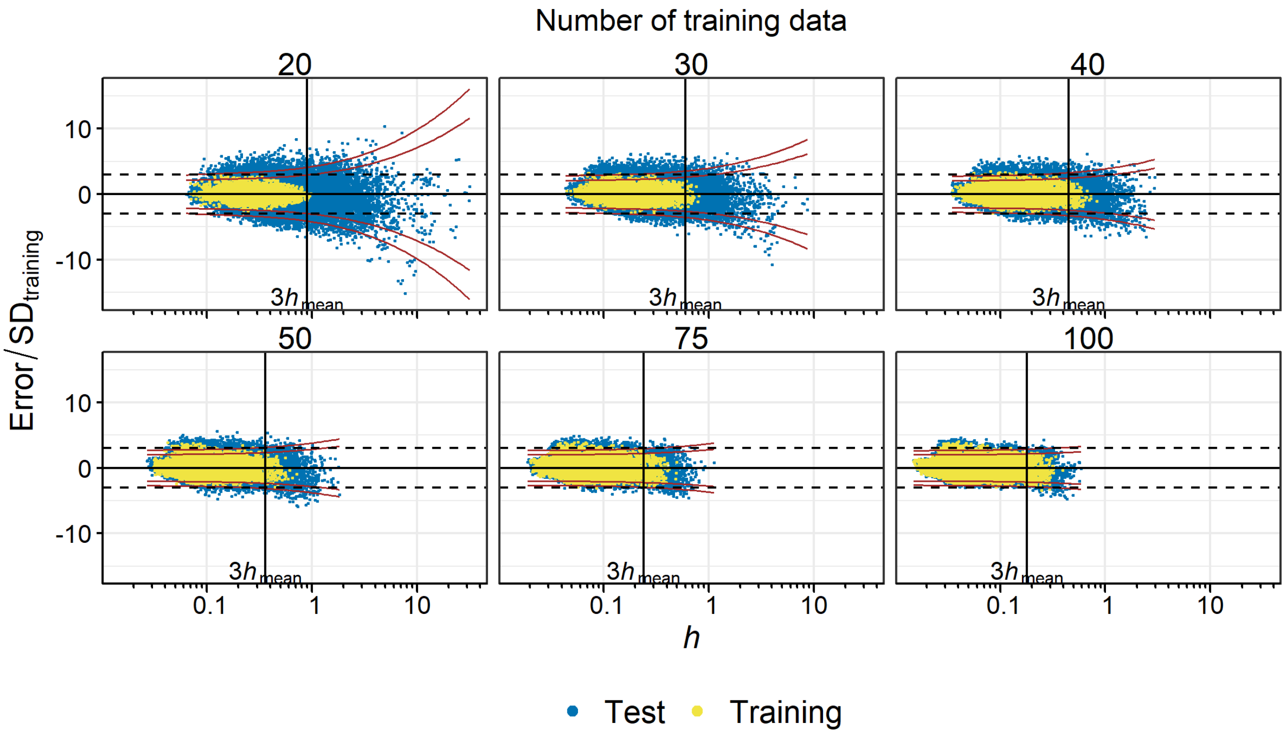


(B) Log $K_{aw}$

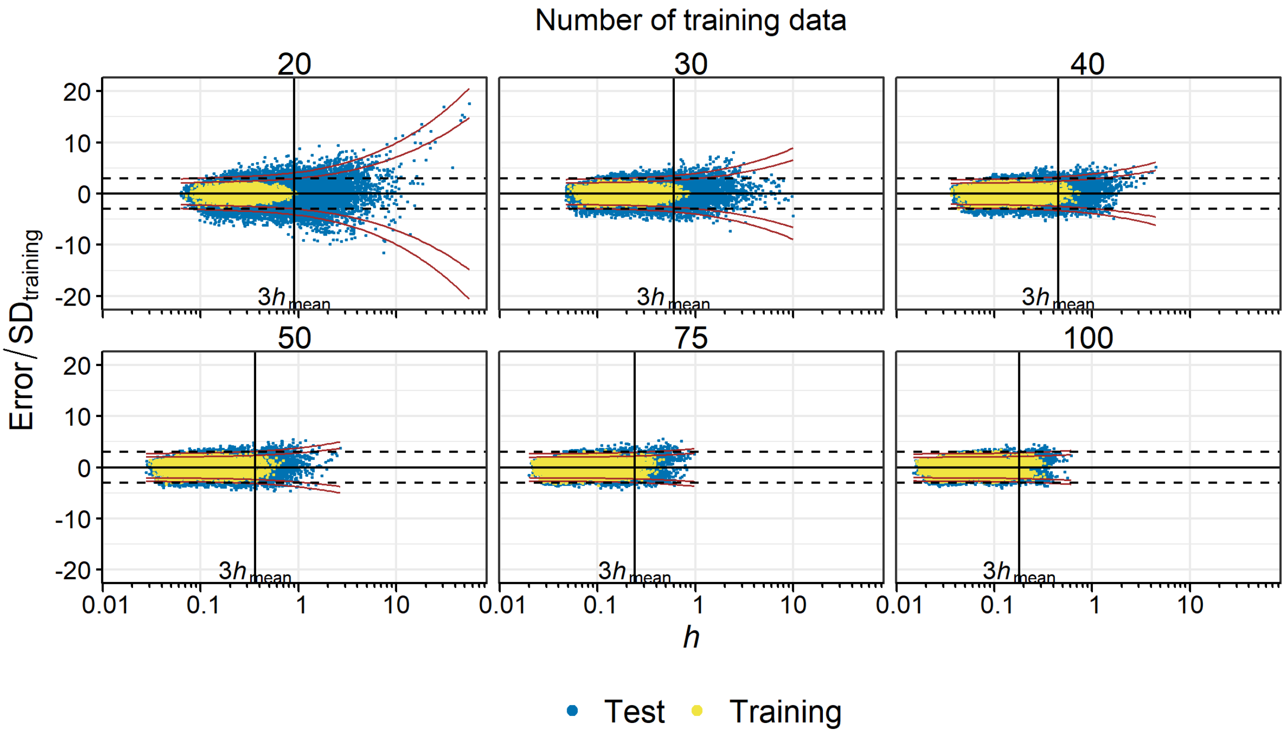


Figure S3 (A)–(F). Prediction errors normalized to $SD_{training}$ plotted against $h$. Results from 200 simulations are shown. The vertical line indicates $3h_{mean}$. The dashed horizontal lines indicate errors that are 3 times the $SD_{training}$. The curves indicate the 95% (inside) and 99% (outside) prediction intervals.

(C) Log $K_{oilw}$

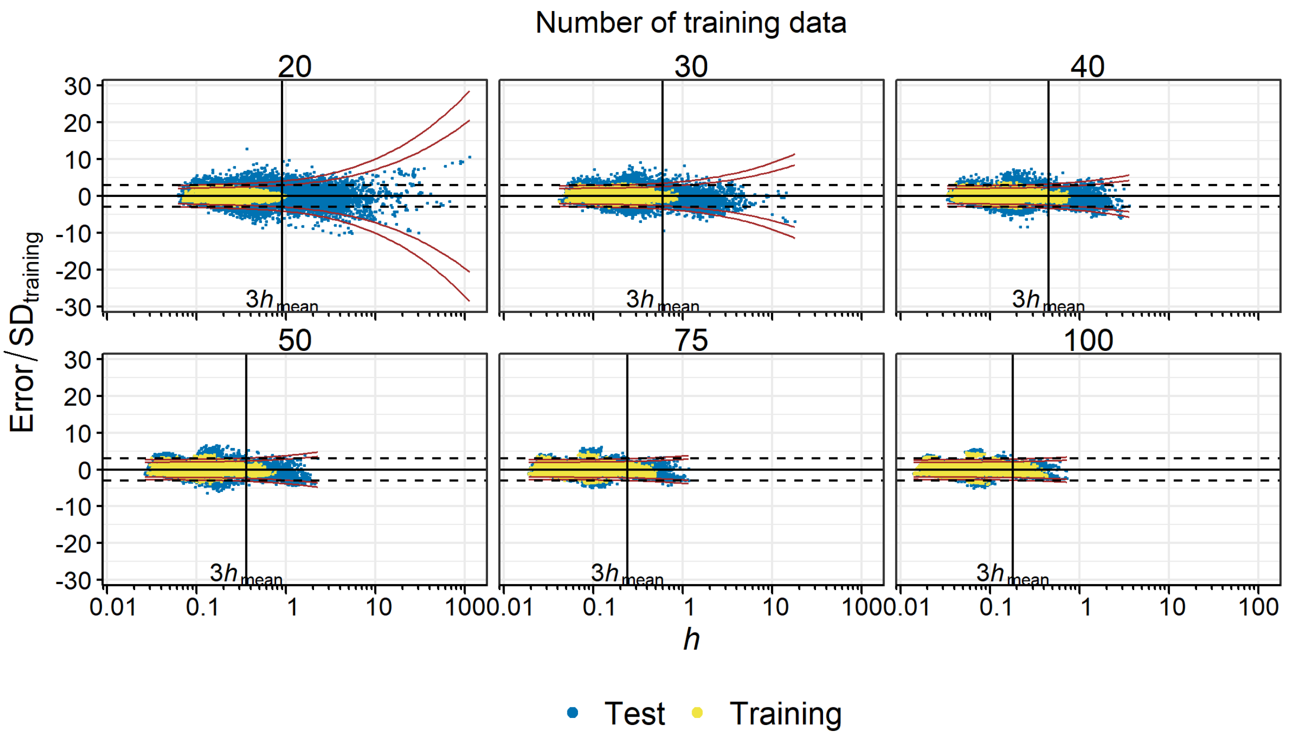


(D) Log $K_{oc}$

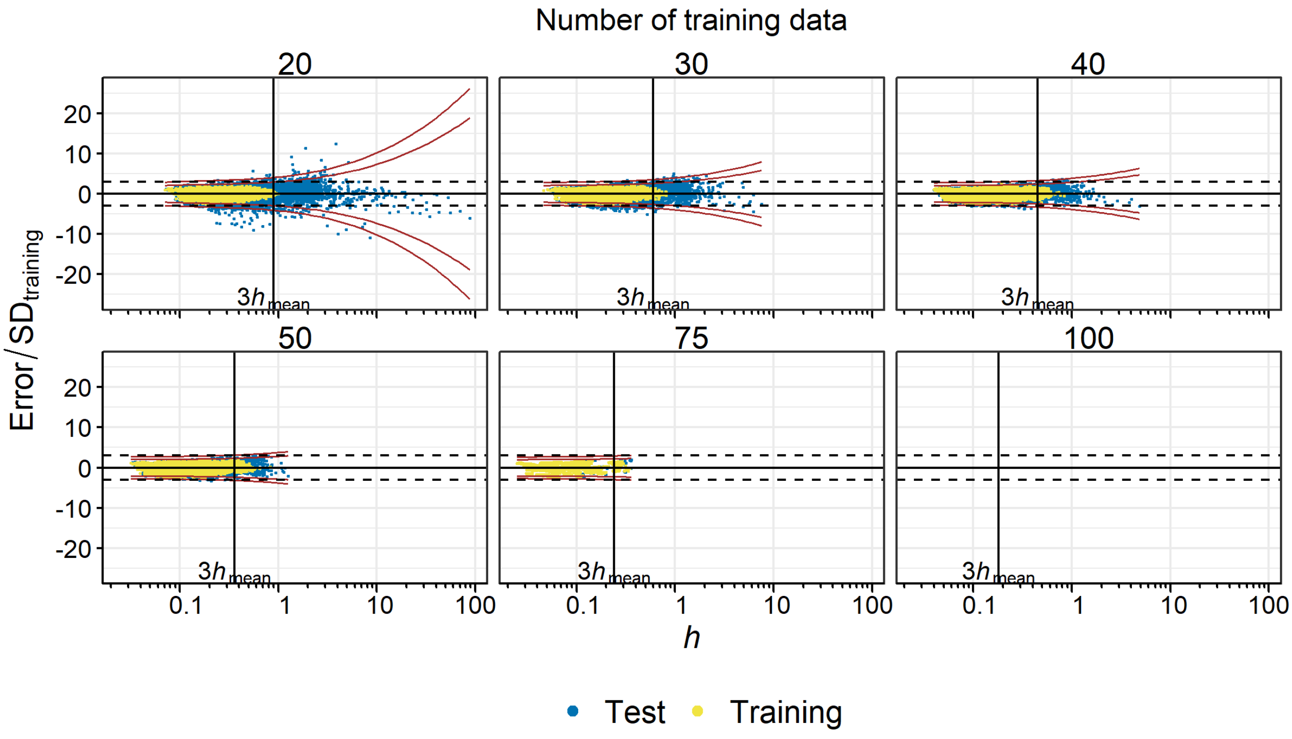


Figure S3. (continued)

(E) Log $K_{lipw}$

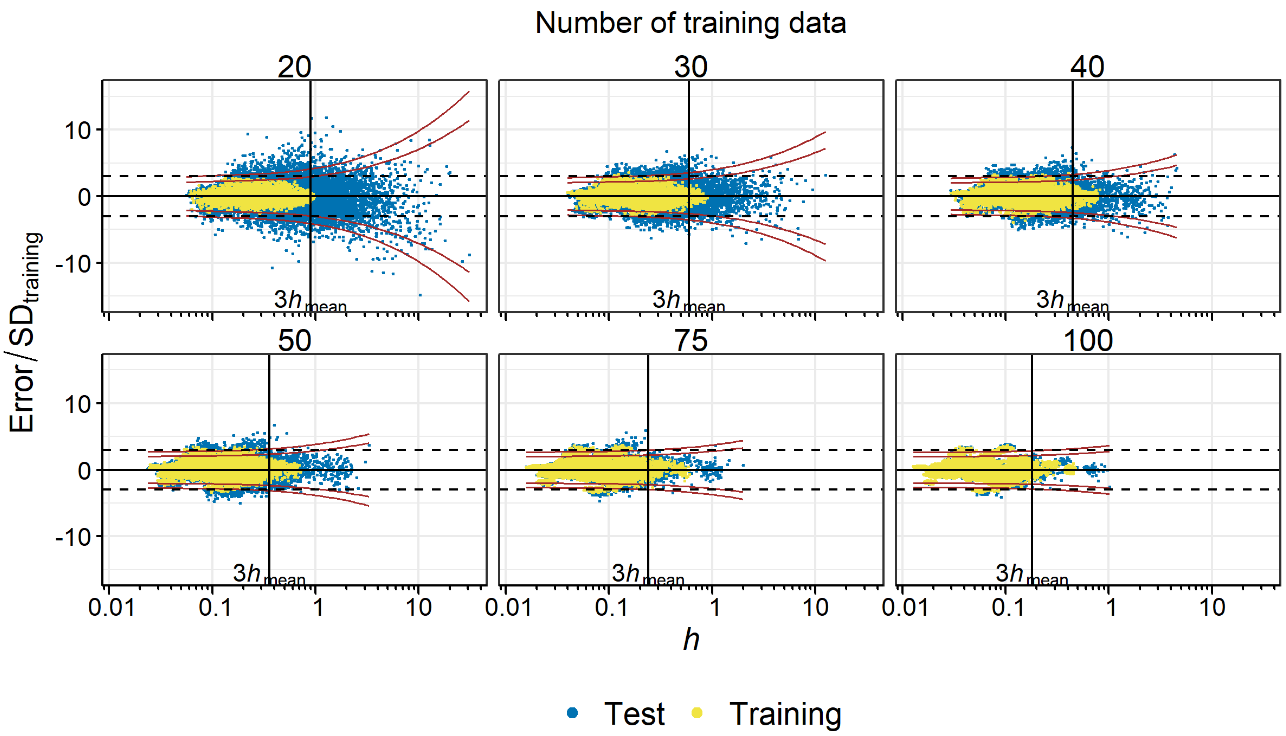


(F) Log $K_{BSAw}$

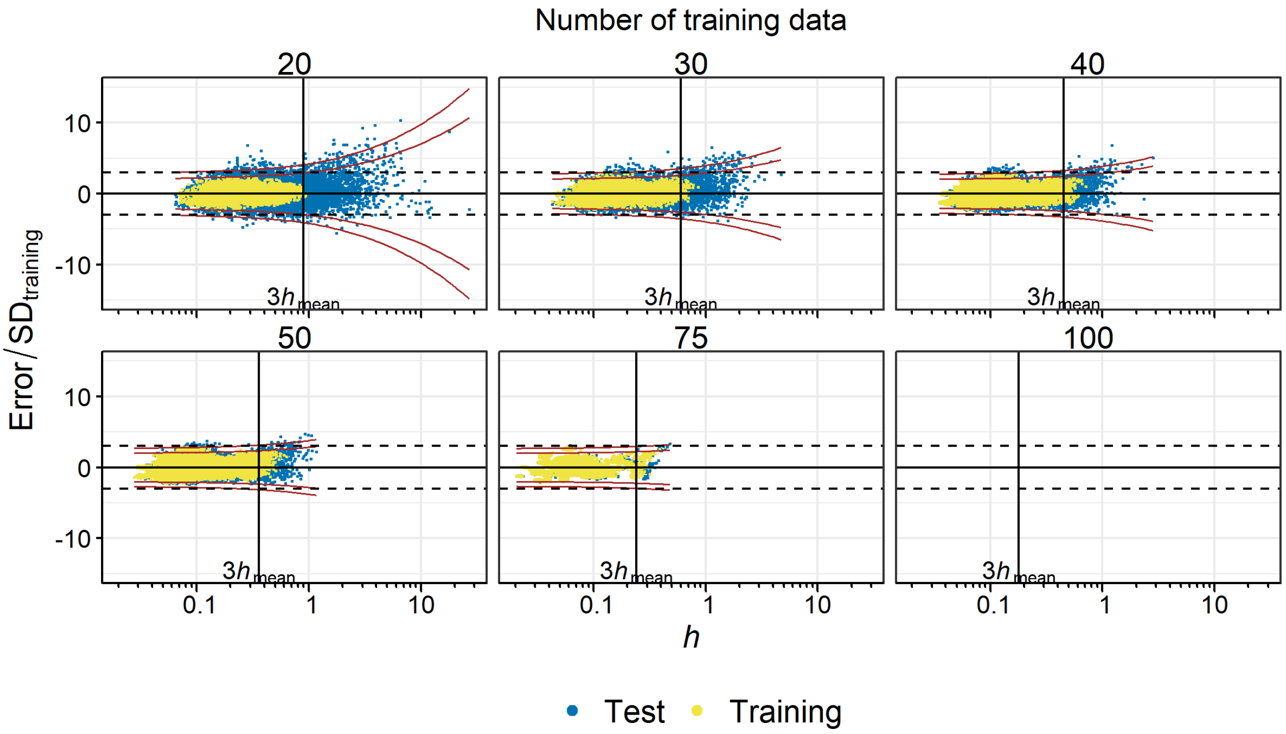


Figure S3. (continued)

## SI-6 Percentage of large prediction errors in interpolation and extrapolation (Test 1)

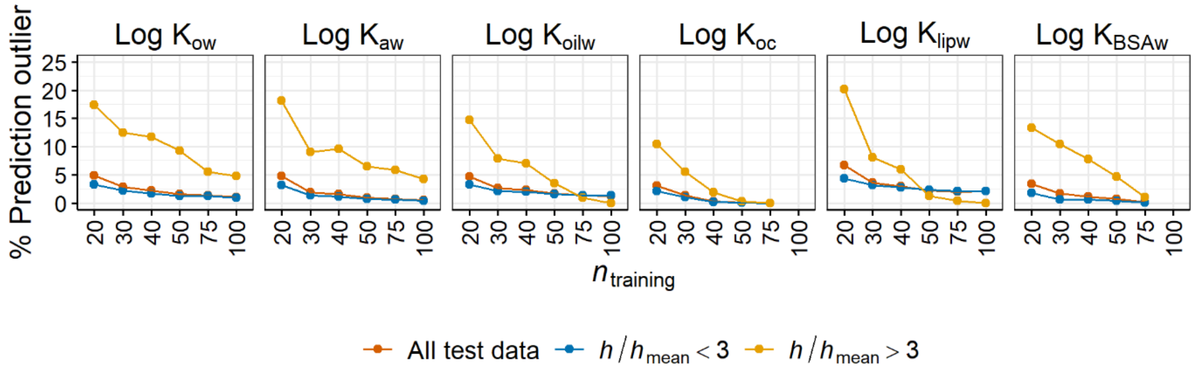


Figure S4. Percentage of prediction outliers, as defined by $|error/SD_{training}| > 3$, in the 200 repeated simulations.

## SI-7 Percentage of the test data for which predictions were within the given PIs (Test 1)

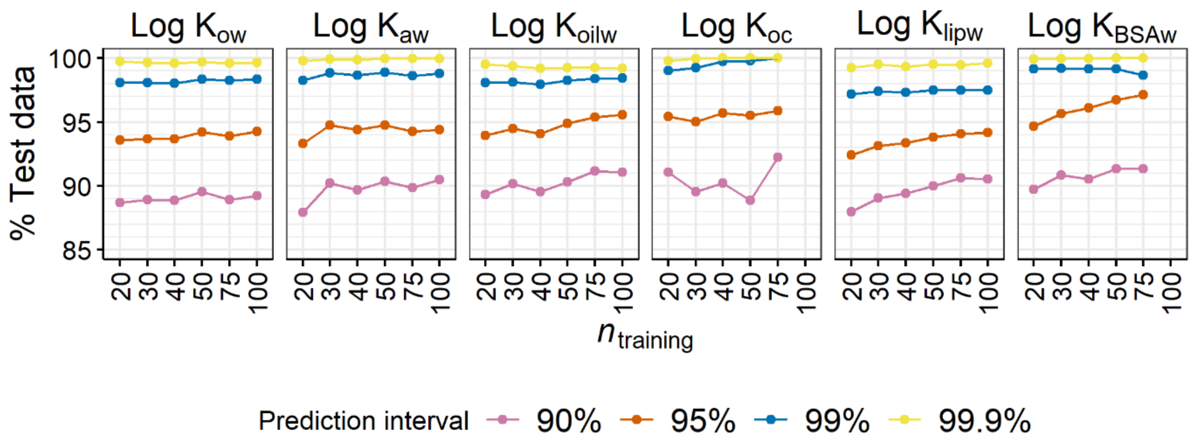


Figure S5. Percentage of the test data for which predictions were within the given PIs.

## SI-8 Prediction errors for PFASs and OSCs with <u>Equation 3 (*S, A, B, V, L*)</u>

(A) Log $K_{ow}$

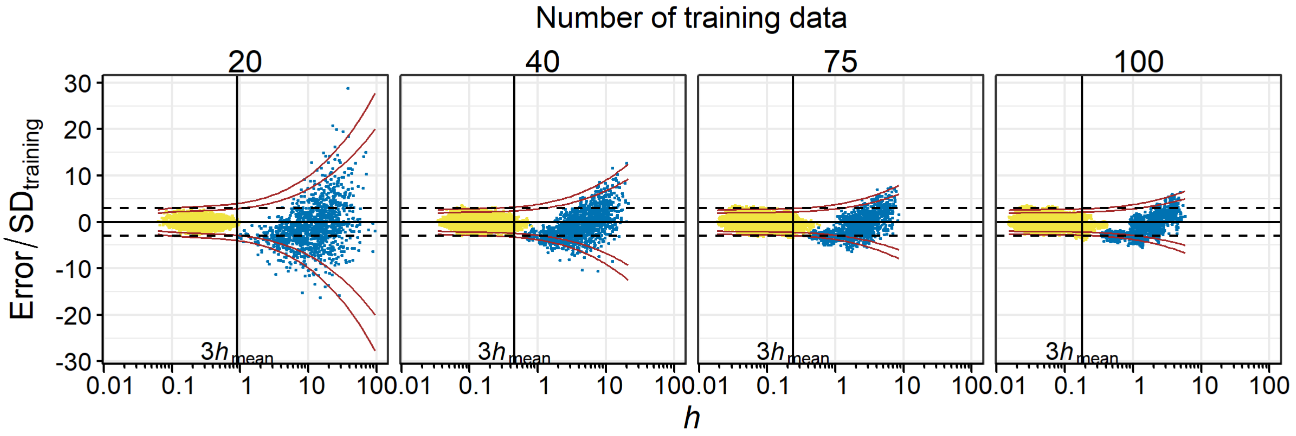


(B) Log $K_{aw}$

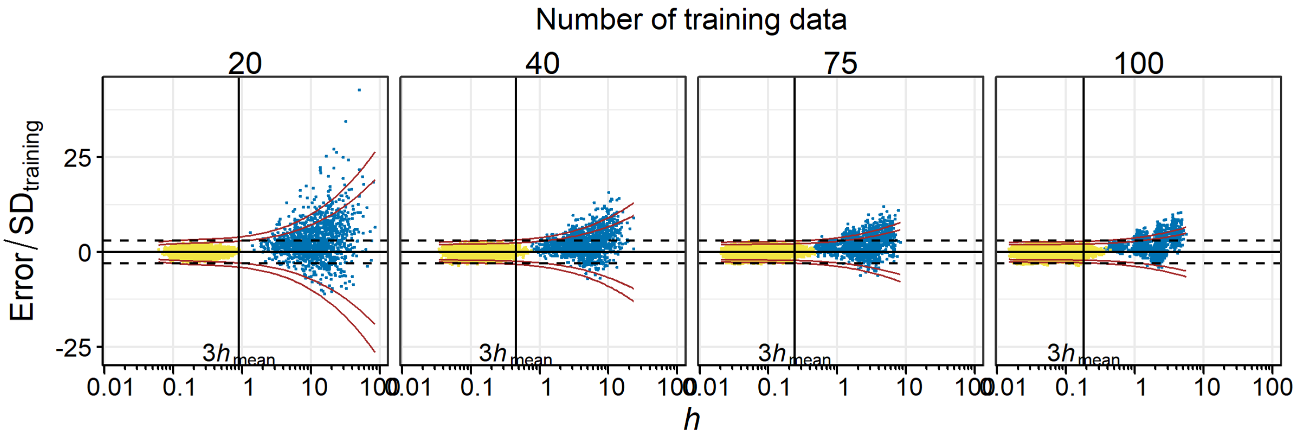


(C) Log $K_{oilw}$

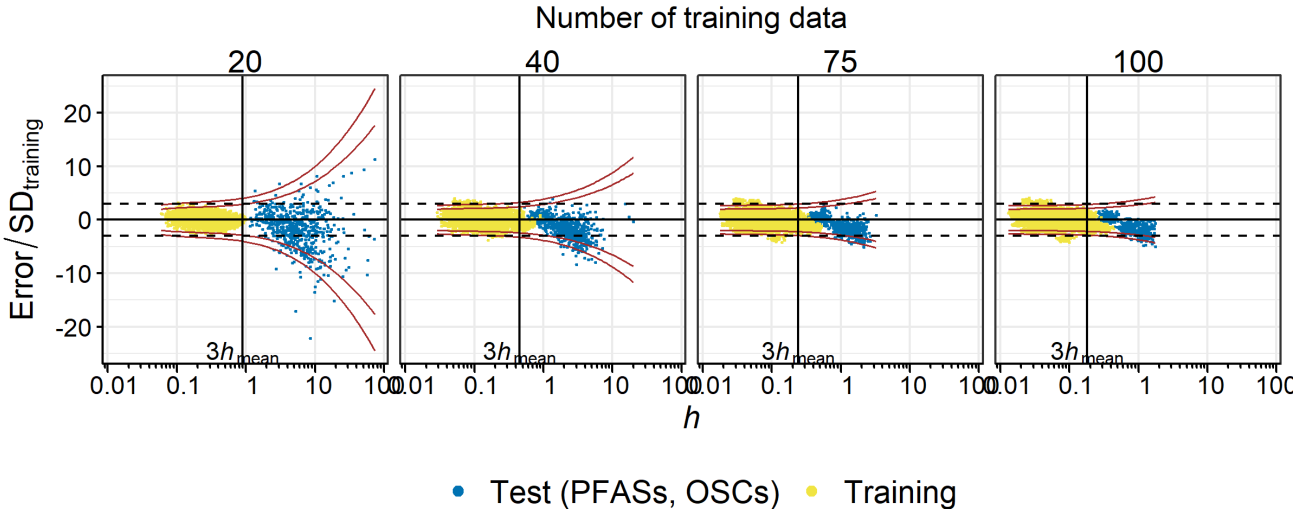


Figure S6 (A)–(F). Prediction errors for PFASs and OSCs normalized to $SD_{training}$ against $h$ (eq 3). Results from 200 simulations are shown. The vertical line indicates $3h_{mean}$. The dashed horizontal lines indicate errors that are 3 times the $SD_{training}$. The curves indicate the 95% (inside) and 99% (outside) PIs. **<u>Equation 3</u>** in the main text was used for this plot (see the text for more details).

(D) Log $K_{oc}$

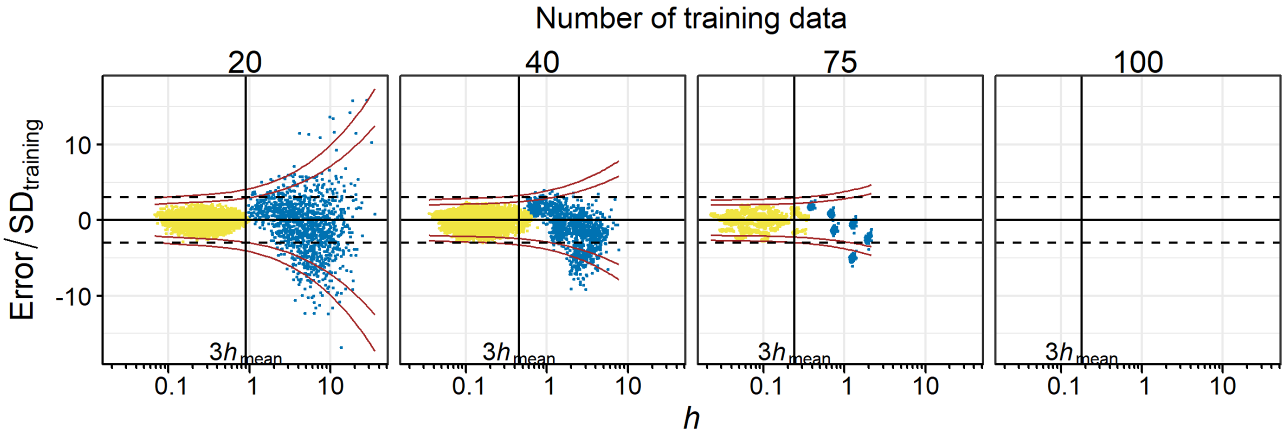


(E) Log $K_{lipw}$

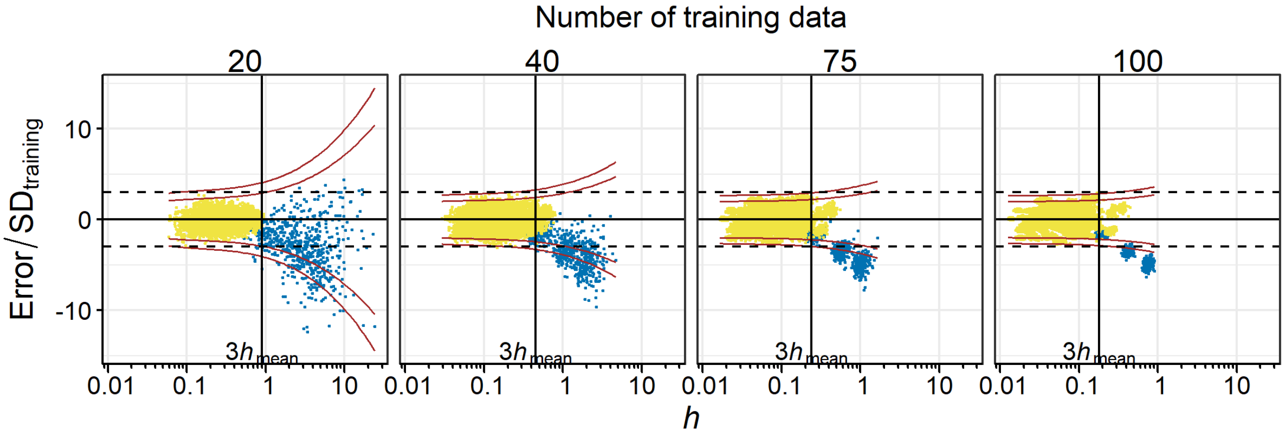


(F) Log $K_{BSAw}$

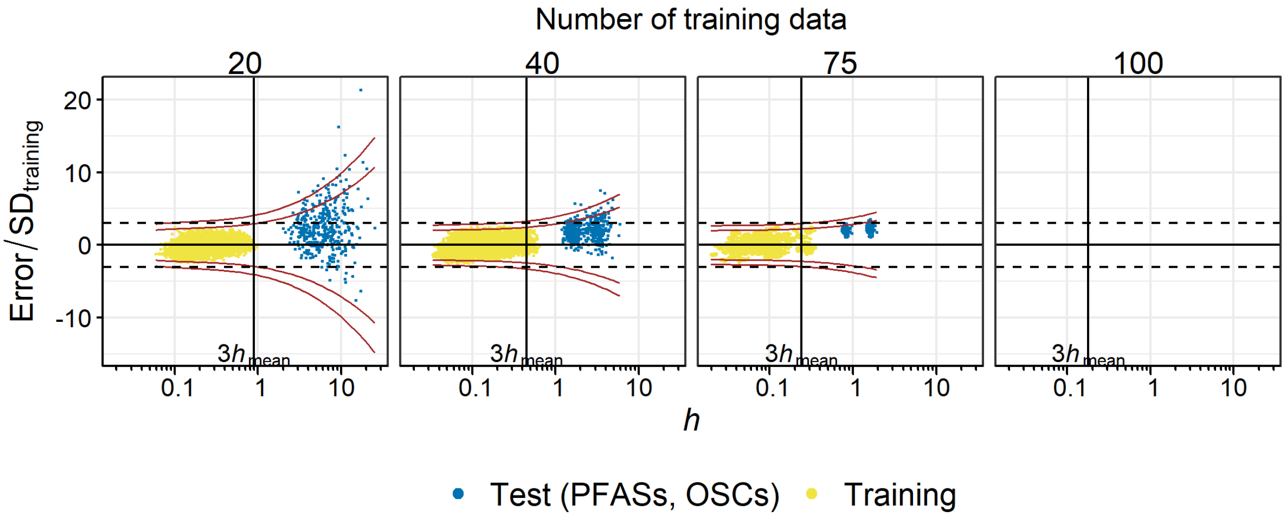


Figure S6. (continued)

## SI-9 Prediction errors for PFASs and OSCs with <u>Equation 1 (*E, S, A, B, V*)</u>

(A) Log $K_{ow}$

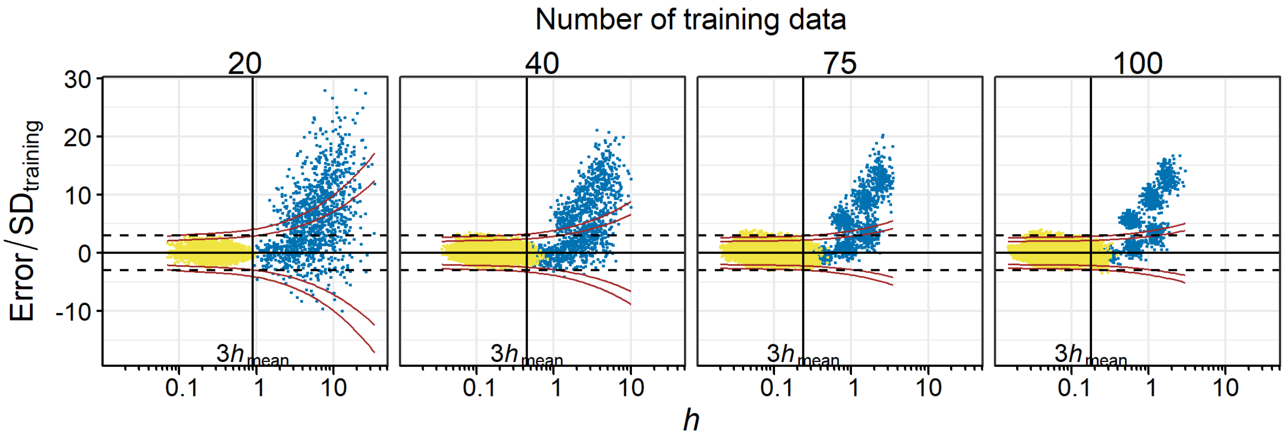


(B) Log $K_{aw}$

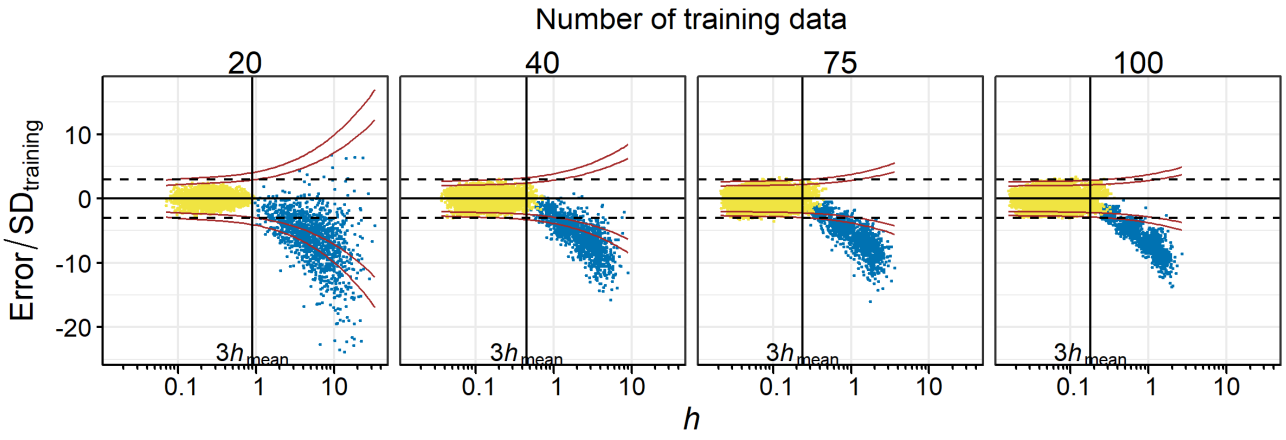


(C) Log $K_{oilw}$

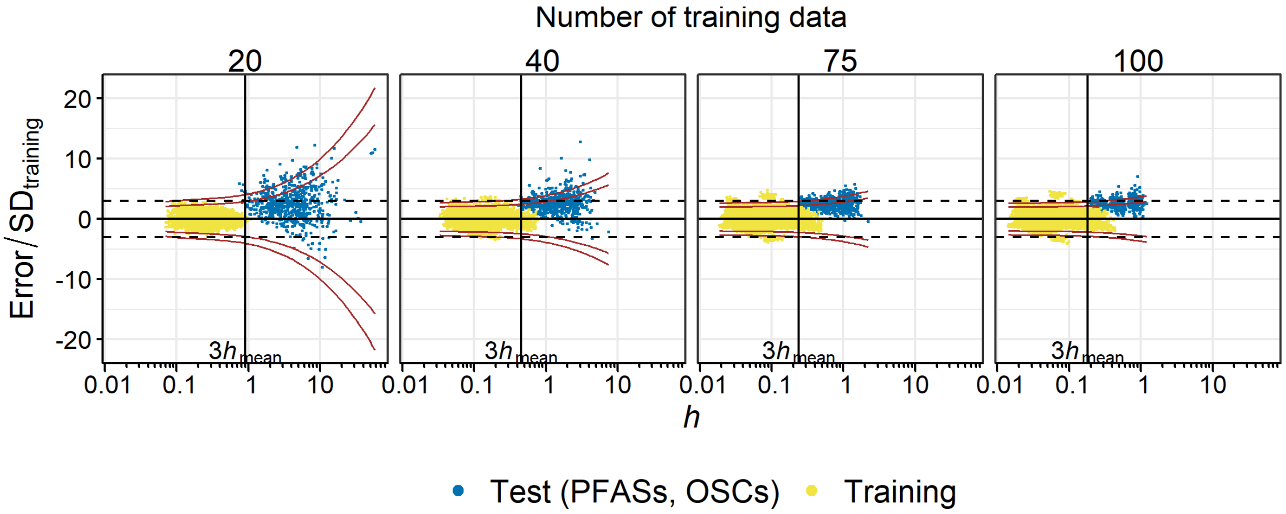


Figure S7 (A)–(F). Prediction errors for PFASs and OSCs normalized to $SD_{training}$ against $h$ (eq 1). Results from 200 simulations are shown. The vertical line indicates $3h_{mean}$. The dashed horizontal lines indicate errors that are 3 times the $SD_{training}$. The curves indicate the 95% (inside) and 99% (outside) PIs. **<u>Equation 1</u>** in the main text was used for this plot (see the text for more details).

(D) Log $K_{oc}$

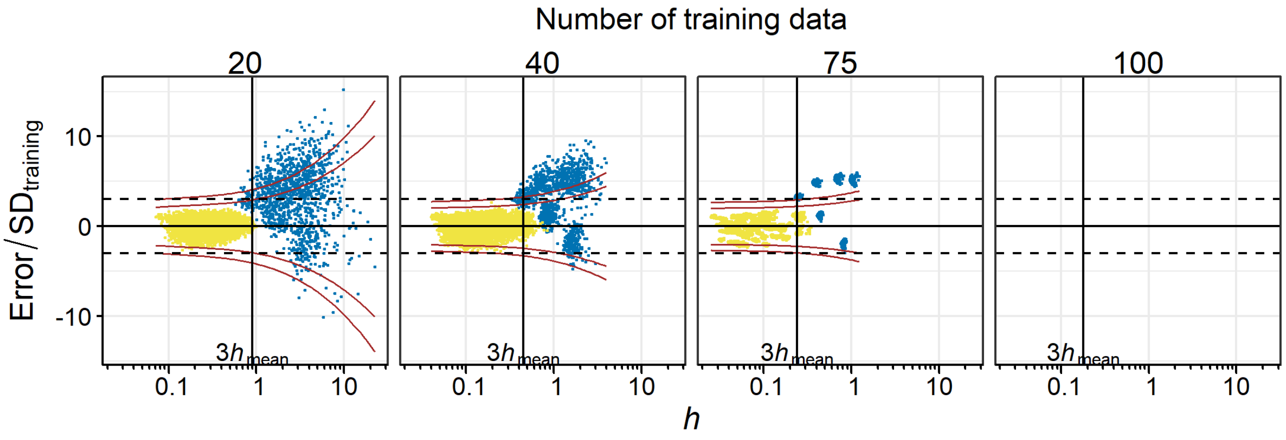


(E) Log $K_{lipw}$

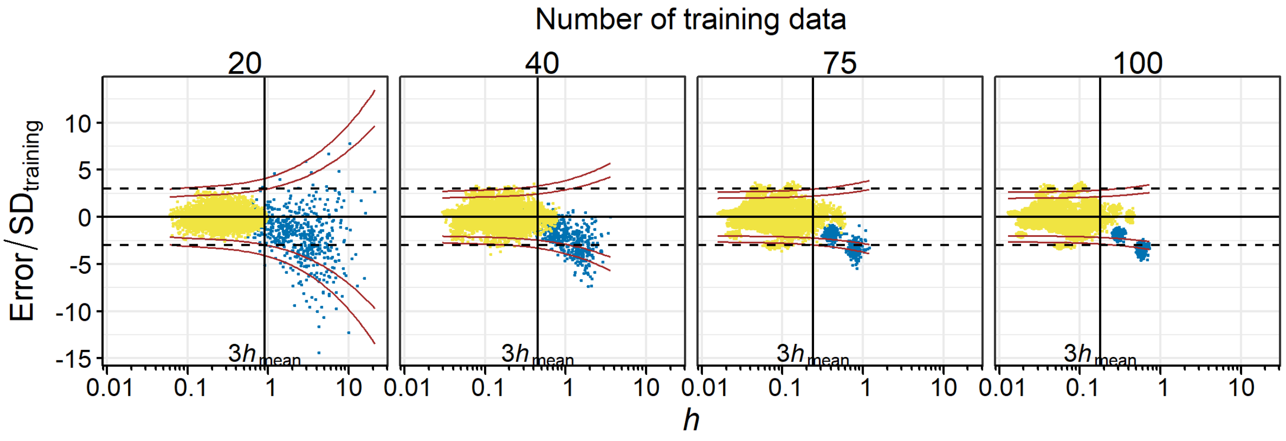


(F) Log $K_{BSAw}$

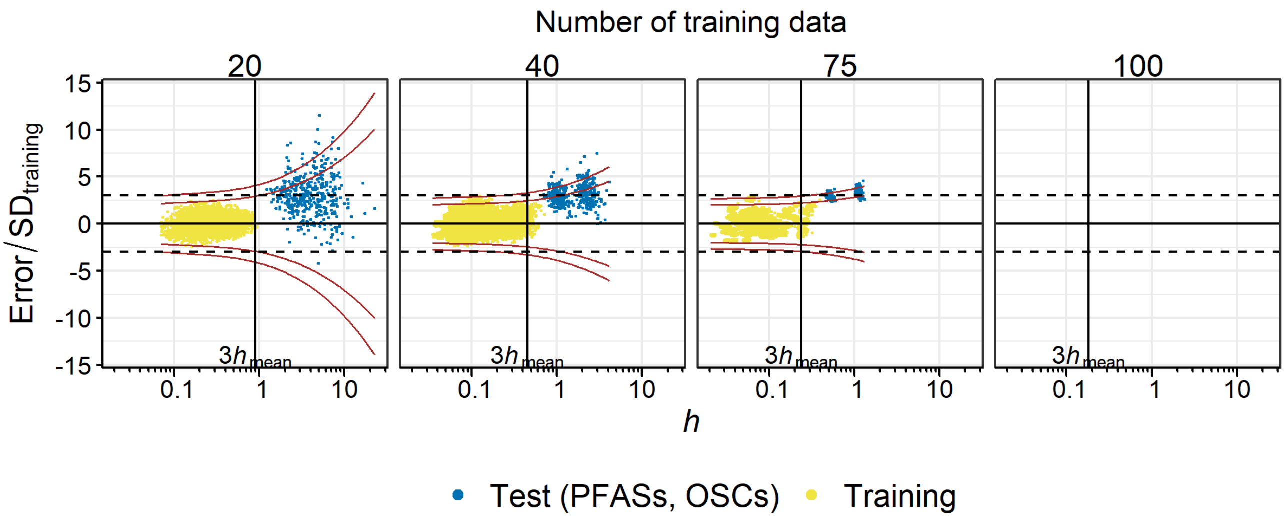


Figure S7. (continued)

## SI-10 Evaluation of literature PP-LFERs with leverages and prediction intervals for AD probes

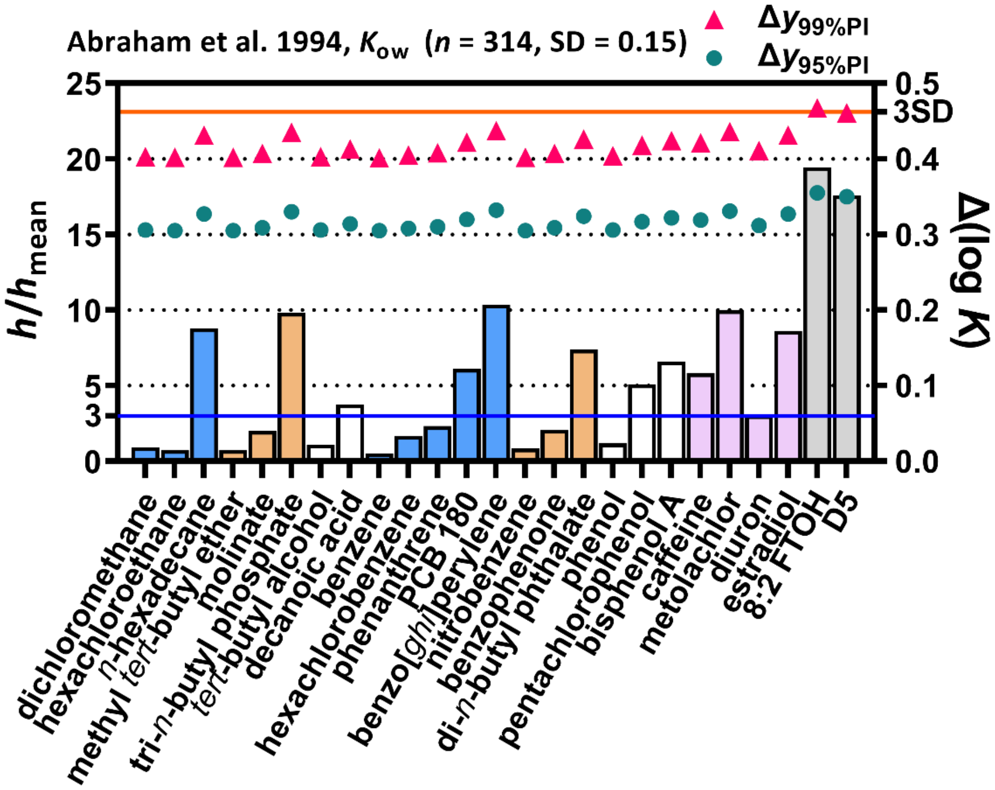


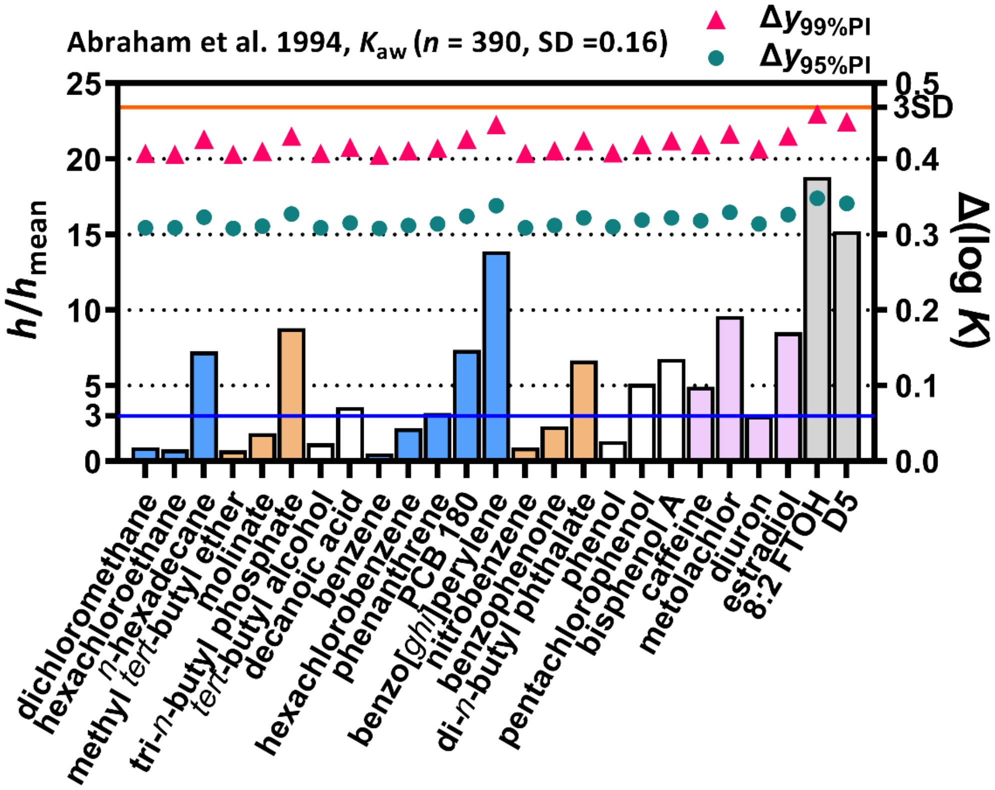


Figure S8. Leverage (bars) and PIs (triangles and circles) of 25 AD probes calculated with the training data sets of 10 PP-LFERs in the literature.[5-14] Solid horizontal lines indicate $h/h_{mean}$ = 3 and Δ(log *K*) = 3SD.

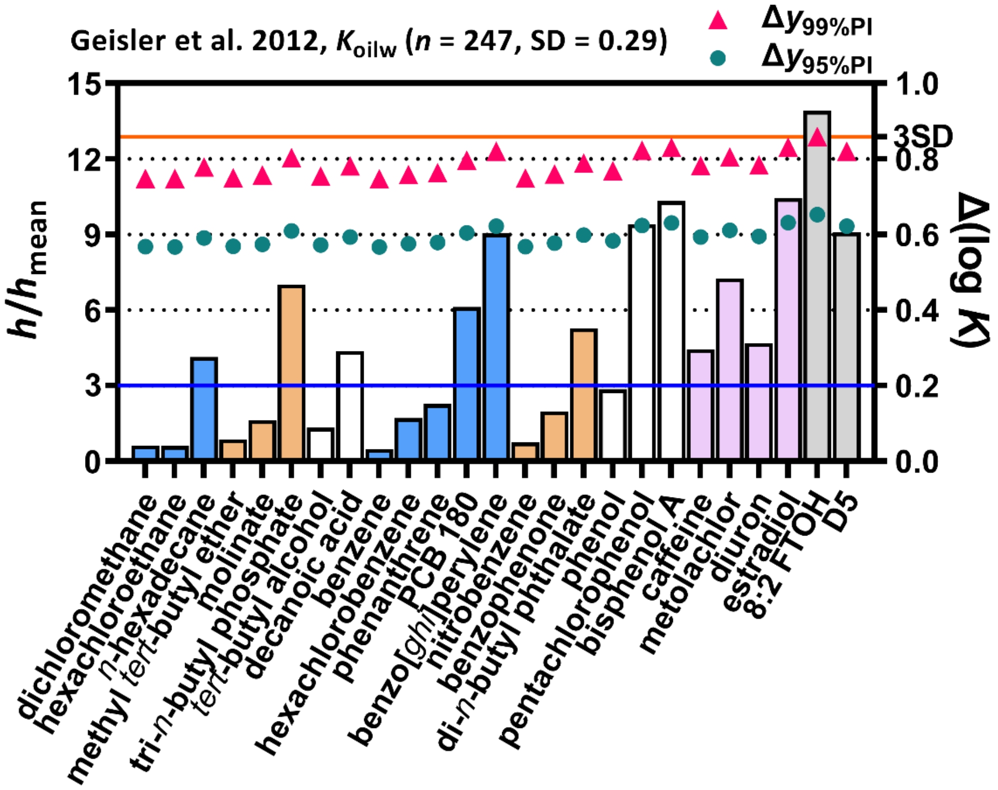


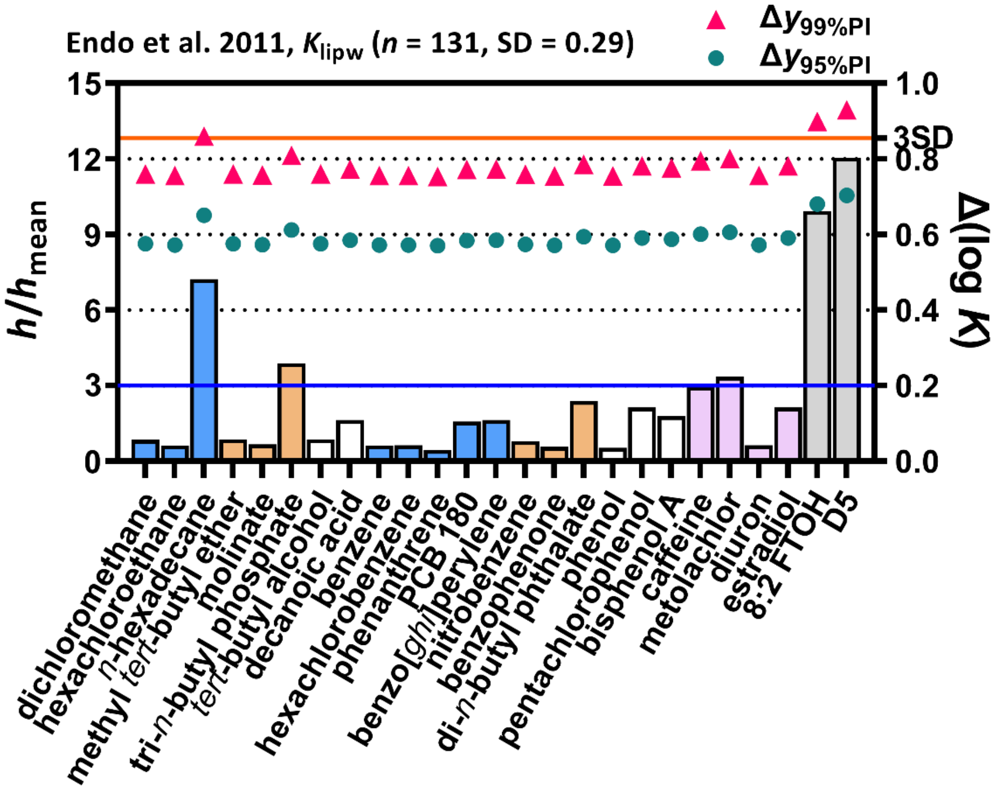


Figure S8. (continued)

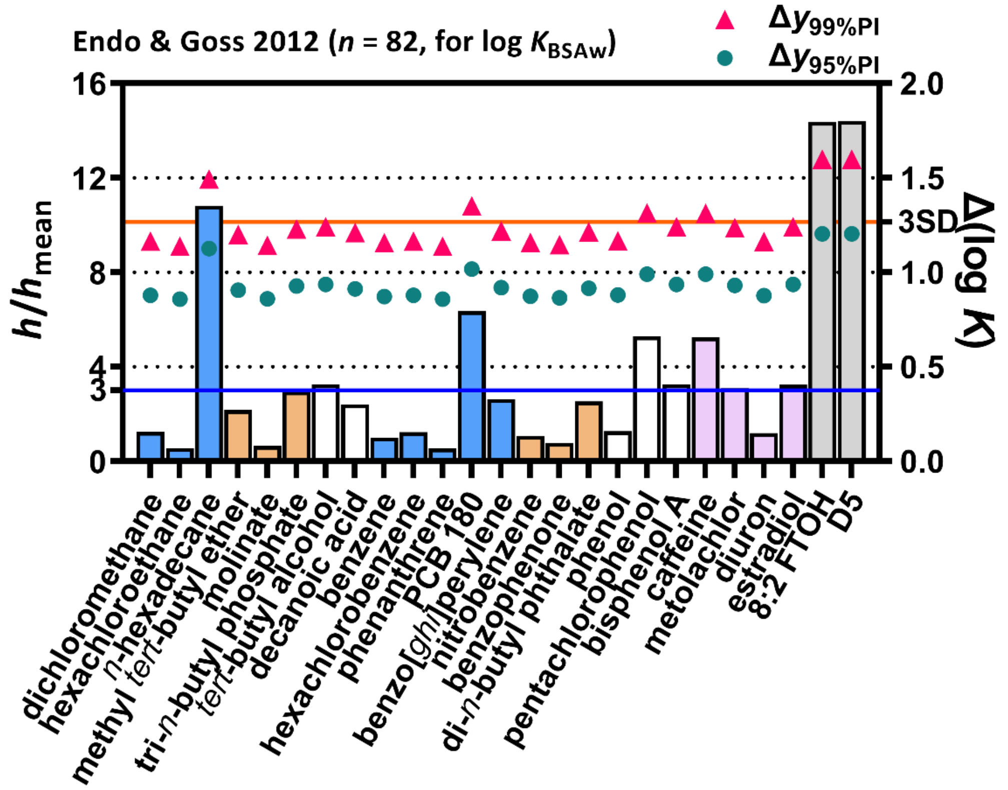


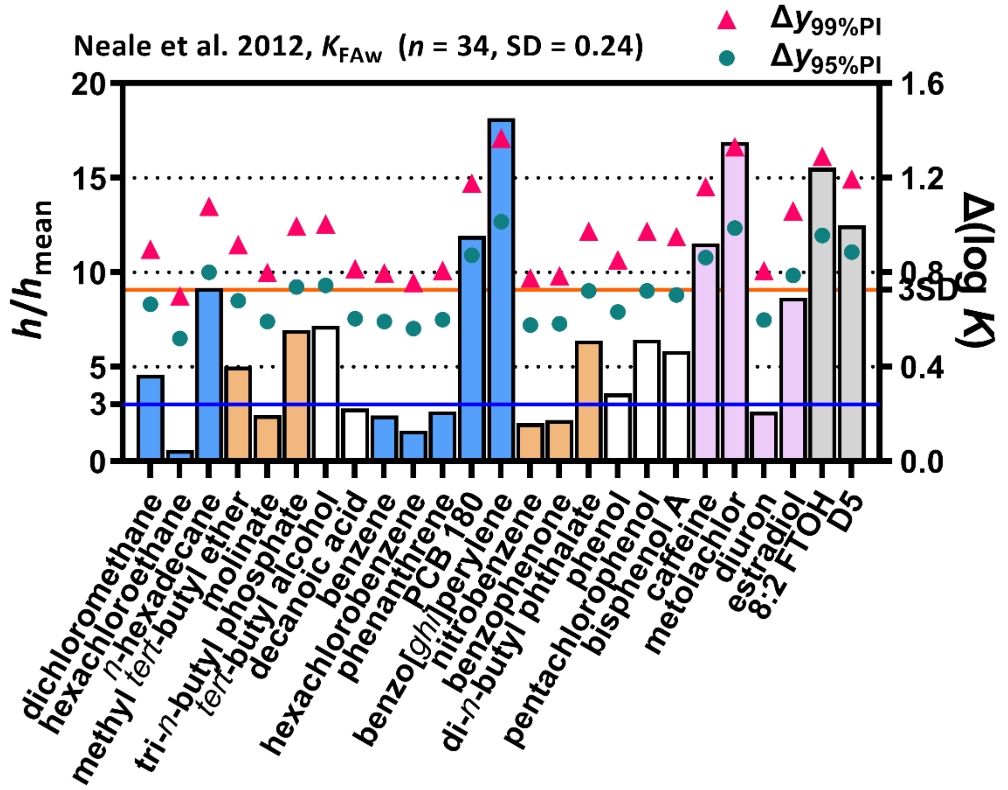


Figure S8. (continued)

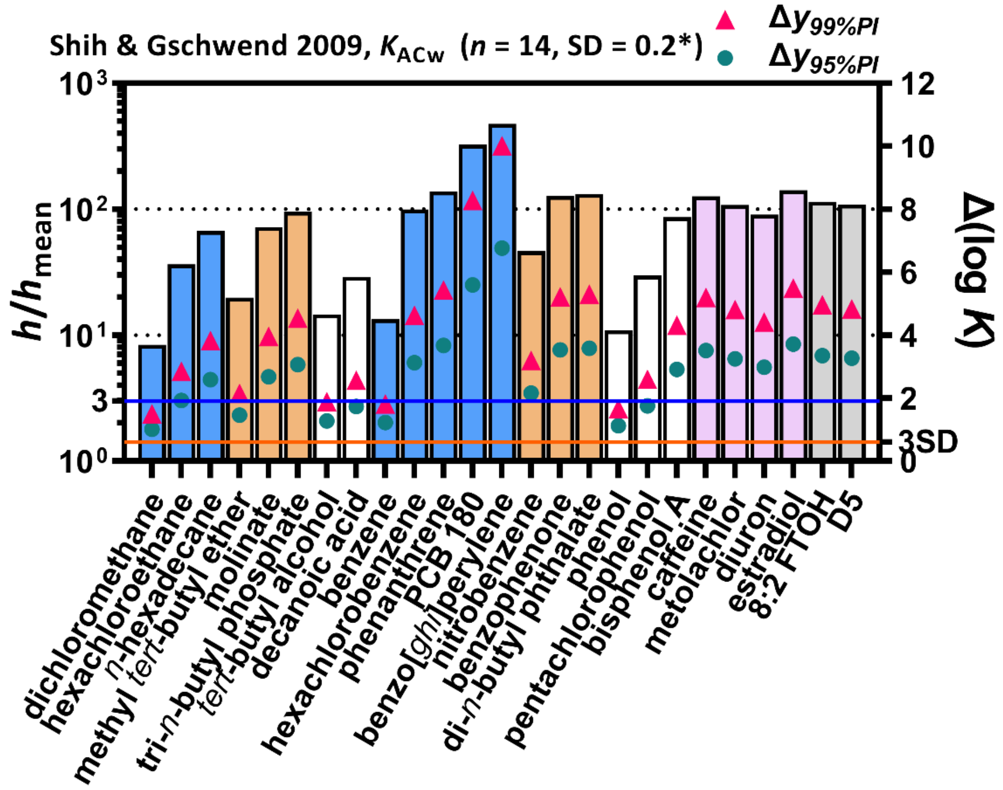


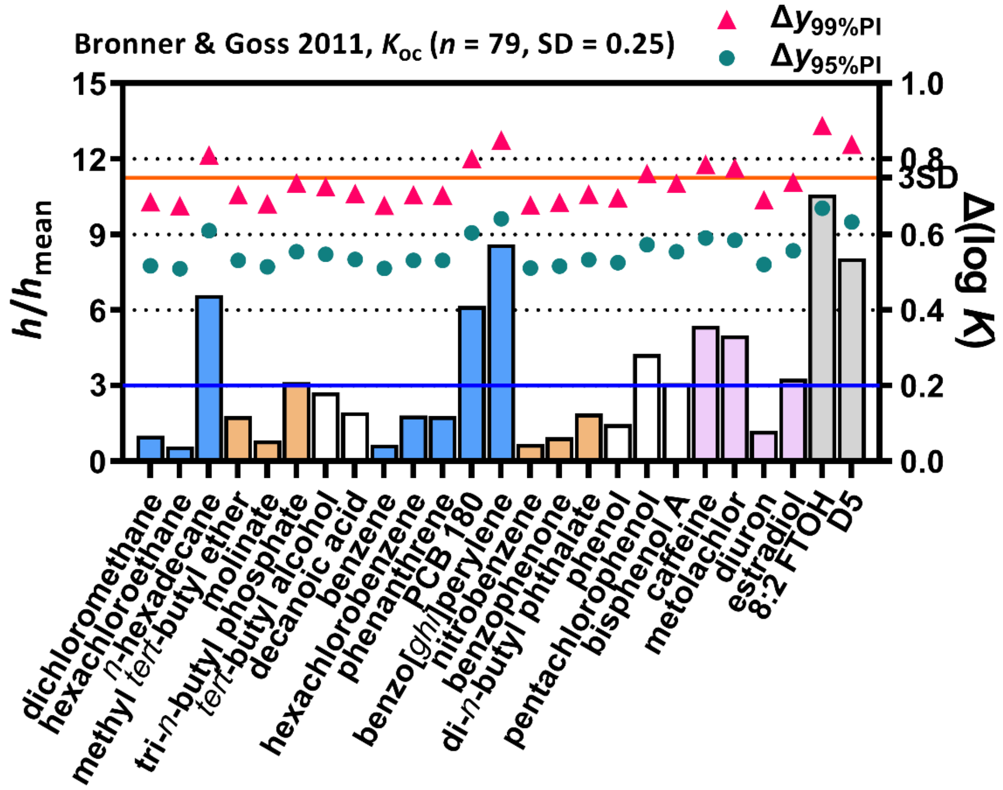


Figure S8. (continued)

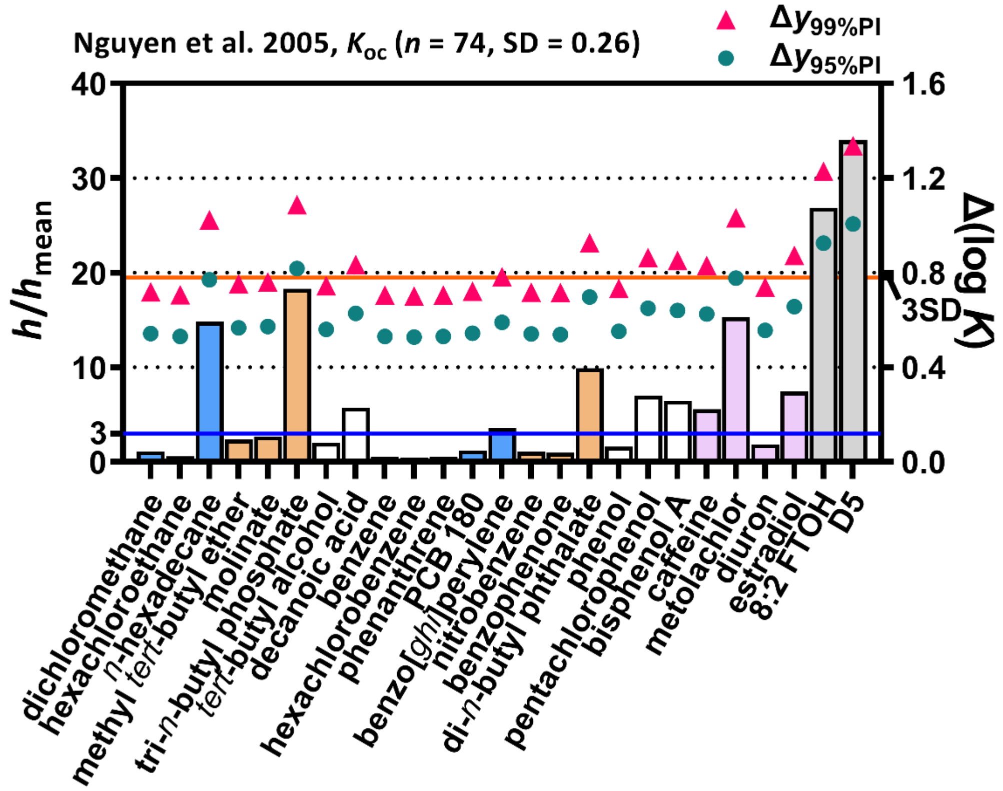


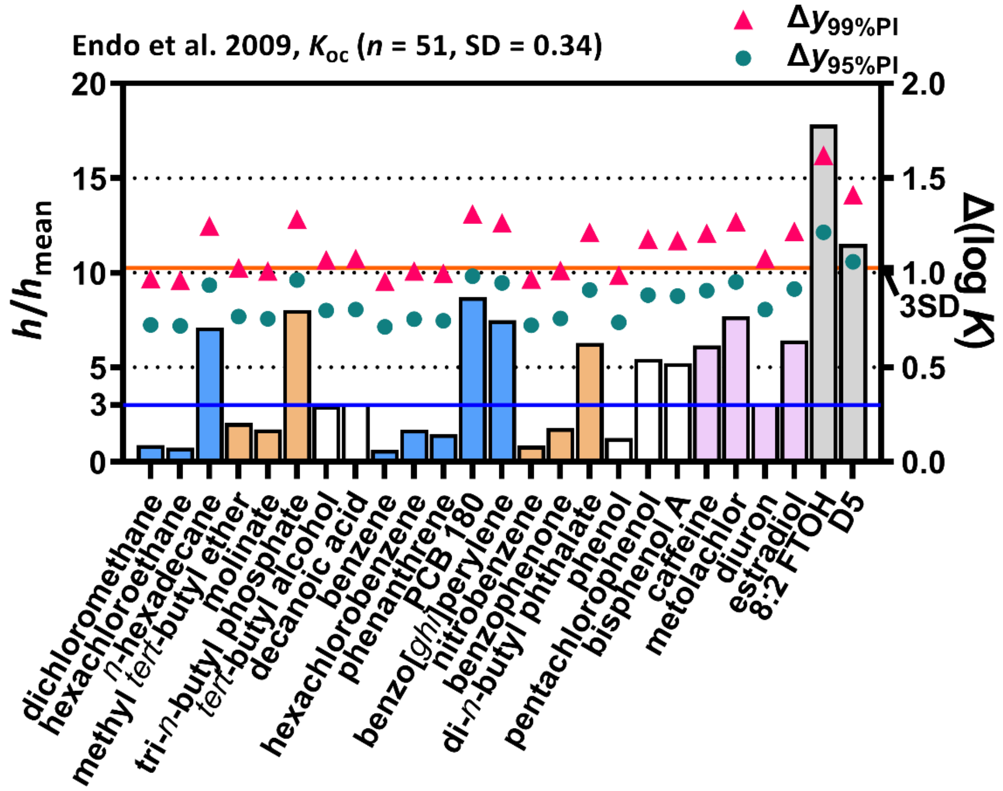


Figure S8. (continued)

**Additional discussion on the AD of PP-LFERs for log $K_{oc}$**

Three data sets for log $K_{oc}$ exhibiting different characters were compared in terms of their ADs (Figure S8). The data set of Bronner and Goss[8] was well-balanced, covering 18 AD probes within the $h/h_{mean} < 3$ limit. The large hydrophobic compounds exhibited $h/h_{mean} > 5$, because such compounds were absent in the data set. Bronner and Goss used a soil column chromatography method to measure $K_{oc}$, but the method is unsuitable for highly hydrophobic compounds, which would be strongly retained by the soil column. The data from Nguyen et al.[13] for $K_{oc}$ exhibited the opposite characteristics. The data set covered nonpolar aromatic compounds well, whereas polar compounds (e.g., tri-*n*-butyl phosphate, metolachlor) exhibited large $h/h_{mean}$ and Δ(log $K$) values. The data set of Nguyen et al. was a selection of literature data, which were predominated with nonpolar aromatic compounds. The data set of Endo et al.[14] resulted in relatively high $h/h_{mean}$ and Δ(log $K$) values for large compounds, irrespective of polarity, reflecting the types of compounds that were not included in the data set.

**References**


1. Netzeva, T. I.; Worth, A.; Aldenberg, T.; Benigni, R.; Cronin, M. T.; Gramatica, P.; Jaworska, J. S.; Kahn, S.; Klopman, G.; Marchant, C. A.; Myatt, G.; Nikolova-Jeliazkova, N.; Patlewicz, G. Y.; Perkins, R.; Roberts, D.; Schultz, T.; Stanton, D. W.; van de Sandt, J. J.; Tong, W.; Veith, G.; Yang, C., Current status of methods for defining the applicability domain of (quantitative) structure-activity relationships. The report and recommendations of ECVAM Workshop 52. *ATLA Altern. Lab. Anim.* **2005,** *33*, (2), 155-73.
2. Jaworska, J.; Nikolova-Jeliazkova, N.; Aldenberg, T., QSAR Applicability Domain Estimation by Projection of the Training Set in Descriptor Space: A Review. *ATLA Altern. Lab. Anim.* **2005,** *33*, (5), 445-459.
3. Gramatica, P., Principles of QSAR models validation: internal and external. *QSAR Comb Sci.* **2007,** *26*, (5), 694-701.
4. Gramatica, P.; Giani, E.; Papa, E., Statistical external validation and consensus modeling: A QSPR case study for $K_{oc}$ prediction. *J. Mol. Graph. Model.* **2007,** *25*, (6), 755-766.
5. Abraham, M. H.; Chadha, H. S.; Whiting, G. S.; Mitchell, R. C., Hydrogen bonding. 32. An analysis of water-octanol and water-alkane partitioning and the ∆log P parameter of seiler. *J. Pharm. Sci.* **1994,** *83*, (8), 1085-100.
6. Abraham, M. H.; Andonian-Haftvan, J.; Whiting, G. S.; Leo, A.; Taft, R. S., Hydrogen bonding. Part 34. The factors that influence the solubility of gases and vapors in water at 298 K, and a new method for its determination. *J. Chem. Soc. Perkin Trans. 2* **1994**, (8), 1777-91.
7. Geisler, A.; Endo, S.; Goss, K.-U., Partitioning of Organic Chemicals to Storage Lipids: Elucidating the Dependence on Fatty Acid Composition and Temperature. *Environ. Sci. Technol.* **2012,** *46*, (17), 9519-9524.
8. Bronner, G.; Goss, K.-U., Predicting sorption of pesticides and other multifunctional organic chemicals to soil organic carbon. *Environ. Sci. Technol.* **2011,** *45*, (4), 1313-1319.
9. Endo, S.; Escher, B. I.; Goss, K.-U., Capacities of Membrane Lipids to Accumulate Neutral Organic Chemicals. *Environ. Sci. Technol.* **2011,** *45*, (14), 5912-5921.
10. Endo, S.; Goss, K.-U., Serum Albumin Binding of Structurally Diverse Neutral Organic Compounds: Data and Models. *Chem. Res. Toxicol.* **2011,** *24*, (12), 2293-2301.
11. Neale, P. A.; Escher, B. I.; Goss, K.-U.; Endo, S., Evaluating dissolved organic carbon–water partitioning using polyparameter linear free energy relationships: Implications for the fate of disinfection by-products. *Water Res.* **2012,** *46*, (11), 3637-3645.
12. Shih, Y.-h.; Gschwend, P. M., Evaluating Activated Carbon−Water Sorption Coefficients of Organic Compounds Using a Linear Solvation Energy Relationship Approach and Sorbate Chemical Activities. *Environ. Sci. Technol.* **2009,** *43*, (3), 851-857.
13. Nguyen, T. H.; Goss, K.-U.; Ball, W. P., Polyparameter linear free energy relationships for estimating the equilibrium partition of organic compounds between water and the natural organic matter in soils and sediments. *Environ. Sci. Technol.* **2005,** *39*, (4), 913-924.
14. Endo, S.; Grathwohl, P.; Haderlein, S. B.; Schmidt, T. C., LFERs for soil organic carbon-water distribution coefficients ($K_{OC}$) at environmentally relevant sorbate concentrations. *Environ. Sci.*

*Technol.* **2009,** *43*, (9), 3094-3100.
